# Supplementary material for: Preliminary Assessment of Microbial Community Structure of Wind-Tidal Flats in the Laguna Madre, Texas, USA
Source: Biology (Basel). 2020 Jul 22;9(8):183. doi: 10.3390/biology9080183 (PMC7464120; doi:10.3390/biology9080183)
Supplement: Supplementary file 1 [file biology-09-00183-s001.zip › biology-866877-supplementary materials/20200507_OTU tables.pdf]

LMFS-W	LMFS-M	LMFS-D	NH-W	NH-M	NH-D	KRN-W	KRN-M	KRN-D	KRS-D	KRS-M	KRS-W	Domain	Phylum	Class	Order	Family	Genus	Species		strain
0	0	0	0	0	0	0	0	7	0	0	0	Bacteria	Acetothermia	Acetothermiia	Acetothermiia	unclassified_Bacteria				
0	0	0	0	0	8	0	0	0	0	0	0	Bacteria	Acidobacteriota	Holophagae	Acanthopleuribacterales	Acanthopleuribacteraceae	Acanthopleuribacter	Acanthopleuribacter	sp.	
38	0	0	0	0	0	0	0	0	0	0	0	Bacteria	Acidobacteriota	Holophagae	Acanthopleuribacterales	Acanthopleuribacteraceae	Acanthopleuribacter	Acanthopleuribacter	sp.	
0	0	0	0	14	0	20	60	22	0	31	0	Bacteria	Acidobacteriota	Acidobacteriae	unclassified_Bacteria	unclassified_Bacteria	PAUC26f			
66	0	0	0	21	0	105	221	117	0	29	68	Bacteria	Acidobacteriota	Thermoanaerobaculia	Thermoanaerobaculales	Thermoanaerobaculaceae	Subgroup 10			
0	0	0	0	19	0	0	35	90	0	0	0	Bacteria	Acidobacteriota	Thermoanaerobaculia	Thermoanaerobaculales	Thermoanaerobaculaceae	Subgroup 10			YC-LK-LKJ13
0	0	0	0	0	0	0	0	3	0	0	0	Bacteria	Acidobacteriota	Thermoanaerobaculia	Thermoanaerobaculales	Thermoanaerobaculaceae	Subgroup 10			
0	0	0	0	0	0	0	37	0	0	0	0	Bacteria	Acidobacteriota	Thermoanaerobaculia	Thermoanaerobaculales	Thermoanaerobaculaceae	Subgroup 10	Acidobacterium	sp.	
0	0	0	0	29	0	0	0	0	0	0	0	Bacteria	Acidobacteriota	Thermoanaerobaculia	Thermoanaerobaculales	Thermoanaerobaculaceae	Subgroup 10			
0	0	0	0	0	0	68	0	82	0	52	30	Bacteria	Acidobacteriota	Acidobacteriae	unclassified_Bacteria	unclassified_Bacteria	Subgroup 21			
0	0	0	0	0	0	0	0	0	0	0	15	Bacteria	Acidobacteriota	Acidobacteriae	unclassified_Bacteria	unclassified_Bacteria	Subgroup 22			
0	0	0	0	13	63	30	47	58	29	48	102	Bacteria	Acidobacteriota	Acidobacteriae	unclassified_Bacteria	unclassified_Bacteria	Subgroup 22			
0	0	0	23	0	0	0	0	0	0	0	0	Bacteria	Acidobacteriota	Thermoanaerobaculia	Thermoanaerobaculales	Thermoanaerobaculaceae	Subgroup 23			
0	31	0	0	39	0	0	0	0	0	0	0	Bacteria	Acidobacteriota	Thermoanaerobaculia	Thermoanaerobaculales	Thermoanaerobaculaceae	Subgroup 23			
0	0	0	0	23	0	0	0	0	0	0	0	Bacteria	Acidobacteriota	Thermoanaerobaculia	Thermoanaerobaculales	Thermoanaerobaculaceae	Subgroup 23			
0	0	0	0	0	0	56	0	0	0	0	0	Bacteria	Acidobacteriota	Holophagae	unclassified_Bacteria	unclassified_Bacteria	Subgroup 7			
0	0	0	0	0	0	11	0	0	0	0	0	Bacteria	Acidobacteriota	unclassified_Bacteria	unclassified_Bacteria	unclassified_Bacteria				
0	0	0	0	0	0	9	0	0	0	0	0	Bacteria	Actinobacteriota	Thermoleophilia	Gaiellales	Gaiellaceae	Gaiella	Gaiella	sp.	EBR4-RS1
0	0	0	0	0	0	0	12	0	0	0	0	Bacteria	Actinobacteriota	Acidimicrobiia	Microtrichales	Ilumatobacteraceae	Ilumatobacter	Ilumatobacter	sp.	
0	0	8	0	0	0	0	0	0	0	0	0	Bacteria	Actinobacteriota	Acidimicrobiia	Microtrichales	Microtrichaceae	IMCC26207			
0	0	0	0	0	0	7	0	0	0	0	0	Bacteria	Actinobacteriota	Rubrobacteria	Rubrobacterales	Rubrobacteriaceae	Rubrobacter	Rubrobacter	sp.	
0	0	11	0	0	0	0	0	0	0	0	0	Bacteria	Actinobacteriota	Acidimicrobiia	Microtrichales	Microtrichaceae	Sva0996 marine group			
15	0	16	0	48	0	0	34	34	0	0	0	Bacteria	Actinobacteriota	Acidimicrobiia	Actinomarinales	unclassified_Bacteria				
0	17	0	0	0	0	0	0	0	0	0	0	Bacteria	Actinobacteriota	Acidimicrobiia	Actinomarinales	unclassified_Bacteria				WH6-7
0	0	0	0	0	0	72	38	27	0	0	0	Bacteria	Actinobacteriota	Acidimicrobiia	Actinomarinales	unclassified_Bacteria				YC-ZSS-LKJ173
0	0	0	0	0	0	88	31	47	0	0	0	Bacteria	Actinobacteriota	Acidimicrobiia	Actinomarinales	unclassified_Bacteria				
0	0	0	0	0	0	59	0	0	30	0	0	Bacteria	Actinobacteriota	Actinobacteria	Euzebyales	Euzebyaceae				
0	0	6	0	0	0	151	0	39	0	0	0	Bacteria	Actinobacteriota	Actinobacteria	Euzebyales	Euzebyaceae				
0	0	0	0	0	0	0	19	12	0	0	0	Bacteria	Actinobacteriota	Actinobacteria	Frankiales	Sporichthyaceae				
0	0	0	0	0	0	6	0	0	0	0	0	Bacteria	Actinobacteriota	Thermoleophilia	Gaiellales	unclassified_Bacteria				
0	0	0	0	0	0	64	0	0	0	0	0	Bacteria	Actinobacteriota	Thermoleophilia	Gaiellales	unclassified_Bacteria				
0	0	0	0	0	0	69	0	0	0	0	5	Bacteria	Actinobacteriota	Acidimicrobiia	Microtrichales	unclassified_Bacteria				
0	0	0	4	0	0	0	0	0	0	0	0	Bacteria	Actinobacteriota	Actinobacteria	Nitriliruptorales	Nitriliruptoraceae				
0	10	0	0	12	0	56	28	19	17	0	11	Bacteria	Actinobacteriota	Actinobacteria	Nitriliruptorales	Nitriliruptoraceae				
0	0	0	0	0	0	90	0	14	13	0	22	Bacteria	Actinobacteriota	Actinobacteria	Nitriliruptorales	Nitriliruptoraceae				
0	0	0	0	0	0	15	0	0	0	0	0	Bacteria	Actinobacteriota	Actinobacteria	Nitriliruptorales	Nitriliruptoraceae				YC-ZSS-LKJ90
0	30	16	0	0	0	0	0	0	0	0	0	Bacteria	Actinobacteriota	Actinobacteria	Propionibacteriales	Propionibacteriaceae				SOGA22_gram-positive
0	5	0	0	0	0	0	0	0	0	0	0	Bacteria	Actinobacteriota	Thermoleophilia	Solirubrobacterales	unclassified_Bacteria				67-14
0	0	0	0	0	0	10	0	0	0	0	0	Bacteria	Actinobacteriota	Acidimicrobiia	unclassified_Bacteria	unclassified_Bacteria				
0	0	0	0	0	0	0	12	0	0	36	15	Bacteria	Armatimonadota	Fimbriimonadia	Fimbriimonadales	unclassified_Bacteria				
0	0	0	9	0	0	0	0	0	0	0	0	Archaea	Asgardarchaeota	Lokiarchaeia	unclassified_Archaea	unclassified_Archaea				
0	0	0	0	0	0	5	0	0	0	0	0	Bacteria	Bacteroidota	Bacteroidia	Cytophagales	Cyclobacteriaceae	Algoriphagus	Algoriphagus	sp.	
0	0	111	0	17	68	64	0	51	147	61	49	Bacteria	Bacteroidota	Rhodothermia	Balneolales	Balneolaceae	Aliifodinibius	Aliifodinibius	sp.	
0	0	29	0	0	0	0	0	0	0	0	0	Bacteria	Bacteroidota	Rhodothermia	Balneolales	Balneolaceae	Aliifodinibius	Aliifodinibius	sp.	
0	0	94	37	42	40	0	0	0	0	0	0	Bacteria	Bacteroidota	Bacteroidia	Bacteroidales	Marinilabiliaceae	Anaerophaga	Anaerophaga	sp.	
0	0	0	0	0	25	0	0	0	0	0	0	Bacteria	Bacteroidota	Bacteroidia	Bacteroidales	Marinilabiliaceae	Anaerophaga	Anaerophaga	sp.	
0	0	0	91	0	0	0	0	0	0	0	0	Bacteria	Bacteroidota	Rhodothermia	Balneolales	Balneolaceae	B2706-C7			
41	0	0	33	0	0	0	0	0	0	0	0	Bacteria	Bacteroidota	Rhodothermia	Balneolales	Balneolaceae	Balneola	Balneola	sp.	
20	0	11	0	0	0	0	0	0	0	0	0	Bacteria	Bacteroidota	Rhodothermia	Balneolales	Balneolaceae	Balneolaceae			
0	0	0	0	0	0	0	49	29	48	46	0	Bacteria	Bacteroidota	Bacteroidia	Cytophagales	Cyclobacteriaceae	Catalinimonas	Catalinimonas	sp.	
0	0	0	0	0	0	0	0	8	22	0	0	Bacteria	Bacteroidota	Bacteroidia	Cytophagales	Cyclobacteriaceae	Cesiribacter	Cesiribacter	sp.	JJ021
0	0	0	29	0	0	0	0	0	0	0	0	Bacteria	Bacteroidota	Chlorobia	Chlorobiales	Chloroherpetonaceae	Chloroherpeton	Chloroherpeton	thalassium	ATCC-35110
0	14	62	0	0	0	0	0	0	0	0	0	Bacteria	Bacteroidota	Bacteroidia	Flavobacteriales	Crocinitomicaceae	Crocinitomix	Crocinitomix	sp.	
0	12	0	10	0	0	0	0	0	0	21	0	Bacteria	Bacteroidota	Bacteroidia	Flavobacteriales	Cryomorphaceae	Cryomorpha	Cryomorpha	sp.	
0	0	0	18	0	0	0	0	18	15	0	0	Bacteria	Bacteroidota	Bacteroidia	Flavobacteriales	Cryomorphaceae	Cryomorpha	Cryomorpha	sp.	
0	0	0	0	11	31	0	0	0	0	0	0	Bacteria	Bacteroidota	Bacteroidia	Sphingobacteriales	unclassified_Bacteria	E6aC02			
0	40	15	36	43	86	0	0	0	0	0	0	Bacteria	Bacteroidota	Bacteroidia	Sphingobacteriales	unclassified_Bacteria	E6aC02			
28	38	0	0	0	0	0	0	0	0	0	0	Bacteria	Bacteroidota	Bacteroidia	Cytophagales	Cyclobacteriaceae	Ekhidna	Ekhidna	sp.	Kor
0	0	0	70	0	0	0	0	0	0	0	0	Bacteria	Bacteroidota	Bacteroidia	Cytophagales	Cyclobacteriaceae	Ekhidna	Ekhidna	sp.	
0	0	0	52	0	0	0	0	0	0	0	0	Bacteria	Bacteroidota	Bacteroidia	Cytophagales	Cyclobacteriaceae	Ekhidna	Ekhidna	sp.	
12	0	0	49	0	0	0	0	0	0	0	0	Bacteria	Bacteroidota	Bacteroidia	Cytophagales	Flammeovirgaceae	Flexithrix	Flexithrix	sp.	
58	32	0	72	0	0	0	0	0	0	0	0	Bacteria	Bacteroidota	Bacteroidia	Cytophagales	Flammeovirgaceae	Flexithrix	Flexithrix	sp.	
5	0	0	35	0	0	0	0	0	0	0	0	Bacteria	Bacteroidota	Bacteroidia	Cytophagales	Flammeovirgaceae	Flexithrix	Flexithrix	sp.	
59	42	0	0	0	0	0	76	28	25	55	52	Bacteria	Bacteroidota	Bacteroidia	Cytophagales	Cyclobacteriaceae	Fulvivirga	Fulvivirga	sp.	
73	72	21	71	43	26	0	52	0	48	0	0	Bacteria	Bacteroidota	Bacteroidia	Cytophagales	Cyclobacteriaceae	Fulvivirga	Fulvivirga	sp.	
18	37	0	75	0	0	0	48	0	0	0	0	Bacteria	Bacteroidota	Rhodothermia	Balneolales	Balneolaceae	Gracilimonas	Gracilimonas	sp.	
26	57	56	0	0	62	0	86	0	49	0	31	Bacteria	Bacteroidota	Rhodothermia	Balneolales	Balneolaceae	Gracilimonas	Gracilimonas	sp.	
31	28	0	0	0	0	0	0	0	0	0	0	Bacteria	Bacteroidota	Rhodothermia	Balneolales	Balneolaceae	Gracilimonas	Gracilimonas	rosea	
0	0	0	0	0	0	0	0	4	0	0	0	Bacteria	Bacteroidota	Ignavibacteria	Ignavibacteriales	Ignavibacteriaceae	Ignavibacterium	Ignavibacterium	sp.	
0	0	0	0	19	0	0	0	0	32	35	0	Bacteria	Bacteroidota	Ignavibacteria	Ignavibacteriales	Ignavibacteriaceae	Ignavibacterium	Ignavibacterium	sp.	
0	0	0	0	0	0	0	0	0	15	0	0	Bacteria	Bacteroidota	Ignavibacteria	Ignavibacteriales	Melioribacteraceae	IheB3-7			
0	0	0	36	0	0	0	0	0	0	0	0	Bacteria	Bacteroidota	Ignavibacteria	Ignavibacteriales	Melioribacteraceae	IheB3-7			
0	0	0	0	0	62	0	0	0	0	0	0	Bacteria	Bacteroidota	Ignavibacteria	Ignavibacteriales	Melioribacteraceae	IheB3-7			
0	9	0	0	204	242	35	213	428	247	256	311	Bacteria	Bacteroidota	Bacteroidia	Cytophagales	Cyclobacteriaceae	Imperialibacter	Imperialibacter	sp.	
0	72	0	0	0	0	0	0	0	0	0	0	Bacteria	Bacteroidota	Bacteroidia	Chitinophagales	Saprospiraceae	Lewinella	Lewinella	sp.	
43	0	0	33	0	0	0	50	0	0	0	0	Bacteria	Bacteroidota	Bacteroidia	Chitinophagales	Saprospiraceae	Lewinella	Lewinella	sp.	
31	24	0	13	0	0	0	0	0	0	0	0	Bacteria	Bacteroidota	Bacteroidia	Chitinophagales	Saprospiraceae	Lewinella	Cladosiphon	okamuranus	
0	43	0	27	0	0	0	0	0	0	6	0	Bacteria	Bacteroidota	Bacteroidia	Chitinophagales	Saprospiraceae	Lewinella	Lewinella	sp.	
61	26	0	59	0	0	0	0	0	0	0	0	Bacteria	Bacteroidota	Bacteroidia	Chitinophagales	Saprospiraceae	Lewinella	Lewinella	sp.	
0	28	0	74	50	0	0	0	0	0	0	0	Bacteria	Bacteroidota	Bacteroidia	Chitinophagales	Saprospiraceae	Lewinella	Lewinella	sp.	
0	0	0	0	10	17	0	37	32	0	0	0	Bacteria	Bacteroidota	Bacteroidia	Cytophagales	Flammeovirgaceae	Luteivirga	Luteivirga	sp.	
0	0	0	36	0	0	0	0	0	0	0	0	Bacteria	Bacteroidota	Bacteroidia	Cytophagales	Cyclobacteriaceae	Marinoscillum	Marinoscillum	sp.	
125	98	0	193	0	23	0	43	0	0	0	0	Bacteria	Bacteroidota	Bacteroidia	Cytophagales	Cyclobacteriaceae	Marinoscillum	Marinoscillum	sp.	
36	30	0	0	0	0	0	0	0	0	0	0	Bacteria	Bacteroidota	Bacteroidia	Cytophagales	Cyclobacteriaceae	Marivirga	Marivirga	sp.	
0	0	133	0	0	42	0	0	0	0	0	0	Bacteria	Bacteroidota	Bacteroidia	Cytophagales	Cyclobacteriaceae	Marivirga	Marivirga	sp.	
0	0	0	0	0	0	0	0	59	0	22	53	Bacteria	Bacteroidota	Rhodothermia	Balneolales	Balneolaceae	MAT-CR-P4-C12			
0	0	0	0	0	0	0	47	0	30	0	0	Bacteria	Bacteroidota	Rhodothermia	Balneolales	Balneolaceae	MAT-CR-P4-C12			
0	0	0	0	0	0	0	0	25	0	0	11	Bacteria	Bacteroidota	Rhodothermia	Balneolales	Balneolaceae	MAT-CR-P4-C12			
0	0	20	0	46	44	0	0	21	49	29	37	Bacteria	Bacteroidota	Rhodothermia	Balneolales	Balneolaceae	MAT-CR-P4-C12			
0	56	0	9	24	63	0	0	22	0	20	22	Bacteria	Bacteroidota	Ignavibacteria	Ignavibacteriales	Melioribacteraceae	Melioribacter	Melioribacter	sp.	
0	0	0	7	0	0	0	0	0	0	0	0	Bacteria	Bacteroidota	Rhodothermia	Balneolales	Balneolaceae	ML310M-34			
0	0	23	0	0	0	0	0	0	0	0	0	Bacteria	Bacteroidota	Bacteroidia	unclassified_Bacteria	unclassified_Bacteria	ML602M-17			
0	0	0	20	0	0	0	0	0	0	0	0	Bacteria	Bacteroidota	Bacteroidia	unclassified_Bacteria	unclassified_Bacteria	ML602M-17			
0	0	0	11	0	0	0	0	0	0	0	0	Bacteria	Bacteroidota	Bacteroidia	unclassified_Bacteria	unclassified_Bacteria	ML602M-17			
0	0	0	0	0	0	0	0	14	0	0	35	Bacteria	Bacteroidota	Bacteroidia	Bacteroidales	unclassified_Bacteria	ML635J-40 aquatic group			
0	0	0	11	0	0	0	0	0	7	16	0	Bacteria	Bacteroidota	Bacteroidia	Bacteroidales	unclassified_Bacteria	ML635J-40 aquatic group			
0	0	0	0	0	0	0	0	51	47	96	15	Bacteria	Bacteroidota	Bacteroidia	Cytophagales	unclassified_Bacteria	MWH-CFBk5			
0	0	0	79	0	0	0	0	0	0	0	0	Bacteria	Bacteroidota	Rhodothermia	Rhodothermales	Rhodothermaceae	Natronotalea	Natronotalea	proteilytica	
0	0	0	0	0	0	0	0	0	0	7	0	Bacteria	Bacteroidota	Bacteroidia	Flavobacteriales	Flavobacteriaceae	Nonlabens	Nonlabens	sp.	
0	0	38	0	0	0	0	33	22	38	50	0	Bacteria	Bacteroidota	Bacteroidia	Flavobacteriales	Cryomorphaceae	Owenweeksia	Owenweeksia	sp.	
0	0	0	14	0	0	0	0	0	0	0	0	Bacteria	Bacteroidota	Bacteroidia	Flavobacteriales	Cryomorphaceae	Owenweeksia	Owenweeksia	sp.	
0	0	0	0	30	36	118	56	22	29	0	152	Bacteria	Bacteroidota	Bacteroidia	Chitinophagales	Saprospiraceae	Phaeodactylibacter	Phaeodactylibacter	sp.	
0	31	0	29	94	0	0	0	0	0	0	53	Bacteria	Bacteroidota	Bacteroidia	Chitinophagales	Saprospiraceae	Phaeodactylibacter	Phaeodactylibacter	sp.	
54	55	0	48	0	0	0	0	0	0	0	0	Bacteria	Bacteroidota	Bacteroidia	Chitinophagales	Saprospiraceae	Phaeodactylibacter	Phaeodactylibacter	xiamenensis	
8	56	0	0	0	27	0	37	0	0	0	0	Bacteria	Bacteroidota	Bacteroidia	Chitinophagales	Saprospiraceae	Phaeodactylibacter	Phaeodactylibacter	sp.	
34	21	0	0	0	0	0	0	0	0	0	0	Bacteria	Bacteroidota	Bacteroidia	Chitinophagales	Saprospiraceae	Phaeodactylibacter	Phaeodactylibacter	sp.	
0	8	0	0	62	14	7	0	0	0	0	75	Bacteria	Bacteroidota	Bacteroidia	Chitinophagales	Saprospiraceae	Phaeodactylibacter	Phaeodactylibacter	sp.	
55	80	0	136	51	38	24	0	67	54	0	0	Bacteria	Bacteroidota	Bacteroidia	Chitinophagales	Saprospiraceae	Phaeodactylibacter	Phaeodactylibacter	sp.	
0	0	0	67	0	0	0	0	0	0	0	0	Bacteria	Bacteroidota	Bacteroidia	Flavobacteriales	Flavobacteriaceae	Psychroflexus	Psychroflexus	sp.	
0	17	0	60	0	0	0	0	0	0	0	0	Bacteria	Bacteroidota	Bacteroidia	Flavobacteriales	Flavobacteriaceae	Psychroflexus	Psychroflexus	sp.	
0	0	0	95	0	0	0	0	0	0	0	0	Bacteria	Bacteroidota	Bacteroidia	Flavobacteriales	Flavobacteriaceae	Psychroflexus	Psychroflexus	sp.	
0	0	0	59	0	0	0	0	0	0	0	0	Bacteria	Bacteroidota	Bacteroidia	Cytophagales	Flammeovirgaceae	Rapidithrix	Rapidithrix	sp.	
0	0	0	0	0	0	0	49	0	0	0	0	Bacteria	Bacteroidota	Rhodothermia	Balneolales	Balneolaceae	Rhodohalobacter	Rhodohalobacter	sp.	
0	0	0	0	0	0	0	0	14	0	0	0	Bacteria	Bacteroidota	Bacteroidia	Flavobacteriales	Flavobacteriaceae	Robiginitalea	Robiginitalea	sp.	
0	9	0	0	0	0	0	0	0	0	0	0	Bacteria	Bacteroidota	Bacteroidia	Cytophagales	Cyclobacteriaceae	Roseivirga	Roseivirga	sp.	
0	0	0	20	0	0	0	0	0	0	0	0	Bacteria	Bacteroidota	Bacteroidia	Sphingobacteriales	unclassified_Bacteria	S15A-MN91			
0	0	41	0	0	0	0	0	0	0	0	0	Bacteria	Bacteroidota	Bacteroidia	Flavobacteriales	Flavobacteriaceae	Salegentibacter	Salegentibacter	sp.	
0	0	0	0	58	42	0	0	0	0	0	0	Bacteria	Bacteroidota	Rhodothermia	Rhodothermales	Rhodothermaceae	Salinibacter	Salinibacter	sp.	
0	0	0	0	41	81	89	327	302	82	272	228	Bacteria	Bacteroidota	Rhodothermia	Rhodothermales	Rhodothermaceae	Salinibacter	Salinibacter	sp.	
0	0	0	0	0	0	0	0	18	0	28	0	Bacteria	Bacteroidota	Rhodothermia	Rhodothermales	Rhodothermaceae	Salinibacter	Salinibacter	iranicus	
0	0	0	0	0	0	0	55	28	0	0	0	Bacteria	Bacteroidota	Rhodothermia	Rhodothermales	Rhodothermaceae	Salinibacter	Salinibacter	sp.	
0	0	0	95	55	105	124	224	114	142	137	61	Bacteria	Bacteroidota	Rhodothermia	Rhodothermales	Rhodothermaceae	Salinibacter	Salinibacter	sp.	
0	0	0	27	0	0	0	0	0	0	0	0	Bacteria	Bacteroidota	Bacteroidia	Bacteroidales	Salinivirgaceae	Salinivirga	Salinivirga	sp.	
29	0	0	0	0	0	0	0	0	0	0	0	Bacteria	Bacteroidota	Rhodothermia	Rhodothermales	Rhodothermaceae	Salisaeta	Salisaeta	sp.	
0	0	0	0	0	47	0	0	90	0	33	0	Bacteria	Bacteroidota	Rhodothermia	Rhodothermales	Rhodothermaceae	Salisaeta	Salisaeta	sp.	
0	0	0	0	0	47	0	46	53	0	0	0	Bacteria	Bacteroidota	Rhodothermia	Rhodothermales	Rhodothermaceae	Salisaeta	Salisaeta	sp.	
0	108	0	1521	100	268	0	53	108	0	68	36	Bacteria	Bacteroidota	Rhodothermia	Rhodothermales	Rhodothermaceae	Salisaeta	Salisaeta	sp.	
12	7	0	8	0	0	0	0	0	0	0	0	Bacteria	Bacteroidota	Bacteroidia	Chitinophagales	Saprospiraceae	Saprospira	Saprospira	sp.	
0	0	0	23	52	102	0	0	37	87	0	85	Bacteria	Bacteroidota	Bacteroidia	Bacteroidales	unclassified_Bacteria	SB-5			
0	0	0	64	92	106	0	0	63	65	52	115	Bacteria	Bacteroidota	Bacteroidia	Bacteroidales	unclassified_Bacteria	SB-5			
0	0	0	13	0	0	0	0	0	0	0	0	Bacteria	Bacteroidota	Bacteroidia	unclassified_Bacteria	unclassified_Bacteria	SM1A07			
0	0	0	11	0	0	0	0	0	0	0	0	Bacteria	Bacteroidota	Bacteroidia	unclassified_Bacteria	unclassified_Bacteria	SM1A07			
0	0	0	0	0	8	0	14	0	0	0	0	Bacteria	Bacteroidota	Bacteroidia	unclassified_Bacteria	unclassified_Bacteria	SM1A07			
0	0	0	36	0	0	0	0	0	0	0	0	Bacteria	Bacteroidota	Bacteroidia	unclassified_Bacteria	unclassified_Bacteria	SM1A07			
10	0	0	32	0	0	0	0	0	0	0	0	Bacteria	Bacteroidota	Rhodothermia	Balneolales	Balneolaceae	Soortia	Soortia	sp.	
0	0	0	0	20	19	0	0	0	0	0	0	Bacteria	Bacteroidota	Bacteroidia	Sphingobacteriales	unclassified_Bacteria	ST-12K33			
0	0	0	0	0	47	0	0	0	0	0	0	Bacteria	Bacteroidota	Bacteroidia	Sphingobacteriales	unclassified_Bacteria	ST-12K33			
0	0	0	0	31	87	0	0	0	40	0	0	Bacteria	Bacteroidota	Bacteroidia	Sphingobacteriales	unclassified_Bacteria	ST-12K33			
0	0	0	0	0	0	0	0	0	45	0	0	Bacteria	Bacteroidota	Bacteroidia	Bacteroidales	Prolixibacteraceae	Tangfeifania	Tangfeifania	sp.	
0	83	19	0	67	368	0	0	0	43	0	31	Bacteria	Bacteroidota	Bacteroidia	Bacteroidales	Prolixibacteraceae	Tangfeifania	Tangfeifania	sp.	
0	0	0	0	0	0	12	0	0	0	0	0	Bacteria	Bacteroidota	Bacteroidia	Cytophagales	Cyclobacteriaceae	Tunicatimonas	Tunicatimonas	sp.	
0	40	0	0	0	0	0	0	0	0	0	0	Bacteria	Bacteroidota	Bacteroidia	Flavobacteriales	Cryomorphaceae	Vicingus	Vicingus	sp.	
36	38	12	0	0	0	0	20	0	0	0	0	Bacteria	Bacteroidota	Bacteroidia	Flavobacteriales	Cryomorphaceae	Vicingus	Vicingus	sp.	

0	0	0	0	0	26	0	0	0	0	0	56	Bacteria	Bacteroidota	Bacteroidia	Bacteroidales	Bacteroidetes BD2-2				
0	39	0	34	43	89	31	0	0	60	18	53	Bacteria	Bacteroidota	Bacteroidia	Bacteroidales	Bacteroidetes BD2-2				
0	0	0	47	0	0	0	0	0	0	0	0	Bacteria	Bacteroidota	Bacteroidia	Bacteroidales	Bacteroidetes BD2-2				
0	0	0	37	0	45	0	0	0	0	0	0	Bacteria	Bacteroidota	Bacteroidia	Bacteroidales	Bacteroidetes BD2-2				
0	0	0	21	0	0	0	0	27	0	0	0	Bacteria	Bacteroidota	Bacteroidia	Bacteroidales	Bacteroidetes BD2-2				
0	0	0	31	0	0	0	0	0	0	0	0	Bacteria	Bacteroidota	Bacteroidia	Bacteroidales	Bacteroidetes BD2-2				
0	0	0	82	0	0	0	0	0	0	0	0	Bacteria	Bacteroidota	Bacteroidia	Bacteroidales	Bacteroidetes BD2-2				
0	34	0	0	19	61	0	0	0	31	0	0	Bacteria	Bacteroidota	Bacteroidia	Bacteroidales	Marinilabiliaceae				
0	0	0	0	0	19	0	0	0	0	0	0	Bacteria	Bacteroidota	Bacteroidia	Bacteroidales	Marinilabiliaceae				
0	0	0	0	0	28	0	0	0	0	0	0	Bacteria	Bacteroidota	Bacteroidia	Bacteroidales	Marinilabiliaceae				YC-LK-LKJ5
0	0	0	3	0	0	0	0	0	0	0	0	Bacteria	Bacteroidota	Bacteroidia	Bacteroidales	Marinilabiliaceae				
0	0	0	0	0	0	0	0	0	0	16	0	Bacteria	Bacteroidota	Bacteroidia	Bacteroidales	Marinilabiliaceae				
0	0	0	57	54	70	0	0	0	0	0	0	Bacteria	Bacteroidota	Bacteroidia	Bacteroidales	Marinilabiliaceae				
0	56	0	27	0	19	0	0	0	0	0	0	Bacteria	Bacteroidota	Bacteroidia	Bacteroidales	Prolixibacteraceae				
0	0	0	0	0	0	0	0	38	0	0	38	Bacteria	Bacteroidota	Bacteroidia	Bacteroidales	Prolixibacteraceae				YC-ZSS-LKJ26
88	62	0	0	0	92	0	0	65	60	0	66	Bacteria	Bacteroidota	Bacteroidia	Bacteroidales	Prolixibacteraceae				
0	0	0	0	21	42	0	0	0	0	0	0	Bacteria	Bacteroidota	Bacteroidia	Bacteroidales	Salinivirgaceae				
0	0	0	19	0	0	0	0	0	0	0	0	Bacteria	Bacteroidota	Bacteroidia	Bacteroidales	Salinivirgaceae				
0	0	0	49	0	0	0	0	0	0	0	0	Bacteria	Bacteroidota	Bacteroidia	Bacteroidales	Salinivirgaceae				
0	0	0	50	0	0	0	0	0	3	0	0	Bacteria	Bacteroidota	Bacteroidia	Bacteroidales	Salinivirgaceae				
0	0	0	0	70	61	0	0	73	66	40	28	Bacteria	Bacteroidota	Bacteroidia	Bacteroidales	unclassified_Bacteria				
0	31	32	190	144	273	0	138	253	304	276	245	Bacteria	Bacteroidota	Bacteroidia	Bacteroidales	unclassified_Bacteria				
0	0	0	30	0	0	0	0	0	0	0	0	Bacteria	Bacteroidota	Bacteroidia	Bacteroidetes VC 2.1 Bac22	unclassified_Bacteria				
0	0	45	0	28	85	0	151	55	110	53	69	Bacteria	Bacteroidota	Rhodothermia	Balneolales	Balneolaceae				
0	0	0	0	0	19	0	0	0	0	0	0	Bacteria	Bacteroidota	Rhodothermia	Balneolales	Balneolaceae				
279	317	115	363	200	281	267	718	917	361	385	1032	Bacteria	Bacteroidota	Rhodothermia	Balneolales	Balneolaceae				
0	0	58	0	20	0	16	137	124	0	52	26	Bacteria	Bacteroidota	Rhodothermia	Balneolales	Balneolaceae				
0	0	33	0	0	0	38	0	0	80	0	47	Bacteria	Bacteroidota	Rhodothermia	Balneolales	Balneolaceae				
0	36	0	0	43	49	0	114	48	53	75	73	Bacteria	Bacteroidota	Rhodothermia	Balneolales	Balneolaceae				
50	120	9	104	195	48	2	177	69	0	31	27	Bacteria	Bacteroidota	Bacteroidia	Chitinophagales	Saprospiraceae				
129	111	0	53	49	101	0	90	21	0	25	37	Bacteria	Bacteroidota	Bacteroidia	Chitinophagales	Saprospiraceae				
0	66	0	42	0	0	0	0	0	0	0	0	Bacteria	Bacteroidota	Bacteroidia	Chitinophagales	Saprospiraceae				
0	0	0	0	0	0	0	133	55	30	0	73	Bacteria	Bacteroidota	Bacteroidia	Chitinophagales	Saprospiraceae				
54	35	0	52	0	0	0	23	28	0	0	0	Bacteria	Bacteroidota	Bacteroidia	Chitinophagales	Saprospiraceae				
91	118	0	180	19	14	0	54	35	35	61	14	Bacteria	Bacteroidota	Bacteroidia	Chitinophagales	Saprospiraceae				
0	0	0	0	0	0	0	0	0	0	14	0	Bacteria	Bacteroidota	Bacteroidia	Chitinophagales	unclassified_Bacteria				
0	0	0	21	0	0	0	0	0	0	0	0	Bacteria	Bacteroidota	Bacteroidia	Chitinophagales	unclassified_Bacteria				
0	16	0	0	0	0	0	0	0	0	0	0	Bacteria	Bacteroidota	Bacteroidia	Chitinophagales	unclassified_Bacteria				
0	0	0	0	25	0	15	49	109	11	6	26	Bacteria	Bacteroidota	Bacteroidia	Chitinophagales	unclassified_Bacteria				
35	54	0	0	0	0	0	0	0	15	0	0	Bacteria	Bacteroidota	Bacteroidia	Chitinophagales	unclassified_Bacteria				
177	19	0	163	19	0	0	0	0	0	0	0	Bacteria	Bacteroidota	Bacteroidia	Chitinophagales	unclassified_Bacteria				
11	0	0	0	0	0	0	0	0	0	0	0	Bacteria	Bacteroidota	Bacteroidia	Cytophagales	Amoebophilaceae				
15	13	0	0	0	0	0	0	0	0	0	0	Bacteria	Bacteroidota	Bacteroidia	Cytophagales	Amoebophilaceae				
8	0	0	0	0	0	0	0	0	0	0	0	Bacteria	Bacteroidota	Bacteroidia	Cytophagales	Amoebophilaceae				
0	30	0	58	0	0	0	0	0	0	0	0	Bacteria	Bacteroidota	Bacteroidia	Cytophagales	Amoebophilaceae				
0	0	0	22	0	0	0	0	0	0	0	0	Bacteria	Bacteroidota	Bacteroidia	Cytophagales	Bernardetiaceae				
15	16	9	86	0	0	12	0	37	56	42	0	Bacteria	Bacteroidota	Bacteroidia	Cytophagales	Cyclobacteriaceae				
236	17	0	151	62	136	35	108	194	0	136	130	Bacteria	Bacteroidota	Bacteroidia	Cytophagales	Cyclobacteriaceae				
0	4	0	82	11	0	0	0	0	0	0	0	Bacteria	Bacteroidota	Bacteroidia	Cytophagales	Cyclobacteriaceae				
0	0	0	0	27	0	0	0	0	0	0	33	Bacteria	Bacteroidota	Bacteroidia	Cytophagales	Cyclobacteriaceae				
0	0	0	0	23	0	0	0	0	0	0	0	Bacteria	Bacteroidota	Bacteroidia	Cytophagales	Cyclobacteriaceae				
34	14	0	0	0	0	0	101	0	85	11	0	Bacteria	Bacteroidota	Bacteroidia	Cytophagales	Cyclobacteriaceae				
0	56	0	0	49	0	0	0	0	0	0	0	Bacteria	Bacteroidota	Bacteroidia	Cytophagales	Cyclobacteriaceae				
0	0	24	0	27	29	0	0	33	6	0	0	Bacteria	Bacteroidota	Bacteroidia	Cytophagales	Cyclobacteriaceae				
0	0	0	7	0	0	0	0	0	0	0	0	Bacteria	Bacteroidota	Bacteroidia	Cytophagales	Cyclobacteriaceae				
0	0	0	8	0	0	0	0	0	0	0	0	Bacteria	Bacteroidota	Bacteroidia	Cytophagales	Flammeovirgaceae				
0	0	0	0	0	0	0	21	0	0	0	0	Bacteria	Bacteroidota	Bacteroidia	Cytophagales	Flammeovirgaceae				
17	0	0	60	0	0	0	23	10	0	0	0	Bacteria	Bacteroidota	Bacteroidia	Cytophagales	unclassified_Bacteria				
0	0	4	0	0	0	0	0	0	0	0	0	Bacteria	Bacteroidota	Bacteroidia	Flavobacteriales	Crocinitomicaceae				
0	16	0	0	71	0	0	0	0	0	0	0	Bacteria	Bacteroidota	Bacteroidia	Flavobacteriales	Crocinitomicaceae				
0	36	0	0	0	0	0	0	0	0	0	0	Bacteria	Bacteroidota	Bacteroidia	Flavobacteriales	Crocinitomicaceae				
29	26	0	51	0	0	0	0	11	0	0	0	Bacteria	Bacteroidota	Bacteroidia	Flavobacteriales	Cryomorphaceae				
0	22	0	0	0	0	0	0	0	0	0	0	Bacteria	Bacteroidota	Bacteroidia	Flavobacteriales	Cryomorphaceae				
20	0	0	0	0	0	0	0	0	0	0	0	Bacteria	Bacteroidota	Bacteroidia	Flavobacteriales	Cryomorphaceae				
31	0	0	0	0	0	0	0	0	0	0	0	Bacteria	Bacteroidota	Bacteroidia	Flavobacteriales	Cryomorphaceae				
0	0	0	0	0	0	0	21	0	0	0	0	Bacteria	Bacteroidota	Bacteroidia	Flavobacteriales	Cryomorphaceae				
16	0	0	0	0	0	0	0	0	0	0	0	Bacteria	Bacteroidota	Bacteroidia	Flavobacteriales	Flavobacteriaceae				
36	39	0	0	0	0	0	0	0	0	0	0	Bacteria	Bacteroidota	Bacteroidia	Flavobacteriales	Flavobacteriaceae				
0	0	0	14	0	0	0	0	0	14	0	0	Bacteria	Bacteroidota	Bacteroidia	Flavobacteriales	Flavobacteriaceae				
0	0	0	10	0	0	0	0	0	0	0	0	Bacteria	Bacteroidota	Bacteroidia	Flavobacteriales	Flavobacteriaceae				
0	0	18	0	0	0	0	0	0	15	0	0	Bacteria	Bacteroidota	Bacteroidia	Flavobacteriales	unclassified_Bacteria				
0	0	0	0	0	10	0	0	0	0	0	0	Bacteria	Bacteroidota	Ignavibacteria	Ignavibacteriales	unclassified_Bacteria				
12	0	0	0	0	0	0	0	0	0	0	0	Bacteria	Bacteroidota	Kapabacteria	Kapabacteriales	unclassified_Bacteria				
0	0	0	0	0	14	0	0	0	0	0	0	Bacteria	Bacteroidota	Kapabacteria	Kapabacteriales	unclassified_Bacteria				
0	0	0	0	0	0	0	17	0	0	0	0	Bacteria	Bacteroidota	Kapabacteria	Kapabacteriales	unclassified_Bacteria				
0	0	0	0	8	0	0	0	0	0	0	0	Bacteria	Bacteroidota	Kapabacteria	Kapabacteriales	unclassified_Bacteria				
0	0	0	87	104	66	0	0	22	61	0	0	Bacteria	Bacteroidota	Kapabacteria	Kapabacteriales	unclassified_Bacteria				
19	90	11	0	27	42	33	106	0	72	89	10	Bacteria	Bacteroidota	Rhodothermia	Rhodothermales	Rhodothermaceae				
28	22	0	0	0	0	0	0	0	0	0	0	Bacteria	Bacteroidota	Rhodothermia	Rhodothermales	Rhodothermaceae				
0	0	19	0	0	0	0	0	0	0	0	0	Bacteria	Bacteroidota	Rhodothermia	Rhodothermales	Rhodothermaceae				
0	0	0	0	0	0	0	0	0	18	0	0	Bacteria	Bacteroidota	Rhodothermia	Rhodothermales	Rhodothermaceae				
0	0	0	0	53	23	28	25	0	0	0	17	Bacteria	Bacteroidota	Rhodothermia	Rhodothermales	Rhodothermaceae				
0	0	0	0	37	46	398	77	93	0	5	97	Bacteria	Bacteroidota	Rhodothermia	Rhodothermales	Rhodothermaceae				
0	0	0	0	0	0	0	71	19	0	0	0	Bacteria	Bacteroidota	Rhodothermia	Rhodothermales	Rhodothermaceae				
0	0	0	0	0	0	45	42	81	0	0	0	Bacteria	Bacteroidota	Rhodothermia	Rhodothermales	Rhodothermaceae				
0	0	0	0	0	0	0	0	0	0	0	57	Bacteria	Bacteroidota	Rhodothermia	Rhodothermales	Rhodothermaceae				
0	0	0	0	0	0	0	27	17	54	27	51	Bacteria	Bacteroidota	Rhodothermia	Rhodothermales	Rhodothermaceae				
0	0	0	0	113	0	114	54	154	21	62	60	Bacteria	Bacteroidota	Rhodothermia	Rhodothermales	Rhodothermaceae				
0	0	0	0	17	0	0	0	0	0	10	0	Bacteria	Bacteroidota	Bacteroidia	Sphingobacteriales	Lentimicrobiaceae				
0	10	0	0	82	99	0	0	44	71	26	64	Bacteria	Bacteroidota	Bacteroidia	Sphingobacteriales	Lentimicrobiaceae				
0	11	0	0	0	0	0	0	0	0	0	0	Bacteria	Bacteroidota	Bacteroidia	Sphingobacteriales	Lentimicrobiaceae				
0	19	0	21	40	22	0	0	0	79	0	0	Bacteria	Bacteroidota	Bacteroidia	Sphingobacteriales	Lentimicrobiaceae				
0	0	0	0	0	36	0	0	0	0	0	0	Bacteria	Bacteroidota	Bacteroidia	Sphingobacteriales	unclassified_Bacteria				
19	20	2	99	20	21	0	54	30	0	7	17	Bacteria	Bacteroidota	Bacteroidia	unclassified_Bacteria	unclassified_Bacteria				
0	0	0	0	0	0	0	16	0	0	0	0	Bacteria	Bdellovibrionota	Oligoflexia	unclassified_Bacteria	unclassified_Bacteria	0319-6G20			
0	0	0	0	14	0	0	0	0	16	18	0	Bacteria	Bdellovibrionota	Oligoflexia	unclassified_Bacteria	unclassified_Bacteria	0319-6G20			
0	11	0	45	0	0	0	0	0	0	0	0	Bacteria	Bdellovibrionota	Oligoflexia	unclassified_Bacteria	unclassified_Bacteria	0319-6G20			
0	0	0	0	0	0	0	0	0	32	16	0	Bacteria	Bdellovibrionota	Oligoflexia	unclassified_Bacteria	unclassified_Bacteria	0319-6G20			
0	0	0	0	24	0	8	0	28	3	13	31	Bacteria	Bdellovibrionota	Bdellovibrionia	Bdellovibrionales	Bdellovibrionaceae	Bdellovibrio	Bdellovibrio	sp.	
0	0	0	0	0	0	0	0	14	0	0	49	Bacteria	Bdellovibrionota	Bdellovibrionia	Bdellovibrionales	Bdellovibrionaceae	Bdellovibrio	Bdellovibrio	sp.	
0	0	0	0	0	0	0	0	0	17	0	8	Bacteria	Bdellovibrionota	Bdellovibrionia	Bdellovibrionales	Bdellovibrionaceae	Bdellovibrio	Bdellovibrio	sp.	
20	0	0	0	0	0	0	0	0	0	0	0	Bacteria	Bdellovibrionota	Bdellovibrionia	Bdellovibrionales	Bdellovibrionaceae	Bdellovibrio	Bdellovibrio	sp.	
15	0	0	0	0	0	0	0	0	0	0	0	Bacteria	Bdellovibrionota	Bdellovibrionia	Bacteriovoracales	Bacteriovoracaceae	Halobacteriovorax	Halobacteriovorax	sp.	
0	0	0	12	0	0	0	0	16	0	0	0	Bacteria	Bdellovibrionota	Bdellovibrionia	Bdellovibrionales	Bdellovibrionaceae	OM27 clade			
46	0	0	0	0	0	0	0	14	5	0	0	Bacteria	Bdellovibrionota	Bdellovibrionia	Bdellovibrionales	Bdellovibrionaceae	OM27 clade			
0	0	0	0	0	0	0	11	0	0	0	0	Bacteria	Bdellovibrionota	Bdellovibrionia	Bdellovibrionales	Bdellovibrionaceae	OM27 clade			
0	0	0	0	0	0	0	0	0	0	20	27	Bacteria	Bdellovibrionota	Bdellovibrionia	Bacteriovoracales	Bacteriovoracaceae	Peredibacter	Peredibacter	sp.	
39	0	0	0	0	0	0	0	5	0	0	0	Bacteria	Bdellovibrionota	Bdellovibrionia	Bacteriovoracales	Bacteriovoracaceae	Peredibacter	Peredibacter	sp.	
0	25	0	0	0	0	0	0	0	0	0	0	Bacteria	Bdellovibrionota	Bdellovibrionia	Bacteriovoracales	Bacteriovoracaceae	Peredibacter	Peredibacter	sp.	
0	0	0	0	0	0	36	0	0	0	27	0	Bacteria	Bdellovibrionota	Bdellovibrionia	Bacteriovoracales	Bacteriovoracaceae	Peredibacter	Peredibacter	sp.	
11	0	0	0	0	0	0	0	0	0	0	0	Bacteria	Bdellovibrionota	Oligoflexia	Oligoflexales	Oligoflexales	Pseudobacteriovorax	Pseudobacteriovorax	antillogorgiicola	
14	0	12	120	17	0	0	0	0	0	0	0	Bacteria	Bdellovibrionota	Bdellovibrionia	Bacteriovoracales	Bacteriovoracaceae				
0	0	0	24	0	0	0	0	0	0	0	0	Bacteria	Bdellovibrionota	Bdellovibrionia	Bacteriovoracales	Bacteriovoracaceae				
0	0	0	35	0	0	0	0	0	0	0	0	Bacteria	Bdellovibrionota	Bdellovibrionia	Bacteriovoracales	Bacteriovoracaceae				
0	9	0	0	0	0	0	0	0	0	0	0	Bacteria	Bdellovibrionota	Oligoflexia	Oligoflexales	unclassified_Bacteria				
0	0	0	9	0	0	13	0	0	0	11	0	Bacteria	Bdellovibrionota	Oligoflexia	Oligoflexales	unclassified_Bacteria				
0	0	0	0	0	19	0	19	0	0	0	0	Bacteria	Bdellovibrionota	Oligoflexia	Oligoflexales	unclassified_Bacteria				
40	0	0	0	0	0	0	0	0	0	0	0	Bacteria	Bdellovibrionota	Oligoflexia	Oligoflexales	unclassified_Bacteria				
0	0	0	52	0	0	0	0	0	0	0	0	Bacteria	Bdellovibrionota	Oligoflexia	Silvanigrellales	Silvanigrellaceae				
0	0	0	12	0	43	0	0	0	67	12	31	Bacteria	Calditrichota	Calditrichia	Calditrichales	Calditrichaceae	Caldithrix	Caldithrix	sp.	
8	0	0	0	0	0	0	0	0	0	0	6	Bacteria	Calditrichota	Calditrichia	Calditrichales	Calditrichaceae	Caldithrix	Caldithrix	sp.	
0	13	0	0	0	0	0	0	0	0	0	0	Bacteria	Calditrichota	Calditrichia	Calditrichales	Calditrichaceae				
0	0	0	0	0	39	0	0	25	71	0	35	Bacteria	Calditrichota	Calditrichia	Calditrichales	Calditrichaceae				
0	0	0	24	0	0	0	0	0	0	0	0	Bacteria	Campilobacterota	Campylobacteria	Campylobacterales	Arcobacteraceae	Arcobacter	Arcobacter	sp.	
0	0	0	57	0	0	0	0	0	0	0	0	Bacteria	Campilobacterota	Campylobacteria	Campylobacterales	Arcobacteraceae	Candidatus Arcobacter	Candidatus Arcobacter	sulfidicus	
0	0	24	0	0	0	0	0	0	0	0	0	Bacteria	Campilobacterota	Campylobacteria	Campylobacterales	Arcobacteraceae	Malaciobacter	Malaciobacter	sp.	
0	0	0	25	0	0	0	0	0	0	0	0	Bacteria	Campilobacterota	Campylobacteria	Campylobacterales	unclassified_Bacteria	Rs-M59 termite group			
0	0	0	31	0	0	0	0	0	0	0	0	Bacteria	Campilobacterota	Campylobacteria	Campylobacterales	Sulfurospirillaceae	Sulfurospirillum	Sulfurospirillum	sp.	
0	0	0	16	0	0	0	0	0	0	0	0	Bacteria	Campilobacterota	Campylobacteria	Campylobacterales	Arcobacteraceae				
0	0	0	41	0	0	0	0	0	0	0	0	Bacteria	Campilobacterota	Campylobacteria	Campylobacterales	Helicobacteraceae				
0	0	0	103	0	0	0	0	0	7	0	0	Bacteria	Campilobacterota	Campylobacteria	Campylobacterales	Helicobacteraceae				
0	0	0	14	0	0	0	0	0	0	0	0	Bacteria	Campilobacterota	Campylobacteria	Campylobacterales	unclassified_Bacteria				
0	0	0	0	0	40	0	0	0	0	0	64	Bacteria	Chloroflexi	Anaerolineae	Anaerolineales	Anaerolineaceae	ADurb.Bin120			
0	0	0	0	0	0	0	0	0	61	0	0	Bacteria	Chloroflexi	Anaerolineae	Anaerolineales	Anaerolineaceae	ADurb.Bin120			
0	0	0	2	10	0	112	38	20	0	0	35	Bacteria	Chloroflexi	Chloroflexia	Thermomicrobiales	unclassified_Bacteria	AKYG1722			
0	0	0	0	0	0	126	0	16	0	0	0	Bacteria	Chloroflexi	Anaerolineae	Ardenticatenales	Ardenticatenaceae	Ardenticatena	Ardenticatena	sp.	
0	0	0	0	0	0	0	23	0	0	0	41	Bacteria	Chloroflexi	Anaerolineae	Ardenticatenales	Anaerolineaceae	Bellilinea	Bellilinea	sp.	
0	33	0	0	0	28	0	0	0	0	0	0	Bacteria	Chloroflexi	Chloroflexia	Chloroflexales	Chloroflexaceae	Candidatus Chlorothrix	Candidatus Chlorothrix	sp.	
0	5	0	14	28	29	0	22	93	0	0	60	Bacteria	Chloroflexi	Chloroflexia	Chloroflexales	Chloroflexaceae	Candidatus Chlorothrix	Candidatus Chlorothrix	sp.	
0	0	0	0	33	19	0	0	0	0	0	0	Bacteria	Chloroflexi	Chloroflexia	Chloroflexales	Chloroflexaceae	Candidatus Chlorothrix	Candidatus Chlorothrix	sp.	
0	0	0	0	0	0	0	77	61	15	49	0	Bacteria	Chloroflexi	Chloroflexia	Chloroflexales	Chloroflexaceae	Candidatus Chlorothrix	Candidatus Chlorothrix	sp.	
0	0	0	0	85	161	44	88	128	62	100	139	Bacteria	Chloroflexi	Chloroflexia	Chloroflexales	Chloroflexaceae	Candidatus Chlorothrix	Candidatus Chlorothrix	sp.	
0	0	0	0	55	54	0	68	84	0	48	84	Bacteria	Chloroflexi	Chloroflexia	Chloroflexales	Chloroflexaceae	Candidatus Chlorothrix	Candidatus Chlorothrix	sp.	
0	0	0	0	0	0	0	14	0	0	0	0	Bacteria	Chloroflexi	Chloroflexia	Chloroflexales	Chloroflexaceae	Oscillochloris	Oscillochloris	sp.	

0	0	0	0	0	0	0	15	0	0	0	0	Bacteria	Chloroflexi	Chloroflexia	Chloroflexales	Chloroflexaceae	Oscillochloris	Oscillochloris	sp.	
0	0	0	0	0	22	0	0	0	0	0	0	Bacteria	Chloroflexi	Anaerolineae	Anaerolineales	Anaerolineaceae	Pelolinea	Pelolinea	sp.	UCG-001
0	0	0	0	0	0	0	4	0	0	0	0	Bacteria	Chloroflexi	Dehalococcoidia	unclassified_Bacteria	unclassified_Bacteria	SAR202 clade			
0	0	0	0	0	0	0	0	13	0	0	0	Bacteria	Chloroflexi	Dehalococcoidia	unclassified_Bacteria	unclassified_Bacteria	SAR202 clade			
15	0	0	0	108	130	57	102	70	104	55	200	Bacteria	Chloroflexi	Anaerolineae	unclassified_Bacteria	unclassified_Bacteria	SBR1031			
335	88	0	204	107	315	0	540	237	164	138	238	Bacteria	Chloroflexi	Anaerolineae	unclassified_Bacteria	unclassified_Bacteria	SBR1031			A4b
0	0	0	0	61	57	0	40	0	50	28	0	Bacteria	Chloroflexi	Anaerolineae	unclassified_Bacteria	unclassified_Bacteria	SBR1031			A4b
308	4	0	65	45	32	0	293	171	86	118	109	Bacteria	Chloroflexi	Anaerolineae	unclassified_Bacteria	unclassified_Bacteria	SBR1031			A4b
0	0	0	0	0	0	0	40	24	0	0	0	Bacteria	Chloroflexi	Anaerolineae	unclassified_Bacteria	unclassified_Bacteria	SBR1031			A4b
157	67	0	460	642	729	60	805	837	647	912	778	Bacteria	Chloroflexi	Anaerolineae	unclassified_Bacteria	unclassified_Bacteria	SBR1031			A4b
161	38	0	0	308	286	307	197	303	327	370	480	Bacteria	Chloroflexi	Anaerolineae	unclassified_Bacteria	unclassified_Bacteria	SBR1031			
0	0	0	0	0	0	0	0	0	29	0	0	Bacteria	Chloroflexi	Anaerolineae	unclassified_Bacteria	unclassified_Bacteria	SBR1031			MO-CFX2
0	0	0	0	0	10	73	0	0	0	0	0	Bacteria	Chloroflexi	Anaerolineae	unclassified_Bacteria	unclassified_Bacteria	SBR1031			YC-ZSS-LKJ145
0	0	0	0	0	0	0	22	0	0	0	0	Bacteria	Chloroflexi	Anaerolineae	unclassified_Bacteria	unclassified_Bacteria	SBR1031			
0	0	0	0	0	0	155	25	40	0	22	49	Bacteria	Chloroflexi	Anaerolineae	unclassified_Bacteria	unclassified_Bacteria	SBR1031			
81	0	0	0	92	43	268	261	238	39	173	199	Bacteria	Chloroflexi	Anaerolineae	unclassified_Bacteria	unclassified_Bacteria	SBR1031			
0	0	0	0	110	77	69	0	0	82	77	55	Bacteria	Chloroflexi	Anaerolineae	unclassified_Bacteria	unclassified_Bacteria	SBR1031			SJA-101
0	54	0	20	368	217	49	0	0	97	0	75	Bacteria	Chloroflexi	Anaerolineae	unclassified_Bacteria	unclassified_Bacteria	SBR1031			
0	8	0	0	173	64	175	0	0	256	85	151	Bacteria	Chloroflexi	Anaerolineae	unclassified_Bacteria	unclassified_Bacteria	SBR1031			
0	0	0	0	0	0	16	0	0	0	0	0	Bacteria	Chloroflexi	Anaerolineae	unclassified_Bacteria	unclassified_Bacteria	SJA-15			
0	0	0	0	7	0	5	0	0	0	0	0	Bacteria	Chloroflexi	Anaerolineae	Anaerolineales	Anaerolineaceae				
0	0	0	0	0	30	0	0	0	0	0	0	Bacteria	Chloroflexi	Anaerolineae	Anaerolineales	Anaerolineaceae				
0	0	0	0	84	55	23	0	163	0	37	99	Bacteria	Chloroflexi	Anaerolineae	Anaerolineales	Anaerolineaceae				
0	0	0	18	0	12	0	0	0	0	0	0	Bacteria	Chloroflexi	Anaerolineae	Anaerolineales	Anaerolineaceae				
0	0	23	0	0	36	1950	53	30	0	63	68	Bacteria	Chloroflexi	Anaerolineae	Anaerolineales	Anaerolineaceae				
0	50	0	0	85	160	42	0	25	78	75	91	Bacteria	Chloroflexi	Anaerolineae	Anaerolineales	Anaerolineaceae				
0	24	0	0	48	18	190	40	34	14	23	113	Bacteria	Chloroflexi	Anaerolineae	Ardenticatenales	unclassified_Bacteria				
0	0	0	0	0	0	666	0	180	0	0	118	Bacteria	Chloroflexi	Anaerolineae	Ardenticatenales	unclassified_Bacteria				
0	0	0	0	0	0	0	0	20	0	6	0	Bacteria	Chloroflexi	Anaerolineae	Ardenticatenales	unclassified_Bacteria				
0	0	14	23	146	155	31	34	121	100	128	115	Bacteria	Chloroflexi	Anaerolineae	Ardenticatenales	unclassified_Bacteria				
0	0	0	0	47	0	0	28	0	0	0	0	Bacteria	Chloroflexi	Anaerolineae	Ardenticatenales	unclassified_Bacteria				
0	0	0	0	0	0	37	0	0	0	0	0	Bacteria	Chloroflexi	Anaerolineae	Caldilineales	Caldilineaceae				
0	0	0	0	0	0	47	0	0	0	0	0	Bacteria	Chloroflexi	Anaerolineae	Caldilineales	Caldilineaceae				
0	0	0	0	8	0	10	12	0	9	0	34	Bacteria	Chloroflexi	Anaerolineae	Caldilineales	Caldilineaceae				
0	0	0	0	24	0	0	0	0	0	0	0	Bacteria	Chloroflexi	Chloroflexia	Chloroflexales	Chloroflexaceae				
0	0	0	0	0	0	0	0	0	14	0	0	Bacteria	Chloroflexi	Anaerolineae	unclassified_Bacteria	unclassified_Bacteria				
0	11	0	0	0	0	0	0	0	0	6	0	Bacteria	Chloroflexi	KD4-96	unclassified_Bacteria	unclassified_Bacteria				
0	0	0	0	0	0	60	7	0	0	0	0	Bacteria	Chloroflexi	KD4-96	unclassified_Bacteria	unclassified_Bacteria				
0	0	0	0	0	0	15	0	0	0	0	0	Bacteria	Chloroflexi	OLB14	unclassified_Bacteria	unclassified_Bacteria				
0	0	0	19	0	0	0	0	0	0	0	0	Bacteria	Cloacimonadota	Cloacimonadia	Cloacimonadales	unclassified_Bacteria				
0	0	47	0	40	0	0	0	0	43	0	0	Bacteria	Cyanobacteria	Cyanophyceae	Oscillatoriales	Microcoleaceae	Arthrospira	Arthrospira	sp.	PCC-7345
0	0	0	58	0	0	0	0	0	0	0	0	Bacteria	Cyanobacteria	Cyanophyceae	Chroococcales	Chroococcaceae	Chroococcus	Chroococcus	sp.	HUW-799
0	0	72	0	300	314	0	256	189	158	309	119	Bacteria	Cyanobacteria	Cyanophyceae	Oscillatoriales	Coleofasciculaceae	Coleofasciculus	Coleofasciculus	sp.	PCC-7420
0	0	0	0	0	0	0	0	0	0	50	0	Bacteria	Cyanobacteria	Cyanophyceae	Oscillatoriales	Coleofasciculaceae	Coleofasciculus	Coleofasciculus	chthonoplastes	SAG-2210
0	25	0	29	0	0	0	0	0	0	0	0	Bacteria	Cyanobacteria	Cyanophyceae	Chroococcales	Chroococcaceae	Dactylococcopsis	Dactylococcopsis	sp.	PCC-8305
0	0	11	0	0	43	11	34	0	8	0	0	Bacteria	Cyanobacteria	Cyanophyceae	Synechococcales	Pseudanabaenaceae	Geitlerinema	Geitlerinema	sp.	PCC-7105
0	0	0	0	43	0	0	0	0	0	0	0	Bacteria	Cyanobacteria	Cyanophyceae	Chroococcales	Chroococcaceae	Gloeocapsa	Gloeocapsa	sp.	
0	0	0	0	23	0	0	0	0	0	0	0	Bacteria	Cyanobacteria	Cyanophyceae	Chroococcales	Chroococcaceae	Gloeocapsopsis	Gloeocapsopsis	crepidinum	BDU-20121
0	30	0	0	0	0	0	0	0	0	0	0	Bacteria	Cyanobacteria	Cyanophyceae	Chroococcales	Chroococcaceae	Gloeothece	Gloeothece	sp.	KO68DGA
0	0	0	0	0	0	0	0	24	16	0	0	Bacteria	Cyanobacteria	Cyanophyceae	Synechococcales	Leptolyngbyaceae	Halomicronema	Halomicronema	sp.	C2206
0	26	0	0	0	0	0	0	0	0	0	0	Bacteria	Cyanobacteria	Cyanophyceae	Synechococcales	Leptolyngbyaceae	Halomicronema	Halomicronema	sp.	C2206
0	0	0	40	0	0	0	0	0	0	0	0	Bacteria	Cyanobacteria	Cyanophyceae	Spirulinales	Spirulinaceae	Halospirulina	Halospirulina	sp.	CCC-Baja-95-Cl.2
0	24	0	0	0	0	0	0	0	0	0	0	Bacteria	Cyanobacteria	Cyanophyceae	Spirulinales	Spirulinaceae	Halospirulina	Halospirulina	sp.	CCC-Baja-95-Cl.2
0	0	17	0	0	0	0	0	0	0	0	0	Bacteria	Cyanobacteria	Cyanophyceae	Oscillatoriales	Microcoleaceae	Hydrocoleum	Hydrocoleum	lyngbyaceum	HBC7
0	0	0	0	0	0	0	0	0	0	0	59	Bacteria	Cyanobacteria	Cyanophyceae	Oscillatoriales	Oscillatoriaceae	Leptolyngbya	Leptolyngbya	sp.	CENA555
0	0	0	0	68	49	0	0	0	0	0	0	Bacteria	Cyanobacteria	Cyanophyceae	Synechococcales	Leptolyngbyaceae	Leptolyngbya	Leptolyngbya	sp.	
0	0	0	0	0	139	17	1511	1104	68	298	19	Bacteria	Cyanobacteria	Cyanophyceae	Oscillatoriales	Oscillatoriaceae	Lyngbya	Lyngbya	sp.	PCC-7419
0	0	0	0	0	0	0	36	10	0	0	0	Bacteria	Cyanobacteria	Cyanophyceae	Oscillatoriales	Oscillatoriaceae	Lyngbya	Lyngbya	sp.	PCC-7419
108	38	0	32	0	0	0	0	0	0	0	0	Bacteria	Cyanobacteria	Cyanophyceae	Synechococcales	Leptolyngbyaceae	MBIC10086			
0	0	0	0	22	0	0	0	0	0	0	0	Bacteria	Cyanobacteria	Cyanophyceae	Synechococcales	Leptolyngbyaceae	MBIC10086			
0	0	0	0	25	23	0	31	38	44	0	29	Bacteria	Cyanobacteria	Cyanophyceae	Synechococcales	Leptolyngbyaceae	Nodosilinea	Nodosilinea	sp.	PCC-7104
0	0	0	0	21	49	0	25	45	0	0	16	Bacteria	Cyanobacteria	Cyanophyceae	Synechococcales	Leptolyngbyaceae	Nodosilinea	Nodosilinea	sp.	PCC-7104
0	0	0	0	0	29	29	0	0	0	0	0	Bacteria	Cyanobacteria	Cyanophyceae	Oscillatoriales	Oscillatoriaceae	Oscillatoria	Oscillatoria	sp.	PCC-6304
76	46	0	46	0	0	0	0	0	0	0	0	Bacteria	Cyanobacteria	Cyanophyceae	Oscillatoriales	Oscillatoriaceae	Phormidium	Phormidium	sp.	
0	0	0	0	35	78	0	77	122	72	43	0	Bacteria	Cyanobacteria	Cyanophyceae	Oscillatoriales	Oscillatoriaceae	Phormidium	Phormidium	sp.	
0	0	0	0	54	85	0	0	0	0	0	0	Bacteria	Cyanobacteria	Cyanophyceae	Oscillatoriales	Oscillatoriaceae	Phormidium	Phormidium	sp.	
0	0	0	39	0	0	0	0	0	0	0	0	Bacteria	Cyanobacteria	Cyanophyceae	Synechococcales	Pseudanabaenaceae	Pseudanabaena	Pseudanabaena	persicina	TAU-MAC-1415
0	13	0	0	0	0	0	0	0	0	0	0	Bacteria	Cyanobacteria	Cyanophyceae	Oscillatoriales	Coleofasciculaceae	Roseofilum	Roseofilum	sp.	A01-A
0	0	0	37	0	0	0	0	0	0	0	0	Bacteria	Cyanobacteria	Cyanophyceae	Spirulinales	Spirulinaceae	Spirulina	Spirulina	sp.	CCC-Snake-P-Y-85
0	0	0	0	0	0	0	23	0	0	0	0	Bacteria	Cyanobacteria	Cyanophyceae	Oscillatoriales	Microcoleaceae	Symploca	Symploca	sp.	PCC-8002
0	0	0	0	0	0	0	116	50	0	0	48	Bacteria	Cyanobacteria	Cyanophyceae	Synechococcales	Merismopediaceae	Synechocystis	Synechocystis	pevalekii	BDHKU-20401
0	79	0	0	0	0	0	0	0	0	0	0	Bacteria	Cyanobacteria	Cyanophyceae	Chroococcales	Chroococcaceae				
26	0	0	0	0	0	0	0	0	0	0	0	Bacteria	Cyanobacteria	Cyanophyceae	Chroococcales	Chroococcaceae				
8	21	0	0	0	0	0	0	0	0	0	0	Bacteria	Cyanobacteria	Cyanophyceae	Chroococcales	Chroococcaceae				
0	0	0	0	0	39	0	63	40	41	48	0	Bacteria	Cyanobacteria	Cyanophyceae	Oscillatoriales	Coleofasciculaceae				
0	0	0	0	0	0	0	61	76	0	32	0	Bacteria	Cyanobacteria	Cyanophyceae	Oscillatoriales	Desertifilaceae				
0	64	0	23	0	0	0	0	0	0	0	0	Bacteria	Cyanobacteria	Cyanophyceae	Oscillatoriales	Oscillatoriaceae				
0	0	0	0	0	56	0	65	0	0	0	0	Bacteria	Cyanobacteria	Cyanophyceae	Oscillatoriales	Oscillatoriaceae				
0	0	0	0	0	0	0	34	0	0	0	0	Bacteria	Cyanobacteria	Cyanophyceae	Oxyphotobacteria Incertae Sedis	unclassified_Bacteria				
0	0	0	0	51	148	0	369	144	64	59	107	Bacteria	Cyanobacteria	Cyanophyceae	Synechococcales	Leptolyngbyaceae				
0	0	0	0	0	72	0	0	0	0	0	0	Bacteria	Cyanobacteria	Cyanophyceae	Synechococcales	Leptolyngbyaceae				
0	0	0	0	55	44	0	86	0	0	0	51	Bacteria	Cyanobacteria	Cyanophyceae	Synechococcales	Leptolyngbyaceae				
0	0	0	52	55	48	0	0	27	0	27	35	Bacteria	Cyanobacteria	Cyanophyceae	Synechococcales	Leptolyngbyaceae				
7	0	0	18	0	0	0	24	78	19	0	10	Bacteria	Cyanobacteria	Cyanophyceae	unclassified_Bacteria	unclassified_Bacteria				
0	32	0	0	0	14	0	0	0	0	0	0	Bacteria	Cyanobacteria	Cyanophyceae	unclassified_Bacteria	unclassified_Bacteria				
72	259	0	0	106	145	113	167	164	115	37	108	Bacteria	Cyanobacteria	Cyanophyceae	unclassified_Bacteria	unclassified_Bacteria				
0	0	0	0	8	0	0	0	26	0	0	0	Bacteria	Dadabacteria	Dadabacteriia	Dadabacteriales	unclassified_Bacteria				
0	0	0	0	0	0	0	0	0	0	18	0	Bacteria	Dadabacteria	Dadabacteriia	Dadabacteriales	unclassified_Bacteria				
9	0	0	0	0	0	9	0	0	0	0	0	Bacteria	Deferrisomatota	Defferrisomatia	Defferrisomatales	Defferrisomataceae	Deferrisoma	Deferrisoma	sp.	
0	0	0	0	0	0	0	0	7	0	11	0	Bacteria	Deferrisomatota	Defferrisomatia	Defferrisomatales	Defferrisomataceae	Deferrisoma	Deferrisoma	sp.	
0	14	0	17	0	0	0	22	0	0	0	0	Bacteria	Deinococcota	Deinococci	Deinococcales	Trueperaceae	Truepera	Truepera	sp.	
0	0	0	0	0	0	44	38	34	0	0	14	Bacteria	Deinococcota	Deinococci	Deinococcales	Trueperaceae	Truepera	Truepera	sp.	YC-ZSS-LKJ140
0	0	0	65	0	0	0	0	0	0	0	0	Bacteria	Deinococcota	Deinococci	Deinococcales	Trueperaceae	Truepera	Truepera	sp.	
0	0	0	0	0	0	0	0	4	0	9	0	Bacteria	Dependentiae	Babeliae	Babeliales	Vermiphilaceae				
0	0	0	29	0	0	0	0	0	0	0	0	Bacteria	Dependentiae	Babeliae	Babeliales	unclassified_Bacteria				
0	17	0	0	0	0	0	0	0	0	0	0	Bacteria	Desulfobacterota	Desulfobulbia	Desulfobulbales	Desulfobulbaceae	Candidatus Electrothrix			
0	55	0	0	0	0	0	0	0	0	0	0	Bacteria	Desulfobacterota	Desulfobulbia	Desulfobulbales	Desulfobulbaceae	Candidatus Electrothrix			
0	0	0	0	0	0	0	61	9	0	0	33	Bacteria	Desulfobacterota	Desulfobacteria	Desulfobacterales	Desulfosarcinaceae	Desulfatibacillum	Desulfatibacillum	sp.	
0	0	0	0	0	0	0	0	0	0	0	42	Bacteria	Desulfobacterota	Desulfobacteria	Desulfatiglandales	Desulfatiglandaceae	Desulfatiglans	Desulfatiglans	sp.	
0	0	0	0	0	0	17	0	0	0	0	0	Bacteria	Desulfobacterota	Desulfobacteria	Desulfatiglandales	Desulfatiglandaceae	Desulfatiglans	Desulfatiglans	sp.	
0	29	0	0	0	0	0	0	0	46	45	0	Bacteria	Desulfobacterota	Desulfobacteria	Desulfatiglandales	Desulfatiglandaceae	Desulfatiglans	Desulfatiglans	sp.	NaphS6
0	0	0	0	0	34	48	0	13	0	0	0	Bacteria	Desulfobacterota	Desulfobacteria	Desulfatiglandales	Desulfatiglandaceae	Desulfatiglans	Desulfatiglans	sp.	
0	0	0	33	0	0	0	0	0	0	0	0	Bacteria	Desulfobacterota	Desulfobacteria	Desulfobacterales	Desulfobacteraceae	Desulfobacter	Desulfobacter	sp.	
0	21	0	147	0	0	0	0	0	0	0	0	Bacteria	Desulfobacterota	Desulfobacteria	Desulfobacterales	Desulfobacteraceae	Desulfobacterium	Desulfobacterium	sp.	
0	0	0	24	0	0	0	0	0	0	0	0	Bacteria	Desulfobacterota	Desulfobacteria	Desulfobacterales	Desulfobacteraceae	Desulfobacterium	Desulfobacterium	sp.	
0	0	0	188	0	0	0	0	0	0	0	0	Bacteria	Desulfobacterota	Desulfobulbia	Desulfobulbales	Desulfobulbaceae	Desulfobulbus	Desulfobulbus	sp.	
0	0	0	0	0	14	0	0	0	0	0	0	Bacteria	Desulfobacterota	Desulfobulbia	Desulfobulbales	Desulfocapsaceae	Desulfofustis	Desulfofustis	sp.	
0	0	0	0	54	39	0	0	0	45	0	0	Bacteria	Desulfobacterota	Desulfobulbia	Desulfobulbales	Desulfocapsaceae	Desulfofustis	Desulfofustis	sp.	
0	0	0	0	44	0	0	0	0	0	0	0	Bacteria	Desulfobacterota	Desulfomonilia	Desulfomonilales	Desulfomonilaceae	Desulfomonile	Desulfomonile	sp.	
0	0	0	0	25	0	0	0	0	0	0	0	Bacteria	Desulfobacterota	Desulfomonilia	Desulfomonilales	Desulfomonilaceae	Desulfomonile	Desulfomonile	sp.	
0	0	0	0	0	64	0	0	47	0	0	0	Bacteria	Desulfobacterota	Desulfovibrionia	Desulfovibrionales	Desulfonatronaceae	Desulfonatronum	Desulfonatronum	alkalitolerans	
0	89	0	67	16	41	62	34	52	42	27	118	Bacteria	Desulfobacterota	Desulfobulbia	Desulfobulbales	Desulfocapsaceae	Desulfopila	Desulfopila	sp.	
0	32	0	0	0	0	0	0	0	0	0	0	Bacteria	Desulfobacterota	Desulfobulbia	Desulfobulbales	Desulfocapsaceae	Desulforhopalus	Desulforhopalus	sp.	
0	0	0	0	45	68	0	0	0	0	48	75	Bacteria	Desulfobacterota	Desulfobacteria	Desulfobacterales	Desulfosarcinaceae	Desulfosalsimonas	Desulfosalsimonas	sp.	
76	0	0	151	0	0	0	0	0	0	0	0	Bacteria	Desulfobacterota	Desulfobacteria	Desulfobacterales	Desulfosarcinaceae	Desulfosarcina	Desulfosarcina	sp.	
32	30	48	101	0	22	0	0	0	0	0	0	Bacteria	Desulfobacterota	Desulfobacteria	Desulfobacterales	Desulfosarcinaceae	Desulfosarcina	Desulfosarcina	sp.	
0	57	0	85	87	90	0	0	51	51	23	33	Bacteria	Desulfobacterota	Desulfobacteria	Desulfobacterales	Desulfobacteraceae	Desulfotignum	Desulfotignum	sp.	
0	0	0	0	0	0	35	0	56	69	17	85	Bacteria	Desulfobacterota	Desulfovibrionia	Desulfovibrionales	Desulfohalobiaceae	Desulfovermiculus	Desulfovermiculus	sp.	
0	43	0	0	0	0	0	0	0	0	0	0	Bacteria	Desulfobacterota	Desulfovibrionia	Desulfovibrionales	Desulfohalobiaceae	Desulfovermiculus	Desulfovermiculus	sp.	
0	0	0	103	133	177	29	31	119	103	57	145	Bacteria	Desulfobacterota	Desulfovibrionia	Desulfovibrionales	Desulfovibrionaceae	Desulfovibrio	Desulfovibrio	sp.	
0	17	6	32	57	49	0	0	0	0	0	0	Bacteria	Desulfobacterota	Desulfovibrionia	Desulfovibrionales	Desulfovibrionaceae	Desulfovibrio	Desulfovibrio	halophilus	
0	0	0	33	0	0	0	0	0	0	0	0	Bacteria	Desulfobacterota	Desulfovibrionia	Desulfovibrionales	Desulfovibrionaceae	Desulfovibrio	Desulfovibrio	hontreensis	
0	0	0	0	19	14	0	0	0	0	0	0	Bacteria	Desulfobacterota	Desulfovibrionia	Desulfovibrionales	Desulfovibrionaceae	Desulfovibrio	Desulfovibrio	sp.	L21-Syr-AB
0	0	0	0	24	20	0	0	0	0	0	0	Bacteria	Desulfobacterota	Desulfovibrionia	Desulfovibrionales	Desulfovibrionaceae	Desulfovibrio	Desulfovibrio	sp.	Ecwsrb011
0	0	0	0	0	75	0	0	0	0	0	0	Bacteria	Desulfobacterota	Desulfovibrionia	Desulfovibrionales	Desulfovibrionaceae	Desulfovibrio	Pseudodesulfovibrio	profundus	
0	0	0	13	0	0	0	0	0	0	0	0	Bacteria	Desulfobacterota	Desulfovibrionia	Desulfovibrionales	Desulfovibrionaceae	Desulfovibrio	Desulfovibrio	sp.	
0	0	0	20	201	141	0	19	0	53	0	95	Bacteria	Desulfobacterota	Desulfovibrionia	Desulfovibrionales	Desulfovibrionaceae	Desulfovibrio	Desulfovibrio	sp.	
0	0	0	0	0	0	0	0	27	0	0	0	Bacteria	Desulfobacterota	Desulfuromonadia	Desulfuromonadia	Geothermobacteraceae	Geothermobacter	Geothermobacter	sp.	
0	0	0	0	0	0	18	0	0	0	0	0	Bacteria	Desulfobacterota	Desulfuromonadia	Desulfuromonadia	Geothermobacteraceae	Geothermobacter	Geothermobacter	sp.	
0	0	20	0	51	0	134	44	0	0	0	5	Bacteria	Desulfobacterota	Desulfuromonadia	Bradymonadales	Bradymonadaceae	Marinobacter	Marinobacter	sp.	
0	0	0	0	0	0	96	0	11	0	0	0	Bacteria	Desulfobacterota	Desulfobulbia	Desulfobulbales	Desulfurivibrionaceae	MSBL7			
0	0	0	0	0	0	0	0	0	17	0	0	Bacteria	Desulfobacterota	Desulfobacteria	Desulfobacterales	Desulfosarcinaceae	SEEP-SRB1			S7086C20
0	62	0	0	0	0	0	0	0	0	0	0	Bacteria	Desulfobacterota	Desulfobacteria	Desulfobacterales	Desulfosarcinaceae	Sva0081 sediment group			
0	0	0	39	0	0	101	0	31	0	0	40	Bacteria	Desulfobacterota	Desulfobacteria	Desulfobacterales	Desulfosarcinaceae	Sva0081 sediment group			
57	0	0	0	0	0	0	0	0	0	0	0	Bacteria	Desulfobacterota	Desulfobacteria	Desulfobacterales	Desulfosarcinaceae	Sva0081 sediment group			
0	0	0	0	0	0	0	0	0	11	0	0	Bacteria	Desulfobacterota	Desulfobacteria	Desulfobacterales	Desulfosarcinaceae	Sva0081 sediment group			
25	32	63	28	136	169	47	83	140	168	71	0	Bacteria	Desulfobacterota	Desulfuromonadia	Bradymonadales	Bradymonadaceae				
0	0	0	0	50	0	18	13	14	0	3	0	Bacteria	Desulfobacterota	Desulfuromonadia	Bradymonadales	Bradymonadaceae				
0	21	138	0	160	275	0	388	520	475	313	350	Bacteria	Desulfobacterota	Desulfuromonadia	Bradymonadales	Bradymonadaceae				
0	0	0	0	0	0	0	0	0	33	0	34	Bacteria	Desulfobacterota	Desulfuromonadia	Bradymonadales	Bradymonadaceae				
0	0	0	9	0	47	68	0	4	27	0	0	Bacteria	Desulfobacterota	Desulfuromonadia	Bradymonadales	Bradymonadales				
32	71	0	0	0	0	0	38	0	0	0	0	Bacteria	Desulfobacterota	Desulfuromonadia	Bradymonadales	Bradymonadales				GMD14H09
51	0	0	0	14	0	26	125	78	19	91	0	Bacteria	Desulfobacterota	Desulfuromonadia	Bradymonadales	Bradymonadales				
0	0	0	0	44	0	0	126	34	50	33	95	Bacteria	Desulfobacterota	Desulfuromonadia	Bradymonadales	Bradymonadales				

27	0	0	58	0	8	0	0	0	3	9	0	Bacteria	Desulfobacterota	Desulfuromonadia	Bradymonadales	Bradymonadales				
0	0	0	0	0	0	0	0	22	0	0	0	Bacteria	Desulfobacterota	Desulfuromonadia	Bradymonadales	unclassified_Bacteria				
0	0	0	59	0	37	0	0	0	0	29	0	Bacteria	Desulfobacterota	Desulfobacteria	Desulfobacterales	Desulfobacteraceae				
0	0	0	0	23	23	0	0	0	0	0	0	Bacteria	Desulfobacterota	Desulfobacteria	Desulfobacterales	Desulfobacteraceae				
0	0	0	0	0	0	0	0	52	0	0	0	Bacteria	Desulfobacterota	Desulfobacteria	Desulfobacterales	Desulfobacterales				
0	24	0	0	41	117	0	0	0	110	56	0	Bacteria	Desulfobacterota	Desulfobacteria	Desulfobacterales	Desulfococcaceae				
0	45	0	121	0	0	27	0	61	0	0	0	Bacteria	Desulfobacterota	Desulfobacteria	Desulfobacterales	Desulfococcaceae				
0	43	0	35	125	179	0	0	69	63	83	63	Bacteria	Desulfobacterota	Desulfobacteria	Desulfobacterales	Desulfococcaceae				
0	0	0	33	0	0	0	0	0	0	0	0	Bacteria	Desulfobacterota	Desulfobacteria	Desulfobacterales	Desulfococcaceae				
0	0	0	0	0	0	0	0	0	22	0	0	Bacteria	Desulfobacterota	Desulfobacteria	Desulfobacterales	Desulfococcaceae				
30	97	33	27	82	94	0	0	14	36	54	26	Bacteria	Desulfobacterota	Desulfobacteria	Desulfobacterales	Desulfococcaceae				
0	99	0	97	37	43	247	0	66	43	67	155	Bacteria	Desulfobacterota	Desulfobacteria	Desulfobacterales	Desulfosarcinaceae				
0	0	0	0	0	0	0	0	0	0	0	26	Bacteria	Desulfobacterota	Desulfobacteria	Desulfobacterales	Desulfosarcinaceae				
0	0	0	0	67	97	77	0	60	0	95	81	Bacteria	Desulfobacterota	Desulfobacteria	Desulfobacterales	Desulfosarcinaceae				
0	0	0	0	0	0	17	0	0	0	0	0	Bacteria	Desulfobacterota	Desulfobacteria	Desulfobacterales	Desulfosarcinaceae				
0	0	0	0	53	21	0	0	0	0	46	0	Bacteria	Desulfobacterota	Desulfobacteria	Desulfobacterales	Desulfosarcinaceae				
31	111	0	122	164	208	329	111	184	282	254	294	Bacteria	Desulfobacterota	Desulfobacteria	Desulfobacterales	unclassified_Bacteria				
0	77	0	0	0	0	0	0	0	0	0	0	Bacteria	Desulfobacterota	Desulfobulbia	Desulfobulbales	Desulfobulbaceae				
0	0	0	0	0	0	61	0	0	0	0	0	Bacteria	Desulfobacterota	Desulfobulbia	Desulfobulbales	Desulfobulbaceae				
44	69	0	45	30	167	0	0	0	0	0	0	Bacteria	Desulfobacterota	Desulfobulbia	Desulfobulbales	Desulfocapsaceae				
0	0	0	133	0	0	0	0	0	0	0	0	Bacteria	Desulfobacterota	Desulfobulbia	Desulfobulbales	Desulfocapsaceae				
46	64	31	64	0	0	0	0	0	0	0	0	Bacteria	Desulfobacterota	Desulfobulbia	Desulfobulbales	Desulfocapsaceae				
0	0	0	0	151	55	0	28	51	27	56	179	Bacteria	Desulfobacterota	Desulfovibrionia	Desulfovibrionales	Desulfovibrionaceae				
0	0	0	39	0	0	0	0	0	0	0	0	Bacteria	Desulfobacterota	Desulfovibrionia	Desulfovibrionales	Desulfovibrionaceae				
0	0	0	0	91	183	199	0	231	91	86	76	Bacteria	Desulfobacterota	Desulfovibrionia	Desulfovibrionales	unclassified_Bacteria				
0	21	0	0	0	0	0	0	0	11	0	0	Bacteria	Desulfobacterota	Desulfuromonadia	Desulfuromonadia	Geopsychrobacteraceae				
0	0	0	0	20	15	0	0	18	0	11	20	Bacteria	Desulfobacterota	Dissulfuribacteria	Dissulfuribacterales	Dissulfuribacteraceae				
0	0	0	0	0	0	0	29	0	0	0	0	Bacteria	Desulfobacterota	Desulfuromonadia	PB19	unclassified_Bacteria				
0	0	0	0	0	0	0	0	0	10	0	0	Bacteria	Desulfobacterota	Desulfuromonadia	PB19	unclassified_Bacteria				
0	0	0	0	0	29	0	0	36	0	0	0	Bacteria	Desulfobacterota	Syntrophobacteria	Syntrophobacterales	Syntrophobacteraceae				
0	0	0	0	0	0	20	0	0	0	0	0	Bacteria	Desulfobacterota	Syntrophobacteria	Syntrophobacterales	Syntrophobacteraceae				
0	0	0	0	0	0	86	0	0	0	0	0	Bacteria	Desulfobacterota	Syntrophobacteria	Syntrophobacterales	Syntrophobacteraceae				
0	0	0	0	0	0	30	0	0	0	0	0	Bacteria	Desulfobacterota	Syntrophobacteria	Syntrophobacterales	Syntrophobacteraceae				
0	0	0	5	0	0	0	0	85	0	0	31	Bacteria	Desulfobacterota	Desulfuromonadia	unclassified_Bacteria	unclassified_Bacteria				
0	0	0	0	0	0	21	48	0	4	0	25	Bacteria	Desulfobacterota	unclassified_Bacteria	unclassified_Bacteria	unclassified_Bacteria				
0	0	0	0	0	0	28	0	0	0	0	0	Bacteria	Elusimicrobiota	Lineage IIb	unclassified_Bacteria	unclassified_Bacteria				
0	0	0	0	0	0	0	9	0	0	0	0	Bacteria	Entotheonellaeota	Entotheonellia	Entotheonellales	Entotheonellaceae				
0	0	0	12	0	0	0	0	0	0	0	0	Bacteria	Fermentibacterota	Fermentibacteria	Fermentibacterales	Fermentibacteraceae				
0	0	0	0	0	0	0	0	0	53	0	0	Bacteria	Fibrobacterota	Fibrobacteria	Fibrobacterales	unclassified_Bacteria	09D2Z48			
0	0	0	0	0	0	0	0	30	14	0	16	Bacteria	Fibrobacterota	Fibrobacteria	Fibrobacterales	unclassified_Bacteria	B122			
0	0	0	10	0	0	0	0	0	0	0	0	Bacteria	Fibrobacterota	Fibrobacteria	Fibrobacterales	unclassified_Bacteria	B18			
0	0	0	24	0	24	0	0	27	16	0	39	Bacteria	Fibrobacterota	Fibrobacteria	Fibrobacterales	unclassified_Bacteria	MAT-CR-H6-H10			
0	0	0	0	0	10	0	0	0	12	0	51	Bacteria	Fibrobacterota	Fibrobacteria	Fibrobacterales	unclassified_Bacteria	SBXY-748			
0	0	0	0	9	47	38	0	22	0	0	48	Bacteria	Fibrobacterota	Fibrobacteria	Fibrobacterales	unclassified_Bacteria	SBYC			
0	0	0	0	0	29	0	0	0	0	0	0	Bacteria	Fibrobacterota	Fibrobacteria	Fibrobacterales	unclassified_Bacteria	SBYZ-1017			
0	0	0	0	0	15	0	0	0	0	0	0	Bacteria	Fibrobacterota	Fibrobacteria	Fibrobacterales	unclassified_Bacteria	TG3			
0	7	0	0	0	0	0	0	12	41	25	0	Bacteria	Fibrobacterota	Fibrobacteria	Fibrobacterales	Fibrobacteraceae				
12	0	0	0	0	0	0	0	0	0	0	0	Bacteria	Fibrobacterota	Fibrobacteria	Fibrobacterales	Fibrobacteraceae				
0	0	0	0	0	0	0	31	0	0	0	0	Bacteria	Fibrobacterota	Fibrobacteria	Fibrobacterales	Fibrobacteraceae				
0	0	0	0	0	18	0	30	22	0	0	30	Bacteria	Fibrobacterota	Fibrobacteria	Fibrobacterales	Fibrobacteraceae				
0	0	0	0	0	0	0	0	10	0	0	0	Bacteria	Fibrobacterota	Fibrobacteria	Fibrobacterales	Fibrobacterales				
0	0	0	0	0	0	0	0	0	22	0	27	Bacteria	Fibrobacterota	Fibrobacteria	Fibrobacterales	unclassified_Bacteria				
0	0	18	0	0	0	0	0	0	0	0	0	Bacteria	Firmicutes	Clostridia	Peptostreptococcales-Tissierellales	Peptostreptococcales-Tissierellales	Alkaliphilus	Alkaliphilus	halophilus	
0	0	144	0	0	0	207	172	336	232	108	149	Bacteria	Firmicutes	Bacilli	Bacillales	Bacillaceae	Bacillus	Bacillus	sp.	
0	0	53	0	0	0	0	0	0	0	0	0	Bacteria	Firmicutes	Bacilli	Bacillales	Bacillaceae	Bacillus	Bacillus	solimangrovi	
0	0	0	0	0	0	0	0	0	0	0	61	Bacteria	Firmicutes	Bacilli	Bacillales	Bacillaceae	Bacillus	Bacillus	sp.	HME7618
0	0	77	0	0	0	0	0	0	0	0	0	Bacteria	Firmicutes	Clostridia	Peptostreptococcales-Tissierellales	Peptostreptococcales-Tissierellales	Brassicibacter	Brassicibacter	sp.	
0	0	0	0	0	0	0	15	0	0	0	24	Bacteria	Firmicutes	Bacilli	Brevibacillales	Brevibacillaceae	Brevibacillus	Brevibacillus	sp.	
0	0	0	0	0	0	0	0	134	0	0	74	Bacteria	Firmicutes	Clostridia	Peptostreptococcales-Tissierellales	Caminicellaceae	Caminicella	Caminicella	sp.	
0	0	92	0	0	0	65	0	0	53	0	47	Bacteria	Firmicutes	Clostridia	Peptostreptococcales-Tissierellales	Caminicellaceae	Caminicella	Caminicella	sp.	
0	0	106	0	0	0	0	0	0	0	0	40	Bacteria	Firmicutes	Clostridia	Peptostreptococcales-Tissierellales	Peptostreptococcales-Tissierellales	Clostridiisalibacter	Clostridiisalibacter	sp.	
0	0	49	0	0	30	0	66	0	0	0	3	Bacteria	Firmicutes	Clostridia	Peptostreptococcales-Tissierellales	Peptostreptococcales-Tissierellales	Clostridiisalibacter	Clostridiisalibacter	sp.	YC-ZSS-LKJ129
0	0	188	0	0	0	0	0	0	0	0	0	Bacteria	Firmicutes	Clostridia	Peptostreptococcales-Tissierellales	Peptostreptococcales-Tissierellales	Clostridiisalibacter	Clostridiisalibacter	sp.	
0	0	150	0	61	0	34	0	38	0	0	57	Bacteria	Firmicutes	Clostridia	Peptostreptococcales-Tissierellales	Peptostreptococcales-Tissierellales	Clostridiisalibacter	Clostridiisalibacter	sp.	
0	0	32	0	0	0	0	0	0	0	0	5	Bacteria	Firmicutes	Clostridia	Clostridiales	Clostridiaceae	Clostridium	Clostridium	ganghwense	
0	0	0	0	0	0	0	14	0	0	0	0	Bacteria	Firmicutes	Clostridia	Clostridiales	Clostridiaceae	Clostridium	Clostridium	sp.	
0	0	0	0	0	0	0	92	87	0	0	0	Bacteria	Firmicutes	Clostridia	Peptostreptococcales-Tissierellales	Thermotaleaceae	Crassaminicella	Crassaminicella	sp.	
0	0	0	0	0	0	0	0	12	0	0	0	Bacteria	Firmicutes	Clostridia	Lachnospirales	Defluviitaleaceae	Defluviitalea	Defluviitalea	sp.	
0	0	6	0	0	0	0	0	0	0	0	0	Bacteria	Firmicutes	Desulfotomaculia	Desulfotomaculales	Desulfotomaculales	Desulfohalotomaculum	Desulfohalotomaculum	halophilum	
0	0	0	0	0	0	0	0	15	0	0	0	Bacteria	Firmicutes	Desulfotomaculia	Desulfotomaculales	Desulfotomaculales	Desulfotomaculum	Desulfotomaculum	sp.	
0	0	0	0	8	0	0	0	0	0	0	0	Bacteria	Firmicutes	Clostridia	Peptostreptococcales-Tissierellales	Dethiosulfatibacteraceae	Dethiosulfatibacter	Dethiosulfatibacter	sp.	
0	0	0	106	0	0	0	0	0	0	0	0	Bacteria	Firmicutes	Clostridia	Peptostreptococcales-Tissierellales	Fusibacteraceae	Fusibacter	Fusibacter	sp.	
0	6	18	44	0	0	0	0	0	0	0	0	Bacteria	Firmicutes	Clostridia	Peptostreptococcales-Tissierellales	Fusibacteraceae	Fusibacter	Fusibacter	sp.	NP25
0	0	0	45	0	0	0	0	0	0	0	0	Bacteria	Firmicutes	Clostridia	Peptostreptococcales-Tissierellales	Fusibacteraceae	Fusibacter	Fusibacter	sp.	
0	152	467	112	20	78	0	0	0	0	0	0	Bacteria	Firmicutes	Clostridia	Peptostreptococcales-Tissierellales	Fusibacteraceae	Fusibacter	Fusibacter	sp.	
0	54	90	0	0	0	98	0	0	0	0	0	Bacteria	Firmicutes	Bacilli	Bacillales	Bacillaceae	Halobacillus	Halobacillus	sp.	
0	0	85	0	0	0	0	0	0	0	0	0	Bacteria	Firmicutes	Bacilli	Haloplasmatales	Haloplasmataceae	Haloplasma	Haloplasma	sp.	
0	0	9	0	0	0	0	0	0	0	0	0	Bacteria	Firmicutes	Bacilli	Haloplasmatales	Haloplasmataceae	Haloplasma	Haloplasma	sp.	YC-ZSS-LKJ118
0	0	64	0	14	0	0	0	0	0	0	0	Bacteria	Firmicutes	Bacilli	Haloplasmatales	Haloplasmataceae	Haloplasma	Haloplasma	sp.	
0	0	54	0	0	0	0	90	0	30	0	83	Bacteria	Firmicutes	Bacilli	Haloplasmatales	Haloplasmataceae	Haloplasma	Haloplasma	sp.	
0	0	71	0	0	0	0	0	0	0	0	0	Bacteria	Firmicutes	Bacilli	Haloplasmatales	Haloplasmataceae	Haloplasma	Haloplasma	sp.	
0	0	0	40	0	0	0	0	0	0	0	0	Bacteria	Firmicutes	Bacilli	Izemoplasmatales	Izemoplasmataceae	Izimaplasma	Izimaplasma	sp.	
0	0	0	0	7	18	0	0	0	5	0	0	Bacteria	Firmicutes	Bacilli	Izemoplasmatales	Izemoplasmataceae	Izimaplasma	Izimaplasma	sp.	HR1
0	24	0	0	0	10	0	0	0	0	0	0	Bacteria	Firmicutes	Bacilli	Izemoplasmatales	Izemoplasmataceae	Izimaplasma	Izimaplasma	sp.	
0	23	0	28	0	0	0	0	0	0	0	0	Bacteria	Firmicutes	Bacilli	Izemoplasmatales	Izemoplasmataceae	Izimaplasma	Izimaplasma	sp.	
0	0	0	0	0	0	0	0	0	0	0	7	Bacteria	Firmicutes	Limnochordia	unclassified_Bacteria	unclassified_Bacteria	MBA03			
0	0	51	0	0	0	0	0	0	0	0	0	Bacteria	Firmicutes	Clostridia	Peptostreptococcales-Tissierellales	Caminicellaceae	Paramaledivibacter	Paramaledivibacter	sp.	
0	0	65	0	0	0	0	0	0	0	0	46	Bacteria	Firmicutes	Bacilli	Bacillales	Bacillaceae	Pontibacillus	Pontibacillus	sp.	
0	0	39	0	0	0	0	0	0	0	0	0	Bacteria	Firmicutes	Bacilli	Bacillales	Bacillaceae	Salimicrobium	Salimicrobium	sp.	
0	0	96	0	0	0	0	0	0	0	0	0	Bacteria	Firmicutes	Clostridia	Peptostreptococcales-Tissierellales	Peptostreptococcales-Tissierellales	Sporosalibacterium	Sporosalibacterium	sp.	
0	0	24	0	0	0	0	0	0	0	0	0	Bacteria	Firmicutes	Clostridia	Peptostreptococcales-Tissierellales	Peptostreptococcaceae	Tepidibacter	Tepidibacter	sp.	
0	0	0	0	0	0	58	46	0	0	0	25	Bacteria	Firmicutes	Bacilli	Alicyclobacillales	Alicyclobacillaceae	Tumebacillus	Tumebacillus	sp.	
0	0	0	0	0	0	0	0	0	0	0	15	Bacteria	Firmicutes	Bacilli	Alicyclobacillales	Alicyclobacillaceae	Tumebacillus	Tumebacillus	sp.	
0	0	0	27	0	0	0	0	0	0	0	0	Bacteria	Firmicutes	Clostridia	Clostridiales	Hungateiclostridiaceae	UCG-012			
0	0	0	0	0	0	0	0	0	0	23	0	Bacteria	Firmicutes	Clostridia	Clostridiales	Hungateiclostridiaceae	UCG-012			
0	0	224	0	7	0	7	0	0	69	0	149	Bacteria	Firmicutes	Bacilli	Bacillales	Bacillaceae				
0	0	25	0	62	0	47	93	0	0	56	0	Bacteria	Firmicutes	Bacilli	Bacillales	unclassified_Bacteria				
0	0	0	25	0	0	0	0	56	0	0	0	Bacteria	Firmicutes	Clostridia	Clostridiales	Clostridiaceae				
0	0	0	0	0	0	52	0	0	0	0	26	Bacteria	Firmicutes	Clostridia	Clostridiales	Hungateiclostridiaceae				
0	8	0	0	0	0	0	0	0	0	0	0	Bacteria	Firmicutes	Clostridia	Clostridiales	Hungateiclostridiaceae				saltmarsh-clone-LCP-84
0	15	0	0	0	15	0	0	0	0	0	0	Bacteria	Firmicutes	Bacilli	Izemoplasmatales	Izemoplasmataceae				
0	0	0	15	0	0	0	0	0	0	0	0	Bacteria	Firmicutes	Bacilli	Izemoplasmatales	Izemoplasmataceae				R4-81B
0	0	0	51	0	0	0	0	0	0	0	0	Bacteria	Firmicutes	Clostridia	Lachnospirales	Defluviitaleaceae				UCG-011
0	0	0	59	0	0	0	0	0	0	0	0	Bacteria	Firmicutes	Clostridia	Lachnospirales	Defluviitaleaceae				UCG-011
0	0	0	42	0	0	0	0	0	0	0	0	Bacteria	Firmicutes	Clostridia	Lachnospirales	Defluviitaleaceae				UCG-011
0	0	0	40	0	0	0	0	0	0	0	0	Bacteria	Firmicutes	Clostridia	Lachnospirales	Lachnospiraceae				
0	0	0	0	0	0	0	0	74	0	0	0	Bacteria	Firmicutes	Clostridia	Lachnospirales	unclassified_Bacteria				
0	0	26	0	0	0	0	0	0	0	0	0	Bacteria	Firmicutes	Clostridia	Peptococcales	Peptococcaceae				
0	0	0	0	0	0	0	0	24	0	0	0	Bacteria	Firmicutes	Clostridia	Peptococcales	Peptococcaceae				
0	0	24	0	0	0	0	0	0	0	0	0	Bacteria	Firmicutes	Clostridia	Peptostreptococcales-Tissierellales	Caminicellaceae				
0	0	0	59	0	0	0	0	25	0	0	0	Bacteria	Firmicutes	Clostridia	Peptostreptococcales-Tissierellales	Peptostreptococcaceae				
0	0	0	0	0	0	0	75	0	0	0	0	Bacteria	Firmicutes	Clostridia	Peptostreptococcales-Tissierellales	Peptostreptococcales-Tissierellales				
0	0	85	0	0	0	0	0	0	0	0	0	Bacteria	Firmicutes	Clostridia	Peptostreptococcales-Tissierellales	Peptostreptococcales-Tissierellales				
0	0	0	0	0	0	0	12	0	0	0	0	Bacteria	Firmicutes	Thermovenabulia	Thermovenabulales	unclassified_Bacteria				
0	0	0	0	11	0	0	52	71	8	34	83	Bacteria	Firmicutes	Thermovenabulia	Thermovenabulales	unclassified_Bacteria				
0	0	31	0	0	0	0	0	9	12	0	8	Bacteria	Firmicutes	Bacilli	unclassified_Bacteria	unclassified_Bacteria				
0	0	36	0	0	0	22	0	0	0	0	0	Bacteria	Firmicutes	Clostridia	unclassified_Bacteria	unclassified_Bacteria				
0	0	0	0	31	0	0	52	49	0	0	63	Bacteria	Firmicutes	unclassified_Bacteria	unclassified_Bacteria	unclassified_Bacteria				
0	0	0	0	50	30	0	0	0	20	25	0	Bacteria	FW113	unclassified_Bacteria	unclassified_Bacteria	unclassified_Bacteria				
0	0	0	0	0	0	0	0	11	0	0	0	Bacteria	Gemmatimonadota	unclassified_Bacteria	unclassified_Bacteria	unclassified_Bacteria	AKAU4049			
0	19	0	0	57	47	0	96	59	28	57	70	Bacteria	Gemmatimonadota	unclassified_Bacteria	unclassified_Bacteria	unclassified_Bacteria	BD2-11 terrestrial group			
0	0	0	0	0	0	33	66	0	0	27	35	Bacteria	Gemmatimonadota	unclassified_Bacteria	unclassified_Bacteria	unclassified_Bacteria	BD2-11 terrestrial group			YC-LK-LKJ11
71	5	0	0	89	24	143	236	296	57	97	84	Bacteria	Gemmatimonadota	unclassified_Bacteria	unclassified_Bacteria	unclassified_Bacteria	BD2-11 terrestrial group			
0	0	7	0	0	59	392	186	304	102	132	129	Bacteria	Gemmatimonadota	unclassified_Bacteria	unclassified_Bacteria	unclassified_Bacteria	BD2-11 terrestrial group			
74	0	0	0	55	48	55	129	0	28	40	25	Bacteria	Gemmatimonadota	unclassified_Bacteria	unclassified_Bacteria	unclassified_Bacteria	BD2-11 terrestrial group			
0	0	0	0	0	0	38	0	30	0	0	0	Bacteria	Gemmatimonadota	unclassified_Bacteria	unclassified_Bacteria	unclassified_Bacteria	BD2-11 terrestrial group			wb1-A18
0	0	0	0	6	0	0	0	0	0	0	0	Bacteria	Gemmatimonadota	unclassified_Bacteria	unclassified_Bacteria	unclassified_Bacteria	MD2902-B12			
0	30	0	0	0	0	0	0	0	0	0	0	Bacteria	Gemmatimonadota	unclassified_Bacteria	unclassified_Bacteria	unclassified_Bacteria	PAUC43f marine benthic group			saltmarsh-clone-LCP-68
0	0	0	0	119	0	191	247	189	0	273	104	Bacteria	Gemmatimonadota	unclassified_Bacteria	unclassified_Bacteria	unclassified_Bacteria	PAUC43f marine benthic group			
0	0	0	0	0	0	41	0	21	0	0	0	Bacteria	Gemmatimonadota	unclassified_Bacteria	unclassified_Bacteria	unclassified_Bacteria	S0134 terrestrial group			
0	0	0	4	0	0	0	0	0	0	0	0	Bacteria	Gemmatimonadota	Gemmatimonadetes	Gemmatimonadales	Gemmatimonadaceae				
0	0	0	0	0	0	45	0	0	0	0	0	Bacteria	Halanaerobiaeota	Halanaerobiia	Halanaerobiales	Halobacteroidaceae	Anoxybacter	Anoxybacter	fermentans	
0	0	0	0	0	0	0	0	0	0	0	13	Bacteria	Halanaerobiaeota	Halanaerobiia	Halanaerobiales	Halobacteroidaceae	Clostridia	Clostridia	sp.	
0	0	0	0	0	0	0	0	0	0	94	0	Bacteria	Halanaerobiaeota	Halanaerobiia	Halanaerobiales	Halobacteroidaceae	Halanaerobacter	Halanaerobacter	jeridensis	
0	3	0	0	0	0	0	0	0	0	0	0	Bacteria	Halanaerobiaeota	Halanaerobiia	Halanaerobiales	Halanaerobiaceae	Halanaerobium	Halanaerobium	sp.	
0	0	43	0	0	18	0	0	0	0	0	0	Bacteria	Halanaerobiaeota	Halanaerobiia	Halanaerobiales	Halanaerobiaceae	Halanaerobium	Halanaerobium	sp.	
0	0	17	0	0	0	0	0	0	0	0	0	Bacteria	Halanaerobiaeota	Halanaerobiia	Halanaerobiales	Halanaerobiaceae	Halanaerobium	Halanaerobium	sp.	
0	0	18	0	0	0	0	0	0	0	0	0	Bacteria	Halanaerobiaeota	Halanaerobiia	Halanaerobiales	Halanaerobiaceae	Halocella	Halocella	sp.	
0	0	0	0	0	0	14	0	0	0	0	0	Bacteria	Halanaerobiaeota	Halanaerobiia	Halanaerobiales	Halanaerobiaceae	Halocella	Halocella	sp.	
0	0	38	0	0	0	0	0	0	0	0	0	Bacteria	Halanaerobiaeota	Halanaerobiia	Halanaerobiales	Halobacteroidaceae	Halonatronum	Halonatronum	sp.	
0	0	0	0	0	6	0	0	0	0	0	0	Bacteria	Halanaerobiaeota	Halanaerobiia	Halanaerobiales	Halobacteroidaceae	Halonatronum	Halonatronum	saccharophilum	DSM-13868
0	0	91	0	0	0	0	0	26	0	0	0	Bacteria	Halanaerobiaeota	Halanaerobiia	Halanaerobiales	Halobacteroidaceae	Halothermothrix	Halothermothrix	sp.	
0	0	215	0	0	60	0	0	0	0	0	0	Bacteria	Halanaerobiaeota	Halanaerobiia	Halanaerobiales	Halobacteroidaceae	Orenia	Orenia	sp.	
0	0	150	0	0	0	0	0	0	0	0	0	Bacteria	Halanaerobiaeota	Halanaerobiia	Halanaerobiales	Halobacteroidaceae	Orenia	Orenia	sp.	

0	0	47	0	0	0	49	0	0	0	0	35	Bacteria	Halanaerobiaeota	Halanaerobiia	Halanaerobiales	Halobacteroidaceae	Orenia	Orenia	sp.	
0	0	281	0	0	0	0	0	0	0	0	0	Bacteria	Halanaerobiaeota	Halanaerobiia	Halanaerobiales	Halobacteroidaceae	Orenia	Orenia	sp.	
0	0	0	0	7	0	0	0	0	0	0	0	Bacteria	Halanaerobiaeota	Halanaerobiia	Halanaerobiales	Halanaerobiaceae				
0	0	0	0	0	0	19	0	0	0	0	0	Bacteria	Halanaerobiaeota	Halanaerobiia	Halanaerobiales	Halobacteroidaceae				
0	9	0	0	0	0	0	0	0	0	0	0	Bacteria	Halanaerobiaeota	Halanaerobiia	Halanaerobiales	Halobacteroidaceae				
0	0	66	0	0	0	218	0	67	51	0	198	Bacteria	Halanaerobiaeota	Halanaerobiia	Halanaerobiales	Halobacteroidaceae				
0	0	0	0	0	0	0	0	0	0	20	0	Bacteria	Halanaerobiaeota	Halanaerobiia	Halanaerobiales	Halobacteroidaceae				
0	0	228	0	0	0	69	0	0	0	0	0	Bacteria	Halanaerobiaeota	Halanaerobiia	Halanaerobiales	Halobacteroidaceae				
0	0	0	0	24	0	0	167	12	0	0	0	Archaea	Halobacterota	Halobacteria	Halobacterales	Haloferacaceae	A07HB70			
0	0	0	0	0	0	0	33	0	0	0	13	Archaea	Halobacterota	Halobacteria	Halobacterales	Haloferacaceae	A07HB70			
0	0	0	0	0	0	14	63	0	0	0	0	Archaea	Halobacterota	Halobacteria	Halobacterales	Haloferacaceae	A07HB70			
0	0	0	0	0	0	26	27	0	0	0	38	Archaea	Halobacterota	Halobacteria	Halobacterales	Halomicrobiaceae	Candidatus Halobonum	Candidatus Halobonum	sp.	
0	0	29	0	0	0	0	0	0	0	0	0	Archaea	Halobacterota	Halobacteria	Halobacterales	Haloadaptaceae	Haladaptatus	Haladaptatus	cibarius	D43
0	0	0	0	101	0	0	113	107	56	98	63	Archaea	Halobacterota	Halobacteria	Halobacterales	Haloadaptaceae	Haladaptatus	Halorussus	sp.	
0	0	0	0	0	60	0	0	0	0	0	0	Archaea	Halobacterota	Halobacteria	Halobacterales	Haloadaptaceae	Haladaptatus			
0	0	0	0	73	197	0	29	0	108	0	0	Archaea	Halobacterota	Halobacteria	Halobacterales	Halomicrobiaceae	Halapricum	Halapricum	sp.	
0	0	0	0	0	0	0	37	0	0	0	0	Archaea	Halobacterota	Halobacteria	Halobacterales	Halomicrobiaceae	Halapricum	Halapricum	sp.	
0	0	0	0	0	0	45	0	0	0	0	65	Archaea	Halobacterota	Halobacteria	Halobacterales	Halomicrobiaceae	Halapricum	Halapricum	sp.	
0	0	0	0	0	0	24	0	96	0	0	50	Archaea	Halobacterota	Halobacteria	Halobacterales	Halobacteriaceae	Halarchaeum	Halarchaeum	sp.	
0	0	0	0	0	0	0	0	0	33	0	0	Archaea	Halobacterota	Halobacteria	Halobacterales	Haloferacaceae	Haloarchaeobius	Haloarchaeobius	sp.	
0	0	95	0	0	0	0	28	0	0	0	56	Archaea	Halobacterota	Halobacteria	Halobacterales	Haloferacaceae	Haloarchaeobius	Haloarchaeobius	sp.	
0	0	0	0	0	0	0	0	0	42	0	0	Archaea	Halobacterota	Halobacteria	Halobacterales	Haloferacaceae	Haloarchaeobius	Haloarchaeobius	sp.	
0	0	0	0	0	0	0	0	0	0	0	14	Archaea	Halobacterota	Halobacteria	Halobacterales	Halococcaceae	Halococcus	Halococcus	sp.	
0	0	0	0	0	0	0	31	0	0	0	0	Archaea	Halobacterota	Halobacteria	Halobacterales	Halococcaceae	Halococcus	Halococcus	sp.	
0	23	0	0	33	0	0	0	0	0	0	0	Archaea	Halobacterota	Halobacteria	Halobacterales	Haloferacaceae	Haloferax	Haloferax	sp.	
0	86	73	0	0	0	0	0	24	0	0	0	Archaea	Halobacterota	Halobacteria	Halobacterales	Haloferacaceae	Haloferax	Haloferax	sp.	SB29
0	0	251	0	0	0	0	0	0	119	0	0	Archaea	Halobacterota	Halobacteria	Halobacterales	Haloferacaceae	Halogranum	Halogranum	sp.	
0	0	34	0	0	0	0	0	0	0	0	0	Archaea	Halobacterota	Halobacteria	Halobacterales	Haloferacaceae	Halolamina	Halolamina	sp.	
0	0	56	0	0	0	0	0	0	0	0	0	Archaea	Halobacterota	Halobacteria	Halobacterales	Halomicrobiaceae	Halomarina	Halomarina	sp.	
0	0	161	0	31	46	36	50	36	64	91	20	Archaea	Halobacterota	Halobacteria	Halobacterales	Halomicrobiaceae	Halomarina	Halomarina	oriensis	
0	0	20	0	0	0	0	0	0	0	0	0	Archaea	Halobacterota	Halobacteria	Halobacterales	Halomicrobiaceae	Halomarina	Halomarina	rubra	
0	0	0	0	66	78	46	0	25	41	0	56	Archaea	Halobacterota	Halobacteria	Halobacterales	Halomicrobiaceae	Halomarina	Halomarina	sp.	
0	0	0	0	0	0	60	0	0	20	0	0	Archaea	Halobacterota	Halobacteria	Halobacterales	Halomicrobiaceae	Halomicroarcula	Halomicroarcula	sp.	
0	0	0	0	28	64	0	0	70	25	44	0	Archaea	Halobacterota	Halobacteria	Halobacterales	Halomicrobiaceae	Halomicroarcula	Halomicroarcula	sp.	YJ-61-S
0	0	0	0	0	0	0	0	0	0	25	0	Archaea	Halobacterota	Halobacteria	Halobacterales	Halomicrobiaceae	Halomicroarcula	Halomicroarcula	sp.	
0	0	0	0	0	0	33	0	0	80	0	0	Archaea	Halobacterota	Halobacteria	Halobacterales	Halomicrobiaceae	Halomicroarcula	Halomicroarcula	sp.	
0	0	0	0	0	0	0	0	0	40	91	0	Archaea	Halobacterota	Halobacteria	Halobacterales	Halomicrobiaceae	Halomicrobium	Halomicrobium	sp.	
0	0	0	0	0	75	24	62	0	0	49	0	Archaea	Halobacterota	Halobacteria	Halobacterales	Halomicrobiaceae	Halomicrobium	Halomicrobium	sp.	
0	0	0	0	0	0	0	0	0	0	0	11	Archaea	Halobacterota	Halobacteria	Halobacterales	Halomicrobiaceae	Halomicrobium	Halomicrobium	zhouii	
0	0	0	0	0	0	0	0	58	0	0	28	Archaea	Halobacterota	Halobacteria	Halobacterales	Halomicrobiaceae	Halomicrobium	Halomicrobium	sp.	
0	0	0	0	0	0	0	0	0	0	0	35	Archaea	Halobacterota	Halobacteria	Halobacterales	Haloferacaceae	Haloplanus	Haloplanus	sp.	
0	0	0	0	25	0	0	0	0	0	0	0	Archaea	Halobacterota	Halobacteria	Halobacterales	Haloferacaceae	Haloplanus	Haloplanus	sp.	
0	0	0	0	0	29	0	35	58	0	42	57	Archaea	Halobacterota	Halobacteria	Halobacterales	Haloferacaceae	Haloplanus	Haloplanus	sp.	LZ-23S87
0	0	0	0	0	0	0	0	0	31	0	0	Archaea	Halobacterota	Halobacteria	Halobacterales	Haloferacaceae	Haloplanus	Haloplanus	sp.	
0	0	0	0	0	24	0	0	0	0	0	0	Archaea	Halobacterota	Halobacteria	Halobacterales	Halomicrobiaceae	Halorientalis	Halorientalis	sp.	
0	0	0	0	0	0	0	0	0	18	0	0	Archaea	Halobacterota	Halobacteria	Halobacterales	Halomicrobiaceae	Halorientalis	Halorientalis	sp.	TGN-40
0	0	0	0	0	0	0	0	0	41	0	0	Archaea	Halobacterota	Halobacteria	Halobacterales	Halomicrobiaceae	Halorientalis	Halorientalis	persicus	
0	0	0	0	0	0	29	0	0	0	0	40	Archaea	Halobacterota	Halobacteria	Halobacterales	Halomicrobiaceae	Halorientalis	Halorientalis	sp.	
0	0	0	0	0	0	0	47	0	0	33	40	Archaea	Halobacterota	Halobacteria	Halobacterales	Haloferacaceae	Halorubellus	Halorubellus	sp.	
0	0	0	0	0	81	0	95	27	0	22	3	Archaea	Halobacterota	Halobacteria	Halobacterales	Haloferacaceae	Halorubellus	Halorubellus	sp.	
0	0	0	0	0	0	0	0	20	0	0	0	Archaea	Halobacterota	Halobacteria	Halobacterales	Haloferacaceae	Halorubellus	Halorubellus	sp.	
0	0	0	0	0	0	0	0	0	17	0	35	Archaea	Halobacterota	Halobacteria	Halobacterales	Haloferacaceae	Halorussus	Halorussus	sp.	
0	0	0	0	0	0	121	65	0	0	0	153	Archaea	Halobacterota	Halobacteria	Halobacterales	Haloferacaceae	Halorussus	Halorussus	sp.	
0	0	92	0	64	11	19	0	0	125	38	0	Archaea	Halobacterota	Halobacteria	Halobacterales	Haloferacaceae	Halorussus	Halorussus	sp.	ZS-3
0	0	0	0	0	0	0	0	64	53	20	0	Archaea	Halobacterota	Halobacteria	Halobacterales	Haloferacaceae	Halorussus	Halorussus	salinus	
0	0	0	0	0	0	21	28	0	67	40	91	Archaea	Halobacterota	Halobacteria	Halobacterales	Haloferacaceae	Halorussus	Halorussus	sp.	
0	0	0	0	0	47	0	0	0	0	0	0	Archaea	Halobacterota	Halobacteria	Halobacterales	Haloferacaceae	Halorussus	Halorussus	sp.	
0	0	0	0	0	0	0	31	0	0	0	0	Archaea	Halobacterota	Halobacteria	Halobacterales	Halomicrobiaceae	Halosimplex	Halosimplex	sp.	
0	0	0	0	0	0	0	0	67	0	46	0	Archaea	Halobacterota	Halobacteria	Halobacterales	Halococcaceae	Halostella	Halostella	sp.	
0	0	0	0	0	0	0	13	38	0	18	0	Archaea	Halobacterota	Halobacteria	Halobacterales	Haloferacaceae	Halovenus	Halovenus	sp.	
0	0	0	0	0	0	0	20	27	0	59	0	Archaea	Halobacterota	Halobacteria	Halobacterales	Haloferacaceae	Natronoarchaeum	Natronoarchaeum	sp.	
0	0	0	0	52	56	97	71	52	62	44	39	Archaea	Halobacterota	Halobacteria	Halobacterales	Halomicrobiaceae	Natronomonas	Natronomonas	sp.	
0	19	19	0	0	0	0	68	47	0	39	0	Archaea	Halobacterota	Halobacteria	Halobacterales	Halomicrobiaceae	Natronomonas	Natronomonas	sp.	
0	0	0	0	0	28	0	0	0	0	0	0	Archaea	Halobacterota	Halobacteria	Halobacterales	Haloferacaceae	Salinarchaeum	Salinarchaeum	sp.	
0	0	0	0	0	11	0	0	0	0	16	0	Archaea	Halobacterota	Halobacteria	Halobacterales	Haloferacaceae	Salinarchaeum	Salinarchaeum	sp.	Harcht-Bsk1
0	0	0	0	0	0	0	60	0	0	0	0	Archaea	Halobacterota	Halobacteria	Halobacterales	Haloferacaceae	Salinarchaeum	Salinarchaeum	sp.	
0	0	0	0	0	0	0	55	0	0	0	0	Archaea	Halobacterota	Halobacteria	Halobacterales	Haloferacaceae	Salinigranum	Salinigranum	sp.	
0	0	0	0	0	0	48	26	55	0	0	25	Archaea	Halobacterota	Halobacteria	Halobacterales	Haloferacaceae	Salinigranum	Salinigranum	sp.	
0	0	0	0	0	0	0	44	15	13	59	0	Archaea	Halobacterota	Halobacteria	Halobacterales	Halomicrobiaceae	Salinirubrum	Salinirubrum	sp.	
0	0	0	0	0	32	0	0	0	0	0	0	Archaea	Halobacterota	Halobacteria	Halobacterales	Halomicrobiaceae	Salinirubrum	Salinirubrum	sp.	ZS-13-S
0	0	0	0	113	175	0	247	279	182	384	65	Archaea	Halobacterota	Halobacteria	Halobacterales	Halomicrobiaceae	Salinirubrum	Salinirubrum	sp.	
0	0	0	0	0	32	0	0	0	0	0	0	Archaea	Halobacterota	Halobacteria	Halobacterales	Haloferacaceae				
0	0	0	0	0	8	0	64	17	0	0	13	Archaea	Halobacterota	Halobacteria	Halobacterales	Haloferacaceae				
0	0	0	0	83	22	0	0	0	0	0	0	Archaea	Halobacterota	Halobacteria	Halobacterales	Haloferacaceae				
0	0	0	0	138	99	21	65	159	135	0	38	Archaea	Halobacterota	Halobacteria	Halobacterales	Halomicrobiaceae				
0	0	0	0	33	0	0	0	0	62	0	0	Archaea	Halobacterota	Halobacteria	Halobacterales	Halomicrobiaceae				
0	0	0	0	20	0	0	0	0	0	48	73	Archaea	Halobacterota	Halobacteria	Halobacterales	Halomicrobiaceae				
0	0	0	0	62	0	0	0	0	0	0	40	Archaea	Halobacterota	Halobacteria	Halobacterales	Halomicrobiaceae				
0	0	0	0	40	0	0	0	0	0	0	0	Archaea	Halobacterota	Halobacteria	Halobacterales	Halomicrobiaceae				
0	0	0	0	0	0	0	0	0	0	26	0	Archaea	Halobacterota	Halobacteria	Halobacterales	Halomicrobiaceae				
0	0	0	0	94	42	0	45	72	0	132	20	Archaea	Halobacterota	Halobacteria	Halobacterales	Halomicrobiaceae				
0	0	0	0	0	0	63	0	0	0	0	0	Archaea	Halobacterota	Halobacteria	Halobacterales	unclassified_Archaea				
0	0	0	0	108	117	63	196	282	125	184	223	Archaea	Halobacterota	Halobacteria	Halobacterales	unclassified_Archaea				
0	0	0	0	0	0	0	0	0	41	0	0	Archaea	Halobacterota	Halobacteria	Halobacterales	unclassified_Archaea				
0	0	0	0	0	32	150	70	0	0	0	72	Archaea	Halobacterota	Halobacteria	Halobacterales	unclassified_Archaea		Halorussus	sp.	HD8-83
0	0	0	0	0	0	0	27	0	0	0	0	Archaea	Halobacterota	Halobacteria	Halobacterales	unclassified_Archaea				
0	0	0	0	0	0	0	56	0	0	0	0	Archaea	Halobacterota	Halobacteria	Halobacterales	unclassified_Archaea				
0	0	0	0	0	0	0	0	0	0	0	17	Archaea	Halobacterota	unclassified_Archaea	unclassified_Archaea	unclassified_Archaea				
0	0	0	0	0	0	8	0	0	0	0	0	Bacteria	Hydrogenedentes	Hydrogenedentia	Hydrogenedentiales	Hydrogenedensaceae				
0	0	0	0	0	0	0	15	36	0	12	0	Bacteria	Hydrogenedentes	Hydrogenedentia	Hydrogenedentiales	Hydrogenedensaceae				
0	0	0	0	0	33	0	38	15	0	0	21	Bacteria	Hydrogenedentes	Hydrogenedentia	Hydrogenedentiales	Hydrogenedensaceae				
0	0	0	0	0	0	0	0	11	0	0	0	Bacteria	Latescibacterota	Latescibacterota	Latescibacterota	Latescibacterota	Acidobacterium	Acidobacterium	sp.	
0	0	0	0	0	29	0	0	0	0	0	8	Bacteria	Latescibacterota	Latescibacteria	Latescibacterales	Latescibacteraceae	Candidatus Latescibacter			
0	0	0	0	0	0	0	0	11	0	0	0	Bacteria	Latescibacterota	Latescibacteria	Latescibacterales	Latescibacteraceae				
0	0	0	0	0	9	0	0	4	0	0	23	Bacteria	Latescibacterota	Latescibacterota	Latescibacterota	Latescibacterota				
0	0	0	0	61	19	0	0	0	47	7	27	Bacteria	Latescibacterota	Latescibacterota	Latescibacterota	Latescibacterota				
0	0	0	0	0	0	0	0	0	4	0	0	Bacteria	Latescibacterota	Latescibacterota	Latescibacterota	Latescibacterota				
0	0	0	9	0	0	0	0	0	16	0	0	Bacteria	LCP-89	unclassified_Bacteria	unclassified_Bacteria	unclassified_Bacteria				
0	0	0	29	0	0	0	0	0	0	0	0	Bacteria	LCP-89	unclassified_Bacteria	unclassified_Bacteria	unclassified_Bacteria				
0	0	0	0	13	7	0	0	21	31	56	97	Bacteria	Marinimicrobia (SAR406 clade)	Marinimicrobia (SAR406 clade)	unclassified_Bacteria	unclassified_Bacteria				
0	0	0	27	0	0	0	0	45	0	0	0	Bacteria	Marinimicrobia (SAR406 clade)	Marinimicrobia (SAR406 clade)	unclassified_Bacteria	unclassified_Bacteria				
0	0	0	0	0	0	0	0	5	0	0	0	Bacteria	Melainabacteria	Vampirivibrionia	Caenarcaniphilales	unclassified_Bacteria				
0	0	0	0	13	22	0	0	0	0	0	0	Bacteria	Melainabacteria	Vampirivibrionia	Gastranaerophilales	unclassified_Bacteria				
0	0	0	0	0	44	0	0	34	43	0	0	Bacteria	Melainabacteria	Vampirivibrionia	Gastranaerophilales	unclassified_Bacteria				
0	0	0	0	0	0	0	0	30	0	0	23	Bacteria	Melainabacteria	Vampirivibrionia	Vampirovibrionales	Vampirovibrionaceae				
0	0	0	0	0	0	0	0	0	0	9	0	Bacteria	Melainabacteria	Vampirivibrionia	Vampirovibrionales	unclassified_Bacteria				
0	0	0	0	6	0	0	0	0	0	0	0	Bacteria	Melainabacteria	Vampirivibrionia	Vampirovibrionales	unclassified_Bacteria				
0	0	0	0	0	0	0	0	0	26	0	0	Bacteria	Modulibacteria	Moduliflexia	Moduliflexales	Moduliflexaceae				
0	0	0	0	0	0	11	0	0	0	0	0	Bacteria	Modulibacteria	Moduliflexia	Moduliflexales	Moduliflexaceae				
0	0	0	0	0	0	0	0	0	0	0	9	Bacteria	Modulibacteria	Moduliflexia	Moduliflexales	Moduliflexaceae				
0	0	0	0	0	0	0	0	28	0	0	0	Bacteria	Myxococcota	Polyangia	Polyangiales	unclassified_Bacteria	BIrii41			
61	0	0	0	0	0	0	0	0	0	0	0	Bacteria	Myxococcota	Polyangia	Polyangiales	unclassified_Bacteria	BIrii41			
16	0	0	0	29	19	58	63	33	50	29	13	Bacteria	Myxococcota	Polyangia	Polyangiales	unclassified_Bacteria	BIrii41			
0	0	0	0	0	0	0	20	0	0	0	0	Bacteria	Myxococcota	Polyangia	Polyangiales	unclassified_Bacteria	Eel-36e1D6			
0	0	0	0	0	0	58	0	0	0	0	0	Bacteria	Myxococcota	Polyangia	Haliangiales	Haliangiaceae	Haliangium	Haliangium	sp.	
0	0	0	0	0	0	0	16	0	0	0	0	Bacteria	Myxococcota	Polyangia	Haliangiales	Haliangiaceae	Haliangium	Haliangium	tepidum	
0	0	0	0	0	34	0	0	0	0	0	0	Bacteria	Myxococcota	Polyangia	unclassified_Bacteria	unclassified_Bacteria	MidBa8			
0	0	0	0	42	34	0	0	0	0	0	0	Bacteria	Myxococcota	Polyangia	unclassified_Bacteria	unclassified_Bacteria	MidBa8			
0	0	0	0	0	0	0	0	0	18	0	0	Bacteria	Myxococcota	Polyangia	unclassified_Bacteria	unclassified_Bacteria	MidBa8			
47	0	0	0	0	0	30	0	0	0	0	0	Bacteria	Myxococcota	Polyangia	Nannocystales	Nannocystaceae	Nannocystis	Nannocystis	sp.	
0	0	0	0	48	33	0	0	0	0	0	0	Bacteria	Myxococcota	Polyangia	Polyangiales	Phaselicystidaceae	Phaselicystis	Phaselicystis	sp.	
0	0	0	0	0	0	0	0	0	0	0	18	Bacteria	Myxococcota	Polyangia	Polyangiales	Sandaracinaceae	Sandaracinus	Sandaracinus	sp.	
0	0	0	15	0	0	0	0	0	0	0	0	Bacteria	Myxococcota	Polyangia	Polyangiales	Sandaracinaceae	Sandaracinus	Sandaracinus	sp.	
0	0	0	0	0	0	35	0	0	21	0	0	Bacteria	Myxococcota	Polyangia	unclassified_Bacteria	unclassified_Bacteria	UASB-TL25			
0	0	0	33	0	0	0	0	18	0	0	0	Bacteria	Myxococcota	Polyangia	unclassified_Bacteria	unclassified_Bacteria	UASB-TL25			
0	0	0	0	18	11	0	0	33	0	4	0	Bacteria	Myxococcota	Polyangia	unclassified_Bacteria	unclassified_Bacteria	VHS-B3-70			
0	0	0	0	0	0	0	0	0	0	18	0	Bacteria	Myxococcota	Polyangia	unclassified_Bacteria	unclassified_Bacteria	VHS-B4-70			
0	0	0	0	0	0	25	0	0	0	13	0	Bacteria	Myxococcota	Myxococcia	Myxococcales	Myxococcaceae				
0	34	0	0	86	110	0	89	0	0	0	0	Bacteria	Myxococcota	Myxococcia	Myxococcales	Myxococcaceae				P3OB-42
0	29	0	0	5	0	38	4	115	67	94	138	Bacteria	Myxococcota	Myxococcia	Myxococcales	Myxococcaceae				P3OB-42
0	0	0	34	70	63	0	0	56	0	0	0	Bacteria	Myxococcota	Myxococcia	Myxococcales	Myxococcaceae				P3OB-42
0	0	0	0	0	0	6	0	0	0	0	0	Bacteria	Myxococcota	Myxococcia	Myxococcales	Myxococcaceae				P3OB-42
0	0	0	0	0	0	0	0	0	0	31	0	Bacteria	Myxococcota	Myxococcia	Myxococcales	Myxococcaceae				P3OB-42
0	0	0	0	0	0	22	0	0	0	0	0	Bacteria	Myxococcota	Myxococcia	Myxococcales	Myxococcaceae				P3OB-42
0	0	0	0	0	0	0	130	55	51	0	70	Bacteria	Myxococcota	Myxococcia	Myxococcales	Myxococcaceae				P3OB-42
0	0	0	0	24	0	0	0	41	18	14	19	Bacteria	Myxococcota	Polyangia	Nannocystales	Nannocystaceae				
0	0	0	0	0	0	0	0	0	0	0	38	Bacteria	Myxococcota	Polyangia	Nannocystales	Nannocystaceae				
0	0	0	0	9	0	0	0	15	0	0	0	Bacteria	Myxococcota	Polyangia	Nannocystales	Nannocystaceae				
106	66	0	0	34	0	25	35	0	0	46	0	Bacteria	Myxococcota	Polyangia	Polyangiales	Sandaracinaceae				
13	0	0	0	0	0	16	32	16	0	13	0	Bacteria	Myxococcota	Polyangia	Polyangiales	Sandaracinaceae				
0	0	0	0	0	0	48	0	0	0	0	0	Bacteria	Myxococcota	Polyangia	Polyangiales	Sandaracinaceae				
9	0	0	0	0	0	0	0	0	0	0	0	Bacteria	Myxococcota	Polyangia	Polyangiales	Sandaracinaceae				
0	0	0	0	60	38	0	22	52	0	0	66	Bacteria	Myxococcota	Polyangia	Polyangiales	Sandaracinaceae				
0	0	0	0	6	0	38	0	0	0	0	0	Bacteria	Myxococcota	Polyangia	unclassified_Bacteria	unclassified_Bacteria				

0	0	0	0	0	0	0	0	0	0	0	9	Archaea	Nanoarchaeota	Nanoarchaeia	Woesearchaeales	unclassified_Archaea	GW2011			AR15
0	0	0	3	0	0	0	0	0	0	0	0	Archaea	Nanoarchaeota	Nanoarchaeia	Woesearchaeales	unclassified_Archaea	SCGC AAA286-E23			
0	0	0	5	0	0	0	0	39	23	0	20	Archaea	Nanoarchaeota	Nanoarchaeia	Woesearchaeales	unclassified_Archaea				
10	26	0	25	28	0	0	27	0	0	78	27	Archaea	Nanoarchaeota	Nanoarchaeia	Woesearchaeales	unclassified_Archaea				
0	0	0	0	0	21	0	0	0	0	0	0	Archaea	Nanoarchaeota	Nanoarchaeia	Woesearchaeales	unclassified_Archaea				
0	0	0	0	0	43	0	0	0	12	0	0	Archaea	Nanohaloarchaeota	Nanosalinia	Nanosalinales	Nanosalinaceae	Candidatus Haloredivivus	Candidatus Haloredivivus	sp.	G17
0	0	0	0	0	26	0	0	0	0	0	0	Archaea	Nanohaloarchaeota	Nanosalinia	Nanosalinales	Nanosalinaceae				
0	0	0	0	0	0	0	0	0	0	7	0	Archaea	Nanohaloarchaeota	Nanosalinia	Nanosalinales	Nanosalinaceae				
0	0	0	0	0	16	0	13	28	55	0	23	Archaea	Nanohaloarchaeota	Nanosalinia	Nanosalinales	Nanosalinaceae				
0	0	10	0	29	0	0	0	94	161	72	36	Archaea	Nanohaloarchaeota	Nanosalinia	Nanosalinales	Nanosalinaceae				
0	0	0	0	0	0	0	70	0	0	0	0	Bacteria	NB1-j	unclassified_Bacteria	unclassified_Bacteria	unclassified_Bacteria				
0	0	0	0	0	0	6	0	0	0	0	0	Bacteria	NB1-j	unclassified_Bacteria	unclassified_Bacteria	unclassified_Bacteria				
0	0	0	0	0	0	0	0	7	0	0	0	Bacteria	Nitrospirota	Nitrospiria	Nitrospirales	Nitrospiraceae	Nitrospira	Nitrospira	sp.	clone-Gall
0	0	0	16	0	0	0	0	0	0	0	0	Bacteria	Patescibacteria	Gracilibacteria	unclassified_Bacteria	unclassified_Bacteria	JGI0000069-P22			
0	0	15	0	0	0	0	0	0	0	0	0	Bacteria	Patescibacteria	Gracilibacteria	unclassified_Bacteria	unclassified_Bacteria	JGI0000069-P22			RFB-d08
0	0	0	12	0	0	0	0	0	0	0	0	Bacteria	Patescibacteria	Gracilibacteria	unclassified_Bacteria	unclassified_Bacteria	JGI0000069-P22			
0	0	57	0	0	0	0	0	0	0	0	0	Bacteria	Patescibacteria	Gracilibacteria	unclassified_Bacteria	unclassified_Bacteria	JGI0000069-P22			
0	0	0	4	0	0	0	0	0	0	0	0	Bacteria	Patescibacteria	Gracilibacteria	Absconditabacteriales (SR1)	unclassified_Bacteria				
0	0	0	0	0	0	0	0	0	0	0	5	Bacteria	Patescibacteria	Microgenomatia	Candidatus Chisholmbacteria	unclassified_Bacteria				
0	0	0	16	0	0	0	0	0	11	0	0	Bacteria	Patescibacteria	Parcubacteria	Candidatus Kaiserbacteria	unclassified_Bacteria				
0	0	0	0	0	0	0	0	0	9	0	0	Bacteria	Patescibacteria	Gracilibacteria	Candidatus Peregrinibacteria	unclassified_Bacteria				
0	0	0	0	0	0	9	0	0	0	0	0	Bacteria	Patescibacteria	Parcubacteria	Candidatus Terrybacteria	unclassified_Bacteria				
0	0	0	0	0	0	44	0	0	0	0	0	Bacteria	Patescibacteria	Microgenomatia	Candidatus Woesebacteria	unclassified_Bacteria				
0	0	0	0	0	0	0	0	29	0	0	11	Bacteria	Patescibacteria	Gracilibacteria	unclassified_Bacteria	unclassified_Bacteria				
0	9	0	9	0	0	0	0	0	0	0	0	Bacteria	Patescibacteria	Gracilibacteria	unclassified_Bacteria	unclassified_Bacteria				
0	0	0	0	0	0	0	0	0	0	0	6	Bacteria	Patescibacteria	Gracilibacteria	unclassified_Bacteria	unclassified_Bacteria				
0	0	0	0	0	0	9	0	0	0	5	0	Bacteria	Patescibacteria	Parcubacteria	unclassified_Bacteria	unclassified_Bacteria				
0	0	0	0	0	0	10	0	0	0	0	0	Bacteria	PAUC34f	unclassified_Bacteria	unclassified_Bacteria	unclassified_Bacteria				
0	0	0	2	0	0	0	0	0	0	0	0	Bacteria	PAUC34f	unclassified_Bacteria	unclassified_Bacteria	unclassified_Bacteria				
0	0	0	0	0	0	0	6	0	0	0	0	Bacteria	PAUC34f	unclassified_Bacteria	unclassified_Bacteria	unclassified_Bacteria				
0	0	0	0	6	0	0	0	0	0	0	0	Bacteria	PAUC34f	unclassified_Bacteria	unclassified_Bacteria	unclassified_Bacteria				
0	0	0	0	0	0	15	0	0	0	0	0	Bacteria	PAUC34f	unclassified_Bacteria	unclassified_Bacteria	unclassified_Bacteria				
0	0	0	0	0	0	0	0	0	0	0	8	Bacteria	Planctomycetota	Phycisphaerae	Phycisphaerales	unclassified_Bacteria	AKAU3564 sediment group			
0	0	0	0	0	0	0	0	0	40	0	0	Bacteria	Planctomycetota	Phycisphaerae	Phycisphaerales	unclassified_Bacteria	AKAU3564 sediment group			
0	0	0	0	274	220	0	0	104	164	36	67	Bacteria	Planctomycetota	Phycisphaerae	Phycisphaerales	unclassified_Bacteria	AKAU3564 sediment group			
0	0	0	8	0	0	0	0	0	0	0	0	Bacteria	Planctomycetota	unclassified_Bacteria	unclassified_Bacteria	unclassified_Bacteria	BD7-11			
0	0	0	0	0	0	0	47	0	0	0	0	Bacteria	Planctomycetota	Planctomycetes	Pirellulales	Pirellulaceae	Blastopirellula	Blastopirellula	sp.	
0	0	0	0	15	0	0	0	0	0	0	0	Bacteria	Planctomycetota	Planctomycetes	Pirellulales	Pirellulaceae	Blastopirellula	Blastopirellula	sp.	
0	0	0	0	8	14	0	0	0	0	0	0	Bacteria	Planctomycetota	Planctomycetes	Pirellulales	Pirellulaceae	Anammoximicrobium	Anammoximicrobium	sp.	
0	0	0	0	50	0	0	0	27	0	18	40	Bacteria	Planctomycetota	Phycisphaerae	unclassified_Bacteria	unclassified_Bacteria	CCM11a			
0	0	0	0	11	0	0	31	0	0	0	0	Bacteria	Planctomycetota	Phycisphaerae	unclassified_Bacteria	unclassified_Bacteria	CCM11a			
0	0	0	0	0	0	0	0	0	0	0	68	Bacteria	Planctomycetota	Phycisphaerae	MSBL9	unclassified_Bacteria	KCLunmb-38-53			
0	0	0	0	0	14	0	0	0	0	0	0	Bacteria	Planctomycetota	Phycisphaerae	MSBL9	unclassified_Bacteria	KCLunmb-38-53			
0	0	0	0	45	51	0	0	0	0	0	0	Bacteria	Planctomycetota	Phycisphaerae	MSBL9	unclassified_Bacteria	KCLunmb-38-53			
0	0	0	0	0	0	0	6	0	0	0	0	Bacteria	Planctomycetota	unclassified_Bacteria	unclassified_Bacteria	unclassified_Bacteria	OM190			
0	0	0	0	0	0	0	0	0	0	0	2	Bacteria	Planctomycetota	unclassified_Bacteria	unclassified_Bacteria	unclassified_Bacteria	OM190			
0	0	0	0	0	12	0	11	19	0	0	0	Bacteria	Planctomycetota	unclassified_Bacteria	unclassified_Bacteria	unclassified_Bacteria	OM190			
18	0	0	0	0	0	0	0	0	0	0	0	Bacteria	Planctomycetota	Phycisphaerae	Phycisphaerales	Phycisphaeraceae	Phycisphaera	Phycisphaera	sp.	
0	0	0	0	0	0	20	23	0	0	0	0	Bacteria	Planctomycetota	Planctomycetes	Pirellulales	Pirellulaceae	Pir4 lineage			
0	0	0	0	0	0	0	24	0	0	0	0	Bacteria	Planctomycetota	Planctomycetes	Pirellulales	Pirellulaceae	Pir4 lineage			
0	33	0	0	0	0	0	15	16	0	0	17	Bacteria	Planctomycetota	Planctomycetes	Pirellulales	Pirellulaceae	Pirellula	Pirellula	sp.	
0	0	0	0	0	0	9	14	0	0	0	0	Bacteria	Planctomycetota	unclassified_Bacteria	unclassified_Bacteria	unclassified_Bacteria	Pla3 lineage			
0	0	0	0	10	0	9	0	0	0	0	0	Bacteria	Planctomycetota	unclassified_Bacteria	unclassified_Bacteria	unclassified_Bacteria	Pla3 lineage			
0	0	0	0	0	0	0	0	0	0	0	15	Bacteria	Planctomycetota	unclassified_Bacteria	unclassified_Bacteria	unclassified_Bacteria	Pla3 lineage			
0	0	0	0	0	0	16	0	23	0	0	0	Bacteria	Planctomycetota	unclassified_Bacteria	unclassified_Bacteria	unclassified_Bacteria	Pla4 lineage			
0	0	0	0	0	0	0	0	0	0	0	26	Bacteria	Planctomycetota	unclassified_Bacteria	unclassified_Bacteria	unclassified_Bacteria	Pla4 lineage			
0	0	0	21	0	0	70	0	0	0	0	0	Bacteria	Planctomycetota	Planctomycetes	Pirellulales	Pirellulaceae	Rhodopirellula	Rhodopirellula	sp.	
0	0	0	0	0	0	0	0	0	0	0	13	Bacteria	Planctomycetota	Planctomycetes	Pirellulales	Pirellulaceae	Rhodopirellula	Rhodopirellula	sp.	WH5-6
0	0	0	0	0	0	0	40	0	0	0	0	Bacteria	Planctomycetota	Planctomycetes	Pirellulales	Pirellulaceae	Rhodopirellula	Rhodopirellula	sp.	
0	0	0	15	0	0	0	0	37	32	0	0	Bacteria	Planctomycetota	Planctomycetes	Pirellulales	Pirellulaceae	Rhodopirellula	Rhodopirellula	sp.	
0	0	0	0	0	0	0	0	0	0	6	0	Bacteria	Planctomycetota	Planctomycetes	Planctomycetales	Rubinisphaeraceae	Rubinisphaera	Rubinisphaera	sp.	
0	0	0	0	0	0	0	19	0	0	0	6	Bacteria	Planctomycetota	Planctomycetes	Planctomycetales	Rubinisphaeraceae	Rubinisphaera	Rubinisphaera	sp.	
28	39	12	0	0	0	38	0	0	0	0	0	Bacteria	Planctomycetota	Planctomycetes	Pirellulales	Pirellulaceae	Rubripirellula	Rubripirellula	sp.	
0	0	0	0	17	14	0	0	0	0	0	9	Bacteria	Planctomycetota	Phycisphaerae	MSBL9	unclassified_Bacteria	SG8-4			
0	0	0	0	0	0	4	0	0	0	0	0	Bacteria	Planctomycetota	Phycisphaerae	MSBL9	unclassified_Bacteria	SG8-4			
0	0	0	0	36	0	0	51	6	0	0	0	Bacteria	Planctomycetota	Phycisphaerae	Phycisphaerales	Phycisphaeraceae	SM1A02			
0	0	0	0	0	0	0	29	0	0	0	0	Bacteria	Planctomycetota	Phycisphaerae	Phycisphaerales	Phycisphaeraceae	SM1A02			
0	0	0	17	0	0	0	0	30	0	26	0	Bacteria	Planctomycetota	Phycisphaerae	Phycisphaerales	Phycisphaeraceae	SM1A02			
0	0	0	0	12	0	0	0	0	0	0	0	Bacteria	Planctomycetota	Phycisphaerae	MSBL9	unclassified_Bacteria	SM23-30			
0	0	0	0	0	59	0	0	8	18	0	24	Bacteria	Planctomycetota	Brocadiae	unclassified_Bacteria	unclassified_Bacteria	SM23-32			
0	0	0	0	61	61	0	0	23	27	19	0	Bacteria	Planctomycetota	Phycisphaerae	MSBL9	unclassified_Bacteria	ST-NAGAB-D1			
0	0	0	0	0	0	0	6	0	0	0	0	Bacteria	Planctomycetota	Phycisphaerae	Phycisphaerales	Phycisphaeraceae	Urania-1B-19 marine sediment group			
0	0	0	0	15	0	0	0	0	0	0	0	Bacteria	Planctomycetota	Phycisphaerae	Phycisphaerales	Phycisphaeraceae	Urania-1B-19 marine sediment group			
0	29	0	0	89	42	14	89	0	0	19	8	Bacteria	Planctomycetota	unclassified_Bacteria	unclassified_Bacteria	unclassified_Bacteria	vadinHA49			
0	0	0	13	0	0	0	0	0	0	0	17	Bacteria	Planctomycetota	unclassified_Bacteria	unclassified_Bacteria	unclassified_Bacteria	vadinHA49			
0	0	0	0	0	0	7	0	0	0	0	11	Bacteria	Planctomycetota	unclassified_Bacteria	unclassified_Bacteria	unclassified_Bacteria	vadinHA49			
0	0	0	0	0	0	10	0	0	0	0	18	Bacteria	Planctomycetota	unclassified_Bacteria	unclassified_Bacteria	unclassified_Bacteria	vadinHA49			
0	0	0	0	58	25	0	0	11	0	0	28	Bacteria	Planctomycetota	unclassified_Bacteria	unclassified_Bacteria	unclassified_Bacteria	vadinHA49			
0	20	0	16	93	140	0	84	80	166	0	40	Bacteria	Planctomycetota	unclassified_Bacteria	unclassified_Bacteria	unclassified_Bacteria	vadinHA49			
0	0	0	0	0	14	0	0	0	0	0	0	Bacteria	Planctomycetota	unclassified_Bacteria	unclassified_Bacteria	unclassified_Bacteria	vadinHA49			
0	0	0	0	0	0	0	57	11	0	0	15	Bacteria	Planctomycetota	Planctomycetes	Gemmatales	Gemmataceae				
0	0	0	0	20	0	0	65	90	0	21	103	Bacteria	Planctomycetota	Phycisphaerae	mle1-8	unclassified_Bacteria				
0	0	0	0	24	0	0	0	0	26	0	26	Bacteria	Planctomycetota	Phycisphaerae	mle1-8	unclassified_Bacteria				
0	0	0	0	0	46	0	11	0	0	32	9	Bacteria	Planctomycetota	Phycisphaerae	mle1-8	unclassified_Bacteria				
0	0	0	0	28	17	0	21	12	2	0	0	Bacteria	Planctomycetota	Phycisphaerae	Phycisphaerales	Phycisphaeraceae				
0	0	0	0	0	0	0	38	42	17	17	29	Bacteria	Planctomycetota	Phycisphaerae	Phycisphaerales	Phycisphaeraceae				
0	6	0	9	122	39	0	62	70	29	37	83	Bacteria	Planctomycetota	Phycisphaerae	Phycisphaerales	Phycisphaeraceae				
0	0	0	0	20	0	0	15	96	18	31	0	Bacteria	Planctomycetota	Phycisphaerae	Phycisphaerales	Phycisphaeraceae				
0	0	0	0	28	13	0	63	14	0	17	0	Bacteria	Planctomycetota	Planctomycetes	Pirellulales	Pirellulaceae				
0	48	0	0	0	0	0	43	0	0	0	0	Bacteria	Planctomycetota	Planctomycetes	Pirellulales	Pirellulaceae				
0	0	0	0	0	0	0	6	35	27	0	0	Bacteria	Planctomycetota	Planctomycetes	Pirellulales	Pirellulaceae				
14	0	0	0	0	0	0	0	0	0	0	0	Bacteria	Planctomycetota	Planctomycetes	Planctomycetales	Gimesiaceae				
0	0	0	0	0	0	0	18	0	0	0	0	Bacteria	Planctomycetota	Planctomycetes	Planctomycetales	Gimesiaceae				
0	0	0	0	0	0	0	22	0	0	0	0	Bacteria	Planctomycetota	Planctomycetes	Planctomycetales	unclassified_Bacteria				
0	0	0	0	9	0	0	31	14	0	0	15	Bacteria	Planctomycetota	Planctomycetes	Planctomycetales	unclassified_Bacteria				DDD3G8
0	0	0	0	0	0	21	0	0	0	0	0	Bacteria	Planctomycetota	Planctomycetes	Planctomycetales	unclassified_Bacteria				
0	50	0	0	0	0	0	0	0	0	0	0	Bacteria	Proteobacteria	Gammaproteobacteria	Ectothiorhodospirales	Acidihalobacteraceae	Acidihalobacter	Acidihalobacter	sp.	
0	12	0	0	0	0	0	0	0	0	0	0	Bacteria	Proteobacteria	Alphaproteobacteria	Rhodobacterales	Rhodobacteraceae	Actibacterium	Actibacterium		
0	19	0	0	0	0	0	0	0	0	0	0	Bacteria	Proteobacteria	Alphaproteobacteria	Rhodobacterales	Rhodobacteraceae	Albimonas	Albimonas	pacifica	
0	108	0	0	0	0	0	0	0	0	0	0	Bacteria	Proteobacteria	Gammaproteobacteria	Oceanospirillales	Alcanivoracaceae1	Alcanivorax	Alcanivorax	sp.	
43	0	0	55	0	0	0	0	0	0	0	0	Bacteria	Proteobacteria	Gammaproteobacteria	Oceanospirillales	Alcanivoracaceae1	Alcanivorax	Alcanivorax	sp.	
0	0	17	0	90	59	0	28	56	75	33	0	Bacteria	Proteobacteria	Gammaproteobacteria	Oceanospirillales	Alcanivoracaceae1	Alcanivorax	Alcanivorax	sp.	
0	0	15	0	0	0	0	0	0	0	0	0	Bacteria	Proteobacteria	Gammaproteobacteria	Alteromonadales	Idiomarinaceae	Aliidiomarina	Aliidiomarina	sp.	
0	0	0	41	0	0	0	0	0	0	0	0	Bacteria	Proteobacteria	Gammaproteobacteria	Alteromonadales	Alteromonadaceae	Alteromonas	Alteromonas	sp.	
22	0	49	0	0	0	0	0	0	0	0	0	Bacteria	Proteobacteria	Gammaproteobacteria	Alteromonadales	Alteromonadaceae	Alteromonas	Alteromonas	halophila	
0	0	19	0	0	0	0	0	0	0	0	0	Bacteria	Proteobacteria	Alphaproteobacteria	Rhizobiales	Rhizobiales Incertae Sedis	Anderseniella	Anderseniella	sp.	
0	0	0	0	47	43	0	0	72	23	0	65	Bacteria	Proteobacteria	Gammaproteobacteria	Nitrococcales	Nitrococcaceae	Aquisalimonas	Aquisalimonas	sp.	
98	134	30	0	0	0	0	0	0	0	0	0	Bacteria	Proteobacteria	Alphaproteobacteria	Rhodobacterales	Rhodobacteraceae	Ascidiaceihabitans	Ascidiaceihabitans	sp.	
51	0	0	0	0	0	0	0	0	0	0	0	Bacteria	Proteobacteria	Gammaproteobacteria	unclassified_Bacteria	unclassified_Bacteria	AT-s2-59			
0	0	0	0	0	0	0	0	0	0	0	17	Bacteria	Proteobacteria	Gammaproteobacteria	unclassified_Bacteria	unclassified_Bacteria	AT-s2-59			
0	0	0	0	0	0	0	0	0	0	28	0	Bacteria	Proteobacteria	Gammaproteobacteria	unclassified_Bacteria	unclassified_Bacteria	B2M28			
17	0	0	0	0	0	0	0	0	0	0	0	Bacteria	Proteobacteria	Gammaproteobacteria	Cellvibrionales	Spongiibacteraceae	BD1-7 clade			
0	0	0	0	0	0	0	19	0	0	0	0	Bacteria	Proteobacteria	Gammaproteobacteria	Cellvibrionales	unclassified_Bacteria	BD2-7			
71	0	0	65	0	0	0	0	0	0	0	0	Bacteria	Proteobacteria	Gammaproteobacteria	Cellvibrionales	unclassified_Bacteria	BD2-7			
0	0	27	0	0	0	0	0	0	0	0	0	Bacteria	Proteobacteria	Gammaproteobacteria	Oceanospirillales	Saccharospirillaceae	Bermanella	Bermanella	marisrubri	
0	0	0	0	0	0	0	0	25	0	15	39	Bacteria	Proteobacteria	Alphaproteobacteria	Rhodospirillales	Rhodospirillaceae	Caenispirillum	Caenispirillum	sp.	
0	0	17	0	0	0	0	0	0	0	0	0	Bacteria	Proteobacteria	Alphaproteobacteria	Rhodospirillales	Rhodospirillaceae	Caenispirillum	Caenispirillum	sp.	
26	0	0	0	0	0	0	0	0	0	0	0	Bacteria	Proteobacteria	Gammaproteobacteria	Cellvibrionales	Cellvibrionaceae	Candidatus Endobugula			
19	0	0	0	0	0	0	0	0	0	0	0	Bacteria	Proteobacteria	Gammaproteobacteria	Tenderiales	Tenderiaceae	Candidatus Tenderia			
64	54	0	0	0	0	0	0	0	0	0	0	Bacteria	Proteobacteria	Gammaproteobacteria	Chromatiales	Chromatiaceae	Candidatus Thiobios			
0	82	0	81	0	0	0	0	0	0	0	0	Bacteria	Proteobacteria	Gammaproteobacteria	Chromatiales	Chromatiaceae	Candidatus Thiobios			
0	0	0	0	0	0	43	0	0	0	0	0	Bacteria	Proteobacteria	Gammaproteobacteria	Arenicellales	Arenicellaceae	Candidatus Thiosymbion			
0	0	0	0	77	0	0	0	0	0	35	108	Bacteria	Proteobacteria	Gammaproteobacteria	Arenicellales	Arenicellaceae	Candidatus Thiosymbion			
0	0	0	0	0	0	0	0	0	0	33	0	Bacteria	Proteobacteria	Gammaproteobacteria	Arenicellales	Arenicellaceae	Candidatus Thiosymbion			
0	0	0	19	0	0	0	0	0	0	0	0	Bacteria	Proteobacteria	Gammaproteobacteria	Chromatiales	Chromatiaceae	Chromatium	Chromatium	sp.	NZ
158	59	0	116	69	112	0	0	78	53	41	0	Bacteria	Proteobacteria	Gammaproteobacteria	Cellvibrionales	Halieaceae	Congregibacter	Congregibacter	sp.	
0	0	0	0	0	0	0	15	0	0	0	0	Bacteria	Proteobacteria	Gammaproteobacteria	Coxiellales	Coxiellaceae	Coxiella	Coxiella	sp.	
0	0	0	0	0	0	0	0	32	0	0	0	Bacteria	Proteobacteria	Gammaproteobacteria	Coxiellales	Coxiellaceae	Coxiella	Coxiella	sp.	
0	0	0	0	18	13	0	0	0	0	0	0	Bacteria	Proteobacteria	Gammaproteobacteria	Coxiellales	Coxiellaceae	Coxiella	Coxiella	sp.	
0	0	0	0	0	0	47	0	0	0	0	0	Bacteria	Proteobacteria	Alphaproteobacteria	Defluviicoccales	Defluviicoccaceae	Defluviicoccus	Defluviicoccus	sp.	
0	0	0	0	46	0	86	72	0	0	40	73	Bacteria	Proteobacteria	Alphaproteobacteria	Defluviicoccales	Defluviicoccaceae	Defluviicoccus	Defluviicoccus	sp.	
0	0	0	59	0	0	0	0	0	0	0	0	Bacteria	Proteobacteria	Gammaproteobacteria	unclassified_Bacteria	unclassified_Bacteria	eub62A3			
0	10	0	14	0	0	0	0	0	0	0	0	Bacteria	Proteobacteria	Gammaproteobacteria	unclassified_Bacteria	unclassified_Bacteria	eub62A3			
0	0	0	0	0	0	0	21	0	0	0	0	Bacteria	Proteobacteria	Alphaproteobacteria	Caulobacterales	Hyphomonadaceae	Euryhalocaulis	Euryhalocaulis	caribicus	
0	33	30	0	38	0	0	45	43	0	0	43	Bacteria	Proteobacteria	Alphaproteobacteria	Rhizobiales	Hyphomicrobiaceae	Filomicrobium	Filomicrobium	sp.	
0	0	0	0	35	0	0	0	0	36	0	61	Bacteria	Proteobacteria	Alphaproteobacteria	Kiloniellales	Fodinicurvataceae	Fodinicurvata	Fodinicurvata	sp.	
0	0	55	0	0	0	0	0	0	36	11	13	Bacteria	Proteobacteria	Gammaproteobacteria	Halothiobacillales	Thioalkalibacteraceae	Guyparkeria	Guyparkeria	sp.	
0	0	0	0	0	0	0	22	0	0	0	0	Bacteria	Proteobacteria	Gammaproteobacteria	Oceanospirillales	Hahellaceae	Hahella	Hahella	sp.	
0	92	55	0	48	86	0	57	85	45	0	65	Bacteria	Proteobacteria	Gammaproteobacteria	Chromatiales	Chromatiaceae	Halochromatium	Halochromatium	sp.	
0	0	0	67	0	57	0	0	0	52	0	53	Bacteria	Proteobacteria	Gammaproteobacteria	Chromatiales	Chromatiaceae	Halochromatium	Halochromatium	glycolicum	
0	73	0	65	0	0	0	0	0	0	0	0	Bacteria	Proteobacteria	Gammaproteobacteria	Chromatiales	Chromatiaceae	Halochromatium	Halochromatium	sp.	
0	0	0	29	0	0	0	0	0	0	0	0	Bacteria	Proteobacteria	Gammaproteobacteria	Chromatiales	Chromatiaceae	Halochromatium	Halochromatium	sp.	
0	0	0	49	0	0	0	0	0	0	0	0	Bacteria	Proteobacteria	Gammaproteobacteria	Chromatiales	Chromatiaceae	Halochromatium	Halochromatium	sp.	
0	0	0	0	25	0	0	0	44	51	0	0	Bacteria	Proteobacteria	Alphaproteobacteria	Rhodobacterales	Rhodobacteraceae	Halodurantibacterium	Halodurantibacterium	sp.	

0	62	0	236	60	0	0	0	0	0	0	0	Bacteria	Proteobacteria	Alphaproteobacteria	Rhodobacterales	Rhodobacteraceae	Halodurantibacterium	Halodurantibacterium	sp.	
0	0	0	0	0	0	0	47	37	0	41	30	Bacteria	Proteobacteria	Gammaproteobacteria	Ectothiorhodospirales	Ectothiorhodospiraceae	Halofilum	Halofilum	sp.	
0	0	0	0	0	103	36	0	91	73	67	70	Bacteria	Proteobacteria	Gammaproteobacteria	Ectothiorhodospirales	Ectothiorhodospiraceae	Halofilum	Halofilum	sp.	
0	0	135	0	0	0	0	0	0	0	0	0	Bacteria	Proteobacteria	Gammaproteobacteria	Oceanospirillales	Halomonadaceae	Halomonadaceae	Marinospirillum	sp.	F3212
0	0	107	18	0	0	0	0	0	8	14	0	Bacteria	Proteobacteria	Gammaproteobacteria	Oceanospirillales	Halomonadaceae	Halomonas	Halomonas	sp.	
0	0	0	0	0	0	0	0	0	8	0	0	Bacteria	Proteobacteria	Alphaproteobacteria	Caulobacterales	Hyphomonadaceae	Henriciella	Henriciella	sp.	
0	0	128	69	0	0	0	0	0	0	0	0	Bacteria	Proteobacteria	Gammaproteobacteria	Alteromonadales	Idiomarinaceae	Idiomarina	Idiomarina	sp.	
0	0	36	0	0	0	0	0	0	0	0	0	Bacteria	Proteobacteria	Gammaproteobacteria	Alteromonadales	Idiomarinaceae	Idiomarina	Idiomarina	zobellii	
0	0	0	0	0	0	0	0	13	0	14	0	Bacteria	Proteobacteria	Gammaproteobacteria	Steroidobacterales	Woeseiaceae	JTB255 marine benthic group			
0	0	0	0	0	0	0	37	0	43	48	0	Bacteria	Proteobacteria	Gammaproteobacteria	Oceanospirillales	Kangiellaceae	Kangiella	Kangiella	sp.	
0	0	0	71	0	0	0	0	0	0	0	0	Bacteria	Proteobacteria	Alphaproteobacteria	Rhodobacterales	Rhodobacteraceae	Limibaculum	Limibaculum	sp.	
0	22	0	82	0	0	0	33	0	0	31	0	Bacteria	Proteobacteria	Alphaproteobacteria	Rhodobacterales	Rhodobacteraceae	Limibaculum	Limibaculum	sp.	
0	0	0	0	0	0	124	0	0	0	0	41	Bacteria	Proteobacteria	Alphaproteobacteria	Rhodobacterales	Rhodobacteraceae	Limibaculum	Limibaculum	sp.	
0	0	0	62	0	0	0	0	0	0	18	0	Bacteria	Proteobacteria	Alphaproteobacteria	Rhodobacterales	Rhodobacteraceae	Limibaculum	Limibaculum	sp.	
0	0	0	32	0	0	0	0	0	0	0	0	Bacteria	Proteobacteria	Gammaproteobacteria	Cellvibrionales	Halieaceae	Luminiphilus	Luminiphilus	sp.	
0	0	0	0	0	0	0	0	16	0	0	0	Bacteria	Proteobacteria	Alphaproteobacteria	Rhodospirillales	Magnetospiraceae	Magnetospira	Magnetospira	sp.	
82	0	0	0	0	0	0	0	0	0	0	0	Bacteria	Proteobacteria	Alphaproteobacteria	Rhodobacterales	Rhodobacteraceae	Mameliella	Mameliella	sp.	
0	0	0	47	0	0	0	0	0	0	0	0	Bacteria	Proteobacteria	Alphaproteobacteria	Caulobacterales	Hyphomonadaceae	Marinicauda	Marinicauda	sp.	WD6-1
147	222	444	127	99	72	0	106	0	193	0	62	Bacteria	Proteobacteria	Gammaproteobacteria	Alteromonadales	Marinobacteraceae	Marinobacter	Marinobacter	sp.	
0	0	1623	0	0	0	0	0	0	35	0	0	Bacteria	Proteobacteria	Gammaproteobacteria	Alteromonadales	Marinobacteraceae	Marinobacter	Marinobacter	aquaticus	
0	0	30	0	0	0	0	0	0	0	0	0	Bacteria	Proteobacteria	Gammaproteobacteria	Oceanospirillales	Nitrincolaceae	Marinobacterium	Marinobacterium	sp.	AK62
0	0	84	0	0	0	0	0	0	0	0	0	Bacteria	Proteobacteria	Gammaproteobacteria	Oceanospirillales	Nitrincolaceae	Marinobacterium	Marinobacterium	stanieri	
8	0	0	0	0	0	0	22	19	0	0	19	Bacteria	Proteobacteria	Alphaproteobacteria	Rhodospirillales	Terasakiellaceae	Marivibrio	Marivibrio	halodurans	
0	13	0	0	0	0	0	0	0	42	0	43	Bacteria	Proteobacteria	Alphaproteobacteria	Rhodobacterales	Rhodobacteraceae	Marivita	Marivita	sp.	
62	59	0	0	0	0	0	0	0	0	0	0	Bacteria	Proteobacteria	Alphaproteobacteria	Rhizobiales	Methyloligellaceae	Methyloceanibacter	Methyloceanibacter	sp.	
0	0	39	0	0	0	0	0	0	0	0	0	Bacteria	Proteobacteria	Gammaproteobacteria	Nitrosococcales	Methylophagaceae	Methylophaga	Methylophaga	sp.	
0	77	36	0	0	0	0	0	0	0	0	0	Bacteria	Proteobacteria	Gammaproteobacteria	unclassified_Bacteria	unclassified_Bacteria	Milano-WF1B-44			
0	0	0	0	0	0	37	0	0	0	0	0	Bacteria	Proteobacteria	Gammaproteobacteria	Nitrosococcales	Nitrosococcaceae	Nitrosococcus	Nitrosococcus	sp.	
24	32	0	86	0	28	0	0	0	41	0	0	Bacteria	Proteobacteria	Alphaproteobacteria	Caulobacterales	Hyphomonadaceae	Oceanicaulis	Oceanicaulis	sp.	
0	0	0	34	0	0	0	0	29	0	87	37	Bacteria	Proteobacteria	Alphaproteobacteria	Caulobacterales	Hyphomonadaceae	Oceanicaulis	Oceanicaulis	sp.	
0	80	0	49	0	37	0	0	0	0	19	0	Bacteria	Proteobacteria	Alphaproteobacteria	Caulobacterales	Hyphomonadaceae	Oceanicaulis	Oceanicaulis	sp.	
0	0	147	0	0	0	0	0	0	0	0	0	Bacteria	Proteobacteria	Gammaproteobacteria	Oceanospirillales	Oceanospirillaceae	Oceanospirillum	Oceanospirillum	sp.	
0	0	58	0	0	0	0	0	0	0	0	0	Bacteria	Proteobacteria	Alphaproteobacteria	Rhodobacterales	Rhodobacteraceae	Palleronia-Pseudomaribius	sp.	A14	
0	0	0	52	0	0	0	17	0	0	27	27	Bacteria	Proteobacteria	Alphaproteobacteria	Caulobacterales	Parvularculaceae	Parvularcula	Parvularcula	sp.	
0	0	0	0	0	0	0	62	92	56	79	24	Bacteria	Proteobacteria	Alphaproteobacteria	Caulobacterales	Parvularculaceae	Parvularcula	Parvularcula	sp.	
0	0	0	0	0	0	0	36	0	0	0	0	Bacteria	Proteobacteria	Alphaproteobacteria	Caulobacterales	Parvularculaceae	Parvularcula	Parvularcula	sp.	TAA-239
0	0	0	71	0	0	0	0	0	0	0	0	Bacteria	Proteobacteria	Alphaproteobacteria	Caulobacterales	Parvularculaceae	Parvularcula	Parvularcula	sp.	
0	0	47	0	0	0	0	0	0	0	0	0	Bacteria	Proteobacteria	Gammaproteobacteria	Alteromonadales	Idiomarinaceae	Pseudidiomarina	Pseudidiomarina	marina	
0	0	76	57	0	0	0	0	0	0	0	0	Bacteria	Proteobacteria	Gammaproteobacteria	Alteromonadales	Pseudoalteromonadaceae	Pseudoalteromonas	Pseudoalteromonas	sp.	
0	26	0	0	0	0	20	0	0	42	0	0	Bacteria	Proteobacteria	Gammaproteobacteria	Oceanospirillales	Pseudohongiellaceae	Pseudohongiella	Pseudohongiella	sp.	
11	0	0	0	0	0	0	0	0	0	0	0	Bacteria	Proteobacteria	Gammaproteobacteria	Oceanospirillales	Pseudohongiellaceae	Pseudohongiella	Pseudohongiella	sp.	DG1301
0	24	0	0	0	29	0	0	0	0	0	0	Bacteria	Proteobacteria	Gammaproteobacteria	Oceanospirillales	Pseudohongiellaceae	Pseudohongiella	Pseudohongiella	sp.	
0	0	0	12	0	0	0	0	0	0	0	0	Bacteria	Proteobacteria	Gammaproteobacteria	Oceanospirillales	Pseudohongiellaceae	Pseudospirillum	Pseudospirillum	japonicum	
0	0	0	0	0	0	0	0	0	0	18	0	Bacteria	Proteobacteria	Alphaproteobacteria	Rhizobiales	Rhodomicrobiaceae	Rhodomicrobium	Rhodomicrobium	sp.	
0	0	0	0	0	15	0	0	0	0	0	0	Bacteria	Proteobacteria	Alphaproteobacteria	Rhizobiales	Rhodomicrobiaceae	Rhodomicrobium	Rhodomicrobium	sp.	
0	62	80	73	188	582	109	577	383	825	1365	492	Bacteria	Proteobacteria	Alphaproteobacteria	Kiloniellales	Fodinicurvataceae	Rhodovibrio	Rhodovibrio	sp.	
0	0	0	0	68	0	0	113	64	37	53	42	Bacteria	Proteobacteria	Alphaproteobacteria	Kiloniellales	Fodinicurvataceae	Rhodovibrio	Rhodovibrio	sp.	2Mb1
0	0	0	18	0	22	0	0	0	0	17	0	Bacteria	Proteobacteria	Alphaproteobacteria	Kiloniellales	Fodinicurvataceae	Rhodovibrio	Rhodovibrio	sp.	
59	54	0	0	0	0	0	0	7	0	22	0	Bacteria	Proteobacteria	Alphaproteobacteria	Rhodobacterales	Rhodobacteraceae	Rhodovulum	Rhodovulum	sp.	JZ3A21
0	0	0	0	0	0	32	92	183	0	0	31	Bacteria	Proteobacteria	Alphaproteobacteria	Rhodobacterales	Rhodobacteraceae	Rhodovulum	Rhodovulum	sp.	
0	0	0	0	0	0	0	0	0	0	0	42	Bacteria	Proteobacteria	Alphaproteobacteria	Rhodobacterales	Rhodobacteraceae	Roseivivax	Roseivivax	sp.	
0	0	30	0	0	0	0	0	0	0	0	0	Bacteria	Proteobacteria	Alphaproteobacteria	Rhodobacterales	Rhodobacteraceae	Roseivivax	Roseivivax	roseus	
0	0	0	30	0	0	0	0	0	0	0	0	Bacteria	Proteobacteria	Alphaproteobacteria	Rhodobacterales	Rhodobacteraceae	Roseovarius	Roseovarius	sp.	
0	0	0	38	0	0	0	0	0	0	0	0	Bacteria	Proteobacteria	Alphaproteobacteria	Rhodobacterales	Rhodobacteraceae	Rubribacterium	Rubribacterium	sp.	
0	52	0	78	0	0	0	0	0	16	0	0	Bacteria	Proteobacteria	Alphaproteobacteria	Rhodobacterales	Rhodobacteraceae	Rubribacterium	Rubribacterium	sp.	
0	0	0	0	0	0	0	94	0	32	0	0	Bacteria	Proteobacteria	Alphaproteobacteria	Rhodobacterales	Rhodobacteraceae	Ruegeria	Ruegeria	sp.	
42	0	0	0	0	0	0	0	0	0	0	0	Bacteria	Proteobacteria	Gammaproteobacteria	Oceanospirillales	Saccharospirillaceae	Saccharospirillum	Saccharospirillum	aestuarii	
0	0	100	0	0	0	0	0	43	0	5	0	Bacteria	Proteobacteria	Gammaproteobacteria	Oceanospirillales	Saccharospirillaceae	Saccharospirillum	Saccharospirillum	salsuginis	
0	0	0	0	0	0	32	0	11	0	0	24	Bacteria	Proteobacteria	Alphaproteobacteria	Rhizobiales	Beijerinckiaceae	Salinarimonas	Salinarimonas	sp.	
0	0	77	0	30	67	0	0	0	0	0	0	Bacteria	Proteobacteria	Gammaproteobacteria	Alteromonadales	Alteromonadaceae	Salinimonas	Salinimonas	sp.	HMF8227
9	27	0	0	0	0	0	0	0	0	0	0	Bacteria	Proteobacteria	Gammaproteobacteria	Salinisphaerales	Salinisphaeraceae	Salinisphaera	Salinisphaera	sp.	
0	0	0	70	0	0	0	0	0	0	0	0	Bacteria	Proteobacteria	Gammaproteobacteria	Vibrionales	Vibrionaceae	Salinivibrio	Salinivibrio	sp.	
0	0	0	0	0	6	0	15	0	0	0	0	Bacteria	Proteobacteria	Alphaproteobacteria	Rickettsiales	unclassified_Bacteria	SM2D12			
0	0	0	0	0	0	0	0	0	0	14	0	Bacteria	Proteobacteria	Alphaproteobacteria	Rickettsiales	unclassified_Bacteria	SM2D12			
9	0	0	20	0	0	0	0	0	0	0	0	Bacteria	Proteobacteria	Alphaproteobacteria	Rickettsiales	unclassified_Bacteria	SM2D12			
0	0	0	0	0	0	0	9	0	0	0	0	Bacteria	Proteobacteria	Alphaproteobacteria	Sneathiellales	Sneathiellaceae	Sneathiella	Sneathiella	sp.	
0	0	37	0	0	0	0	0	0	0	0	0	Bacteria	Proteobacteria	Gammaproteobacteria	Oceanospirillales	Halomonadaceae	Terasakiispira	Terasakiispira	sp.	
0	5	0	0	0	0	0	0	0	0	0	0	Bacteria	Proteobacteria	Gammaproteobacteria	Oceanospirillales	Saccharospirillaceae	Thalassolituus	Bacterioplanes	sanyensis	
0	0	0	0	0	62	0	0	0	0	0	0	Bacteria	Proteobacteria	Gammaproteobacteria	Chromatiales	Chromatiaceae	Thiohalocapsa	Thiohalocapsa	sp.	
0	0	0	0	28	0	0	0	0	0	0	0	Bacteria	Proteobacteria	Gammaproteobacteria	Ectothiorhodospirales	Thioalkalispiraceae	Thiohalophilus	Thiohalophilus	thiocyanatoxydans	
0	0	0	0	8	0	16	0	0	16	0	0	Bacteria	Proteobacteria	Gammaproteobacteria	Thiohalorhabdales	Thiohalorhabdaceae	Thiohalorhabdus	Thiohalorhabdus	sp.	
0	0	0	0	0	0	51	0	0	0	0	0	Bacteria	Proteobacteria	Gammaproteobacteria	Arenicellales	Arenicellaceae	Thiolapillus	Thiolapillus	sp.	
0	0	0	79	0	0	0	0	0	0	0	0	Bacteria	Proteobacteria	Gammaproteobacteria	Thiomicrospirales	Thiomicrospiraceae	Thiomicrospira	Thiomicrospira	sp.	
0	0	34	0	0	0	0	0	0	0	0	0	Bacteria	Proteobacteria	Gammaproteobacteria	Thiomicrospirales	Thiomicrospiraceae	Thiomicrospira	Thiomicrospira	sp.	
0	0	0	0	0	31	0	0	0	0	0	0	Bacteria	Proteobacteria	Gammaproteobacteria	Chromatiales	Chromatiaceae	Thiophaeococcus	Thiophaeococcus	sp.	JA592
0	0	0	33	0	0	0	0	0	0	0	0	Bacteria	Proteobacteria	Gammaproteobacteria	Chromatiales	Chromatiaceae	Thiorhodovibrio	Thiorhodovibrio	sp.	
0	0	48	59	0	0	0	0	0	0	0	0	Bacteria	Proteobacteria	Gammaproteobacteria	Vibrionales	Vibrionaceae	Vibrio	Vibrio	sp.	
0	0	0	62	0	0	0	0	0	0	0	0	Bacteria	Proteobacteria	Gammaproteobacteria	Gammaproteobacteria Incertae Sedis	unclassified_Bacteria	Wenzhouxiangella	Wenzhouxiangella	sp.	
0	0	0	99	0	0	0	28	0	0	0	15	Bacteria	Proteobacteria	Gammaproteobacteria	Gammaproteobacteria Incertae Sedis	unclassified_Bacteria	Wenzhouxiangella	Wenzhouxiangella	sp.	
0	0	0	0	0	0	0	0	69	0	0	0	Bacteria	Proteobacteria	Gammaproteobacteria	unclassified_Bacteria	unclassified_Bacteria	WN-HWB-116			
284	177	91	0	87	39	62	168	82	0	65	32	Bacteria	Proteobacteria	Gammaproteobacteria	Steroidobacterales	Woeseiaceae	Woeseia	Woeseia	sp.	
23	28	0	0	46	50	17	44	40	0	92	0	Bacteria	Proteobacteria	Gammaproteobacteria	Steroidobacterales	Woeseiaceae	Woeseia	Woeseia	sp.	
0	0	0	0	0	0	0	0	0	0	0	68	Bacteria	Proteobacteria	Alphaproteobacteria	Alphaproteobacteria Incertae Sedis	unclassified_Bacteria				
3	0	0	0	0	0	0	0	0	0	0	0	Bacteria	Proteobacteria	Alphaproteobacteria	Alphaproteobacteria Incertae Sedis	unclassified_Bacteria				
0	0	38	0	0	0	0	0	0	0	0	0	Bacteria	Proteobacteria	Gammaproteobacteria	Alteromonadales	Alteromonadaceae				
0	0	0	0	0	15	0	0	0	0	0	0	Bacteria	Proteobacteria	Gammaproteobacteria	Arenicellales	Arenicellaceae				
0	0	0	0	0	0	0	48	0	0	0	10	Bacteria	Proteobacteria	Alphaproteobacteria	Azospirillales	Azospirillaceae				
0	0	0	0	0	36	0	0	0	0	0	0	Bacteria	Proteobacteria	Gammaproteobacteria	Beggiatoales	Beggiatoaceae				
32	37	0	55	0	0	0	0	0	0	0	0	Bacteria	Proteobacteria	Alphaproteobacteria	Caulobacterales	Hyphomonadaceae				
43	33	0	0	0	0	0	0	44	0	41	0	Bacteria	Proteobacteria	Alphaproteobacteria	Caulobacterales	Hyphomonadaceae				
0	0	0	52	0	0	0	0	0	0	0	0	Bacteria	Proteobacteria	Alphaproteobacteria	Caulobacterales	Hyphomonadaceae				
0	19	0	80	0	0	0	19	16	0	18	0	Bacteria	Proteobacteria	Alphaproteobacteria	Caulobacterales	Hyphomonadaceae				
0	0	0	0	0	0	0	8	0	0	0	0	Bacteria	Proteobacteria	Gammaproteobacteria	Cellvibrionales	Halieaceae				
0	0	0	56	0	0	0	116	30	0	43	0	Bacteria	Proteobacteria	Gammaproteobacteria	Cellvibrionales	Halieaceae				
0	0	0	0	0	0	0	39	60	0	0	39	Bacteria	Proteobacteria	Gammaproteobacteria	Cellvibrionales	Spongiibacteraceae				
0	0	0	0	0	25	0	0	0	0	0	0	Bacteria	Proteobacteria	Gammaproteobacteria	Cellvibrionales	Spongiibacteraceae				
0	0	0	0	0	0	0	0	76	75	50	87	Bacteria	Proteobacteria	Gammaproteobacteria	Cellvibrionales	Spongiibacteraceae				
0	0	0	0	0	0	0	36	0	0	0	0	Bacteria	Proteobacteria	Gammaproteobacteria	Cellvibrionales	Spongiibacteraceae				
0	0	0	93	0	0	0	0	0	0	0	0	Bacteria	Proteobacteria	Gammaproteobacteria	Chromatiales	Chromatiaceae				
0	151	0	193	135	190	56	0	58	115	121	0	Bacteria	Proteobacteria	Gammaproteobacteria	Chromatiales	Chromatiaceae				
0	0	0	45	0	0	0	0	0	0	0	0	Bacteria	Proteobacteria	Gammaproteobacteria	Chromatiales	Chromatiaceae				
0	0	0	0	0	0	0	24	35	0	0	0	Bacteria	Proteobacteria	Alphaproteobacteria	Defluviicoccales	unclassified_Bacteria				
25	24	0	0	0	0	0	0	0	0	48	0	Bacteria	Proteobacteria	Alphaproteobacteria	Defluviicoccales	unclassified_Bacteria				
0	0	14	0	0	0	0	0	0	0	0	0	Bacteria	Proteobacteria	Gammaproteobacteria	Ectothiorhodospirales	Ectothiorhodospiraceae				
26	0	0	0	0	0	0	0	0	0	0	65	Bacteria	Proteobacteria	Gammaproteobacteria	Gammaproteobacteria Incertae Sedis	unclassified_Bacteria				
0	43	0	0	0	0	70	0	0	0	0	0	Bacteria	Proteobacteria	Gammaproteobacteria	Gammaproteobacteria Incertae Sedis	unclassified_Bacteria				YC-ZSS-LKJ56
0	85	0	0	0	0	0	0	0	0	0	0	Bacteria	Proteobacteria	Gammaproteobacteria	Gammaproteobacteria Incertae Sedis	unclassified_Bacteria				25(2013)
0	0	0	0	0	0	0	18	0	0	0	0	Bacteria	Proteobacteria	Gammaproteobacteria	Gammaproteobacteria Incertae Sedis	unclassified_Bacteria				
0	0	0	55	85	116	0	53	0	0	121	0	Bacteria	Proteobacteria	Gammaproteobacteria	Gammaproteobacteria Incertae Sedis	unclassified_Bacteria				
0	0	0	0	0	0	0	0	0	0	0	42	Bacteria	Proteobacteria	Gammaproteobacteria	Gammaproteobacteria Incertae Sedis	unclassified_Bacteria				
67	0	0	0	26	0	0	0	0	0	0	0	Bacteria	Proteobacteria	Gammaproteobacteria	HOC36	unclassified_Bacteria				
0	0	0	0	0	0	23	347	0	0	27	0	Bacteria	Proteobacteria	Gammaproteobacteria	HOC36	unclassified_Bacteria				
25	0	0	0	0	0	45	198	157	80	0	101	Bacteria	Proteobacteria	Gammaproteobacteria	HOC36	unclassified_Bacteria				
0	0	0	0	0	0	55	13	0	0	0	14	Bacteria	Proteobacteria	Gammaproteobacteria	KI89A clade	unclassified_Bacteria				
0	0	0	0	21	0	65	0	0	77	34		Bacteria	Proteobacteria	Gammaproteobacteria	KI89A clade	unclassified_Bacteria				
0	0	0	0	0	0	0	11	0	0	0	0	Bacteria	Proteobacteria	Alphaproteobacteria	Kiloniellales	Fodinicurvataceae				
33	51	35	0	0	0	0	77	0	0	73	0	Bacteria	Proteobacteria	Alphaproteobacteria	Kiloniellales	Kiloniellaceae				
0	0	0	0	0	0	0	0	27	0	0	0	Bacteria	Proteobacteria	Alphaproteobacteria	Kiloniellales	unclassified_Bacteria				
0	0	0	48	0	0	0	0	0	5	0	22	Bacteria	Proteobacteria	Alphaproteobacteria	Micavibrionales	Micavibrionaceae				
0	0	0	0	0	0	0	0	54	0	0	0	Bacteria	Proteobacteria	Alphaproteobacteria	Micavibrionales	Micavibrionaceae				
0	0	0	0	0	0	0	61	0	0	56	0	Bacteria	Proteobacteria	Alphaproteobacteria	Micavibrionales	Micavibrionaceae				
0	0	0	0	0	0	0	0	0	0	0	9	Bacteria	Proteobacteria	Alphaproteobacteria	Micavibrionales	Micavibrionaceae				
3	13	0	265	0	22	0	0	0	60	0	0	Bacteria	Proteobacteria	Alphaproteobacteria	Micavibrionales	Micavibrionaceae				
44	24	0	0	0	0	0	0	0	23	0	0	Bacteria	Proteobacteria	Alphaproteobacteria	Micavibrionales	Micavibrionaceae				
0	0	0	0	0	0	0	13	0	0	0	0	Bacteria	Proteobacteria	Alphaproteobacteria	Micavibrionales	Micavibrionaceae				
0	0	0	43	0	0	0	0	0	0	0	0	Bacteria	Proteobacteria	Alphaproteobacteria	Micavibrionales	unclassified_Bacteria				
0	0	0	0	0	0	80	97	0	14	0	0	Bacteria	Proteobacteria	Gammaproteobacteria	Nitrococcales	Nitrococcaceae				
0	0	0	0	0	0	0	28	0	0	0	0	Bacteria	Proteobacteria	Gammaproteobacteria	Nitrosococcales	Nitrosococcaceae				
0	0	0	0	0	0	54	0	0	0	0	0	Bacteria	Proteobacteria	Gammaproteobacteria	Nitrosococcales	Nitrosococcaceae				3PJM14
0	0	0	0	108	0	20	41	0	0	0	146	Bacteria	Proteobacteria	Gammaproteobacteria	Nitrosococcales	Nitrosococcaceae				CI75cm.2.12
0	0	0	0	0	0	0	0	0	0	100	46	Bacteria	Proteobacteria	Gammaproteobacteria	Nitrosococcales	Nitrosococcaceae				CI75cm.2.12
0	0	0	0	0	0	0	0	2	0	0	0	Bacteria	Proteobacteria	Gammaproteobacteria	Oceanospirillales	Halomonadaceae				
0	53	81	0	22	0	51	13	0	82	0	0	Bacteria	Proteobacteria	Gammaproteobacteria	Oceanospirillales	Halomonadaceae				
0	0	0	0	79	36	0	0	43	0	0	0	Bacteria	Proteobacteria	Gammaproteobacteria	Oceanospirillales	unclassified_Bacteria				
33	43	0	11	0	0	0	19	0	0	0	0	Bacteria	Proteobacteria	Gammaproteobacteria	OM182 clade	unclassified_Bacteria				
0	0	10	0	0	0	0	0	0	0	0	0	Bacteria	Proteobacteria	Alphaproteobacteria	Paracaedibacterales	Paracaedibacteraceae				
0	0	0	0	0	0	0	0	0	0	0	12	Bacteria	Proteobacteria	Alphaproteobacteria	Puniceispirillales	unclassified_Bacteria				
0	0	0	0	0	0	0	0	0	0	0	5	Bacteria	Proteobacteria	Alphaproteobacteria	Puniceispirillales	unclassified_Bacteria				
0	13	0	0	0	29	0	0	0	0	0	0	Bacteria	Proteobacteria	Alphaproteobacteria	Rhizobiales	Methyloligellaceae				
0	0	0	23	0	0	0	0	0	0	0	0	Bacteria	Proteobacteria	Alphaproteobacteria	Rhizobiales	Rhizobiaceae				
0	0	0	21	0	0	0	7	0	0	9	0	Bacteria	Proteobacteria	Alphaproteobacteria	Rhizobiales	unclassified_Bacteria				
167	1222	191	1975	256	491	187	189	531	659	560	372	Bacteria	Proteobacteria	Alphaproteobacteria	Rhodobacterales	Rhodobacteraceae				
0	0	0	45	0	0	0	0	0	58	62	0	Bacteria	Proteobacteria	Alphaproteobacteria	Rhodobacterales	Rhodobacteraceae				
0	0	0	0	0	0	0	0	5	0	0	0	Bacteria	Proteobacteria	Alphaproteobacteria	Rhodospirillales	Magnetospiraceae				

0	0	0	0	0	0	15	0	0	0	0	0	Bacteria	Proteobacteria	Alphaproteobacteria	Rhodospirillales	Magnetospiraceae				
0	0	0	0	0	9	0	0	0	0	0	0	Bacteria	Proteobacteria	Alphaproteobacteria	Rhodospirillales	Magnetospiraceae				
0	0	0	0	0	0	0	6	0	0	0	0	Bacteria	Proteobacteria	Alphaproteobacteria	Rhodospirillales	Rhodospirillaceae				
0	0	0	0	0	12	0	0	0	0	0	0	Bacteria	Proteobacteria	Alphaproteobacteria	Rhodospirillales	unclassified_Bacteria				
0	0	0	0	0	0	0	0	0	48	0	0	Bacteria	Proteobacteria	Alphaproteobacteria	Rhodospirillales	unclassified_Bacteria				
0	11	0	6	0	0	0	0	0	0	9	0	Bacteria	Proteobacteria	Alphaproteobacteria	Rhodospirillales	unclassified_Bacteria				
0	0	0	8	14	0	0	0	30	52	75	0	Bacteria	Proteobacteria	Alphaproteobacteria	Rhodospirillales	unclassified_Bacteria				
0	0	0	26	0	0	0	0	0	0	0	0	Bacteria	Proteobacteria	Alphaproteobacteria	Rickettsiales	Anaplasmataceae				
0	0	0	18	0	0	0	0	0	0	0	0	Bacteria	Proteobacteria	Alphaproteobacteria	Rickettsiales	Fokiniaceae				
0	0	0	0	0	0	0	7	0	0	0	0	Bacteria	Proteobacteria	Alphaproteobacteria	Rickettsiales	unclassified_Bacteria				
47	0	0	0	0	0	0	0	0	0	0	0	Bacteria	Proteobacteria	Gammaproteobacteria	Run-SP154	unclassified_Bacteria				
31	165	0	98	72	70	29	96	0	55	132	40	Bacteria	Proteobacteria	Alphaproteobacteria	Sphingomonadales	Sphingomonadaceae				
0	0	0	0	65	80	34	34	50	30	0	82	Bacteria	Proteobacteria	Alphaproteobacteria	Thalassobaculales	unclassified_Bacteria				
0	8	0	0	27	63	83	25	0	32	0	53	Bacteria	Proteobacteria	Alphaproteobacteria	Thalassobaculales	unclassified_Bacteria				
0	0	0	21	0	41	0	100	115	58	119	71	Bacteria	Proteobacteria	Alphaproteobacteria	Thalassobaculales	unclassified_Bacteria				
0	0	0	26	0	0	0	0	0	0	0	0	Bacteria	Proteobacteria	Gammaproteobacteria	Thiomicrospirales	Thiomicrospiraceae				
0	29	0	0	0	0	0	0	0	0	0	0	Bacteria	Proteobacteria	Gammaproteobacteria	Thiotrichales	Thiotrichaceae				
21	0	0	0	0	0	0	0	0	0	0	0	Bacteria	Proteobacteria	Gammaproteobacteria	Thiotrichales	Thiotrichaceae				
15	0	0	0	0	0	0	0	0	0	0	0	Bacteria	Proteobacteria	Alphaproteobacteria	Tistrellales	Geminicoccaceae				
0	113	0	0	0	12	13	21	0	0	6	0	Bacteria	Proteobacteria	Alphaproteobacteria	Tistrellales	Geminicoccaceae				
0	0	0	0	0	0	0	34	0	0	0	0	Bacteria	Proteobacteria	Alphaproteobacteria	Tistrellales	Geminicoccaceae				
0	0	0	19	0	0	0	0	0	0	0	0	Bacteria	Proteobacteria	Alphaproteobacteria	unclassified_Bacteria	unclassified_Bacteria				
37	2	0	193	46	29	14	104	76	28	69	63	Bacteria	Proteobacteria	Alphaproteobacteria	unclassified_Bacteria	unclassified_Bacteria				
76	0	34	35	0	0	0	0	0	0	0	0	Bacteria	Proteobacteria	Gammaproteobacteria	unclassified_Bacteria	unclassified_Bacteria				clone-35(2013)
0	0	0	0	0	0	5	0	0	0	0	0	Bacteria	Proteobacteria	Gammaproteobacteria	unclassified_Bacteria	unclassified_Bacteria				
25	136	61	189	42	75	215	190	119	45	314	211	Bacteria	Proteobacteria	Gammaproteobacteria	unclassified_Bacteria	unclassified_Bacteria				
0	0	0	26	10	4	0	0	0	0	0	0	Bacteria	Proteobacteria	unclassified_Bacteria	unclassified_Bacteria	unclassified_Bacteria				
0	0	0	0	114	117	0	77	61	0	37	85	Bacteria	Proteobacteria	Alphaproteobacteria	unclassified_Bacteria	unclassified_Bacteria				
0	0	0	0	0	0	0	19	0	0	0	0	Bacteria	Proteobacteria	Gammaproteobacteria	Xanthomonadales	Rhodanobacteraceae				
0	0	0	15	0	0	0	0	0	20	0	0	Bacteria	SAR324_clade(Marine group B)	SAR324_clade(Marine group B)	unclassified_Bacteria	unclassified_Bacteria				
0	0	0	29	0	0	9	14	14	0	0	0	Bacteria	SAR324_clade(Marine group B)	SAR324_clade(Marine group B)	unclassified_Bacteria	unclassified_Bacteria				
5	0	0	0	0	0	0	0	0	0	0	0	Bacteria	SAR324_clade(Marine group B)	SAR324_clade(Marine group B)	unclassified_Bacteria	unclassified_Bacteria				
0	0	0	0	0	0	0	41	0	0	0	18	Bacteria	SAR324_clade(Marine group B)	SAR324_clade(Marine group B)	unclassified_Bacteria	unclassified_Bacteria				
0	0	0	96	0	0	0	0	0	0	0	0	Bacteria	Spirochaetota	Spirochaetia	Spirochaetales	Spirochaetaceae	Alkalispirochaeta	Alkalispirochaeta	sp.	
0	0	0	0	0	0	0	0	0	8	10	18	Bacteria	Spirochaetota	Brachyspirae	Brachyspirales	Brachyspiraceae	Brachyspira	Brachyspira	sp.	
0	0	0	10	0	0	0	0	0	0	0	0	Bacteria	Spirochaetota	Brevinematia	Brevinematales	Brevinemataceae	Brevinema	Brevinema	sp.	
0	0	0	0	0	5	0	0	5	14	0	10	Bacteria	Spirochaetota	Brevinematia	Brevinematales	Brevinemataceae	Brevinema	Brevinema	sp.	
0	0	0	0	34	21	0	0	0	0	0	0	Bacteria	Spirochaetota	Spirochaetia	Spirochaetales	Spirochaetaceae	Candidatus Marispirochaeta			
0	0	0	0	61	76	0	0	0	0	0	0	Bacteria	Spirochaetota	Spirochaetia	Spirochaetales	Spirochaetaceae	Candidatus Marispirochaeta			
0	0	0	7	0	0	0	0	0	0	0	0	Bacteria	Spirochaetota	Brachyspirae	Brachyspirales	Brachyspirales Incertae Sedis	Exilispira	Exilispira	thermophila	
0	0	0	0	8	0	0	0	0	0	0	0	Bacteria	Spirochaetota	Brachyspirae	Brachyspirales	unclassified_Bacteria	LH041			
0	0	0	0	0	19	0	0	0	0	20	0	Bacteria	Spirochaetota	Brachyspirae	Brachyspirales	unclassified_Bacteria	LH041			
0	0	0	0	0	0	0	0	8	0	0	0	Bacteria	Spirochaetota	Brachyspirae	Brachyspirales	unclassified_Bacteria	LH041			
0	0	8	47	0	0	0	0	0	0	0	0	Bacteria	Spirochaetota	Spirochaetia	Spirochaetales	Spirochaetaceae	Salinispira	Salinispira	pacifica	
0	0	0	15	0	0	0	0	0	0	0	0	Bacteria	Spirochaetota	Spirochaetia	Spirochaetales	Spirochaetaceae	Sediminispirochaeta	Sediminispirochaeta	sp.	
0	0	0	55	0	22	0	0	24	0	0	35	Bacteria	Spirochaetota	Spirochaetia	Spirochaetales	Spirochaetaceae	Sediminispirochaeta	Sediminispirochaeta	sp.	
0	0	0	82	0	0	0	0	0	0	0	0	Bacteria	Spirochaetota	Spirochaetia	Spirochaetales	Spirochaetaceae	Sediminispirochaeta	Sediminispirochaeta	sp.	
0	0	0	0	0	21	0	7	0	0	0	0	Bacteria	Spirochaetota	Spirochaetia	Spirochaetales	Spirochaetaceae	Sediminispirochaeta	Sediminispirochaeta	sp.	
0	0	0	10	80	96	0	0	0	20	0	0	Bacteria	Spirochaetota	Spirochaetia	Spirochaetales	Spirochaetaceae	Sediminispirochaeta	Sediminispirochaeta	sp.	
0	0	0	0	0	0	0	0	0	8	0	0	Bacteria	Spirochaetota	Spirochaetia	Spirochaetales	Spirochaetaceae	Sediminispirochaeta	Sediminispirochaeta	sp.	
0	0	0	0	25	26	0	17	0	0	0	0	Bacteria	Spirochaetota	Spirochaetia	Spirochaetales	Spirochaetaceae	Spirochaeta	Spirochaeta	sp.	
2	0	0	53	73	46	0	0	26	77	17	61	Bacteria	Spirochaetota	Spirochaetia	Spirochaetales	Spirochaetaceae	Spirochaeta	Spirochaeta	sp.	
0	0	0	0	0	0	77	0	0	0	0	0	Bacteria	Spirochaetota	Spirochaetia	Spirochaetales	Spirochaetaceae	Spirochaeta	Spirochaeta	sp.	
0	0	0	0	11	26	0	0	0	3	0	10	Bacteria	Spirochaetota	Spirochaetia	Spirochaetales	Spirochaetaceae	Spirochaeta	Spirochaeta	sp.	
0	0	0	0	78	0	0	16	73	39	98	13	Bacteria	Spirochaetota	Spirochaetia	Spirochaetales	Spirochaetaceae	Spirochaeta	Spirochaeta	sp.	
17	0	5	0	158	74	0	41	48	157	110	131	Bacteria	Spirochaetota	Spirochaetia	Spirochaetales	Spirochaetaceae	Spirochaeta	Spirochaeta	sp.	
0	66	0	121	68	223	51	52	55	31	120	179	Bacteria	Spirochaetota	Spirochaetia	Spirochaetales	Spirochaetaceae	Spirochaeta	Spirochaeta	sp.	
0	0	0	33	0	0	0	0	0	0	0	0	Bacteria	Spirochaetota	Spirochaetia	Spirochaetales	Spirochaetaceae	Spirochaeta	Spirochaeta	cellobiosiphila	
0	0	0	36	0	0	0	0	0	0	0	0	Bacteria	Spirochaetota	Spirochaetia	Spirochaetales	Spirochaetaceae	Spirochaeta	Spirochaeta	sp.	
0	0	0	103	0	0	0	0	0	0	0	0	Bacteria	Spirochaetota	Spirochaetia	Spirochaetales	Spirochaetaceae	Spirochaeta	Spirochaeta	sp.	
26	138	33	368	362	287	0	96	378	305	379	289	Bacteria	Spirochaetota	Spirochaetia	Spirochaetales	Spirochaetaceae	Spirochaeta	Spirochaeta	sp.	
0	0	0	23	107	47	0	0	49	85	48	55	Bacteria	Spirochaetota	Spirochaetia	Spirochaetales	Spirochaetaceae	Spirochaeta	Spirochaeta	sp.	
0	0	0	6	0	0	0	0	0	0	0	0	Bacteria	Spirochaetota	Spirochaetia	Spirochaetales	Spirochaetaceae	Treponema	Treponema	isoptericolens	
0	0	0	77	35	42	0	0	0	32	11	0	Bacteria	Spirochaetota	Leptospirae	Leptospirales	Leptospiraceae				RBG-16-49-21
0	0	0	0	0	15	0	0	0	0	0	0	Bacteria	Spirochaetota	Leptospirae	Leptospirales	Leptospiraceae				
0	0	0	77	0	39	0	0	0	0	0	0	Bacteria	Spirochaetota	Spirochaetia	Spirochaetales	Spirochaetaceae				
0	0	0	31	37	73	0	44	125	48	53	154	Bacteria	Spirochaetota	Spirochaetia	Spirochaetales	Spirochaetaceae				
0	0	0	120	0	0	94	0	0	0	0	15	Bacteria	Spirochaetota	Spirochaetia	Spirochaetales	Spirochaetaceae				
0	0	0	18	0	0	0	0	0	0	0	0	Bacteria	Spirochaetota	Spirochaetia	Spirochaetales	Spirochaetaceae				GWE2-31-10
0	0	0	0	0	42	13	0	0	0	0	14	Bacteria	Spirochaetota	Spirochaetia	Spirochaetales	Spirochaetaceae				
0	0	0	0	100	0	0	0	0	24	0	5	Bacteria	Spirochaetota	Spirochaetia	Spirochaetales	Spirochaetaceae				
0	0	0	65	0	0	0	0	0	0	0	0	Bacteria	Spirochaetota	MVP-15	unclassified_Bacteria	unclassified_Bacteria				
0	0	0	29	0	0	0	0	0	0	0	0	Bacteria	Spirochaetota	V2072-189E03	unclassified_Bacteria	unclassified_Bacteria				
0	0	0	0	0	15	0	0	13	10	14	0	Bacteria	Sumerlaeota	Sumerlaeia	Sumerlaeales	Sumerlaeaceae	Sumerlaea	Sumerlaea	sp.	
0	0	0	0	0	0	0	0	0	7	0	0	Bacteria	Sumerlaeota	Sumerlaeia	Sumerlaeales	Sumerlaeaceae	Sumerlaea	Sumerlaea	sp.	
0	0	0	0	13	13	0	0	0	11	18	0	Bacteria	Sumerlaeota	Sumerlaeia	Sumerlaeales	Sumerlaeaceae	Sumerlaea	Sumerlaea	sp.	
0	0	0	0	14	0	0	0	0	0	0	0	Bacteria	Sumerlaeota	Sumerlaeia	Sumerlaeales	Sumerlaeaceae	Sumerlaea	Sumerlaea	sp.	
0	0	0	0	10	16	0	0	0	14	0	0	Bacteria	Sumerlaeota	Sumerlaeia	Sumerlaeales	Sumerlaeaceae	Sumerlaea	Sumerlaea	sp.	
0	0	0	0	0	0	0	0	10	0	0	0	Bacteria	Sumerlaeota	Sumerlaeia	Sumerlaeales	Sumerlaeaceae	Sumerlaea	Sumerlaea	sp.	PBS-II-1
0	0	0	0	25	24	0	0	0	49	0	0	Bacteria	Sumerlaeota	Sumerlaeia	unclassified_Bacteria	unclassified_Bacteria				
0	0	0	0	0	0	0	0	0	0	17	0	Bacteria	Sumerlaeota	Sumerlaeia	unclassified_Bacteria	unclassified_Bacteria				
10	0	0	0	79	108	0	18	0	25	41	0	Bacteria	Sumerlaeota	Sumerlaeia	unclassified_Bacteria	unclassified_Bacteria				
0	0	0	0	0	0	0	0	0	0	0	31	Bacteria	Sva0485	unclassified_Bacteria	unclassified_Bacteria	unclassified_Bacteria				
0	0	0	0	0	44	0	0	0	0	0	0	Bacteria	Sva0485	unclassified_Bacteria	unclassified_Bacteria	unclassified_Bacteria				
0	0	0	0	10	0	0	0	0	0	0	0	Bacteria	Sva0485	unclassified_Bacteria	unclassified_Bacteria	unclassified_Bacteria				
0	0	0	0	0	5	0	0	0	0	0	0	Bacteria	Sva0485	unclassified_Bacteria	unclassified_Bacteria	unclassified_Bacteria				
0	0	0	0	0	26	0	0	0	20	0	0	Archaea	Thermoplasmatota	Thermoplasmata	unclassified_Archaea	unclassified_Archaea	Marine benthic group D and DHVEG-1			
0	0	0	0	0	12	0	0	0	0	0	0	Bacteria	Thermotogota	Thermotogae	Petrotogales	Petrotogaceae	SC103			
0	0	0	0	0	0	0	24	0	0	28	0	Bacteria	Verrucomicrobiota	Verrucomicrobiae	Chthoniobacterales	unclassified_Bacteria	01D2Z36			
0	0	0	0	0	0	0	0	17	0	0	0	Bacteria	Verrucomicrobiota	Omnitrophia	Omnitrophales	Omnitrophaceae	Candidatus Omnitrophus			
0	0	0	0	0	0	0	0	0	0	0	4	Bacteria	Verrucomicrobiota	Omnitrophia	Omnitrophales	Omnitrophaceae	Candidatus Omnitrophus			
0	0	0	0	0	0	0	0	0	12	0	0	Bacteria	Verrucomicrobiota	Omnitrophia	Omnitrophales	Omnitrophaceae	Candidatus Omnitrophus			
0	0	0	0	0	0	0	4	4	0	0	0	Bacteria	Verrucomicrobiota	Verrucomicrobiae	Opitutales	Puniceicoccaceae	Cerasicoccus	Cerasicoccus	sp.	
40	22	0	27	33	43	0	31	141	58	129	54	Bacteria	Verrucomicrobiota	Verrucomicrobiae	Opitutales	Puniceicoccaceae	Cerasicoccus	Cerasicoccus	sp.	
0	0	0	37	0	0	0	0	0	0	0	0	Bacteria	Verrucomicrobiota	Verrucomicrobiae	Opitutales	Puniceicoccaceae	Coraliomargarita	Coraliomargarita	sp.	
0	0	0	0	0	0	0	0	20	0	0	0	Bacteria	Verrucomicrobiota	Verrucomicrobiae	Opitutales	Puniceicoccaceae	Coraliomargarita	Coraliomargarita	sp.	
0	0	0	96	0	0	0	0	0	0	0	0	Bacteria	Verrucomicrobiota	Verrucomicrobiae	Opitutales	Puniceicoccaceae	Coraliomargarita	Coraliomargarita	sp.	
0	0	0	0	7	0	0	0	0	0	10	0	Bacteria	Verrucomicrobiota	Chlamydiae	Chlamydiales	unclassified_Bacteria	cvE6			
0	0	0	0	0	0	0	10	0	0	0	0	Bacteria	Verrucomicrobiota	Verrucomicrobiae	Verrucomicrobiales	DEV007	DEV007			
0	0	0	0	0	0	0	0	19	0	0	0	Bacteria	Verrucomicrobiota	Verrucomicrobiae	Verrucomicrobiales	DEV007	DEV007			
0	33	0	0	0	0	0	0	0	0	0	0	Bacteria	Verrucomicrobiota	Verrucomicrobiae	Verrucomicrobiales	Rubritaleaceae	Haloferula	Haloferula	sp.	
0	0	0	39	0	0	0	0	0	0	0	0	Bacteria	Verrucomicrobiota	Verrucomicrobiae	Verrucomicrobiales	Rubritaleaceae	Haloferula	Haloferula	sp.	
32	0	0	26	0	0	0	0	0	0	0	0	Bacteria	Verrucomicrobiota	Verrucomicrobiae	Verrucomicrobiales	Rubritaleaceae	Haloferula	Haloferula	sp.	
0	0	0	0	0	0	0	0	0	6	0	0	Bacteria	Verrucomicrobiota	Omnitrophia	Omnitrophales	unclassified_Bacteria	koll11			
40	168	0	0	0	0	0	0	0	0	0	0	Bacteria	Verrucomicrobiota	Verrucomicrobiae	Opitutales	Puniceicoccaceae	Lentimonas	Lentimonas	sp.	
90	0	0	51	0	0	0	0	0	0	0	0	Bacteria	Verrucomicrobiota	Verrucomicrobiae	Opitutales	Puniceicoccaceae	Lentimonas	Lentimonas	sp.	
0	0	0	0	0	13	0	0	0	0	0	0	Bacteria	Verrucomicrobiota	Kiritimatiellae	Kiritimatiellales	Kiritimatiellaceae	MSBL3			
0	0	0	0	37	16	0	0	0	0	0	31	Bacteria	Verrucomicrobiota	Omnitrophia	Omnitrophales	Omnitrophaceae	Omnitrophus	Omnitrophus	magneticus	
0	0	0	0	0	0	0	0	27	0	0	0	Bacteria	Verrucomicrobiota	Verrucomicrobiae	Pedosphaerales	Pedosphaeraceae	Pedosphaera	Pedosphaera	sp.	
0	0	0	0	0	0	0	22	0	0	0	0	Bacteria	Verrucomicrobiota	Verrucomicrobiae	Opitutales	Puniceicoccaceae	Pelagicoccus	Pelagicoccus	sp.	
0	0	0	9	0	0	0	0	0	0	0	5	Bacteria	Verrucomicrobiota	Verrucomicrobiae	Opitutales	Puniceicoccaceae	Puniceicoccus	Puniceicoccus	sp.	
0	0	0	12	0	0	0	0	19	0	0	11	Bacteria	Verrucomicrobiota	Verrucomicrobiae	Opitutales	Puniceicoccaceae	Puniceicoccus	Puniceicoccus	sp.	
0	0	0	18	0	0	0	0	0	0	11	0	Bacteria	Verrucomicrobiota	Verrucomicrobiae	Opitutales	Puniceicoccaceae	Puniceicoccus	Puniceicoccus	sp.	
0	0	0	0	18	40	0	0	0	0	0	0	Bacteria	Verrucomicrobiota	Kiritimatiellae	Kiritimatiellales	Kiritimatiellaceae	R76-B128			
0	0	0	11	0	0	0	0	0	0	0	0	Bacteria	Verrucomicrobiota	Verrucomicrobiae	Verrucomicrobiales	Rubritaleaceae	Roseibacillus	Roseibacillus	sp.	
0	0	0	0	0	0	0	6	0	0	0	0	Bacteria	Verrucomicrobiota	Verrucomicrobiae	Pedosphaerales	Pedosphaeraceae	SCGC AAA164-E04			
0	0	0	0	0	0	11	0	57	0	0	0	Bacteria	Verrucomicrobiota	Verrucomicrobiae	Pedosphaerales	Pedosphaeraceae	SH3-11			
0	0	0	0	0	0	0	0	0	0	0	16	Bacteria	Verrucomicrobiota	Chlamydiae	Chlamydiales	Simkaniaceae	Simkania	Simkania	negevensis	Z
0	0	0	0	8	0	0	0	0	0	0	47	Bacteria	Verrucomicrobiota	Lentisphaeria	unclassified_Bacteria	unclassified_Bacteria	SS1-B-02-17			
0	0	0	0	0	0	0	0	22	56	16	6	Bacteria	Verrucomicrobiota	Verrucomicrobiae	Opitutales	Puniceicoccaceae	Verruc-01			
0	0	0	0	0	0	33	0	0	0	0	0	Bacteria	Verrucomicrobiota	Verrucomicrobiae	Opitutales	Puniceicoccaceae	Verruc-01			
0	0	0	0	0	0	0	0	0	9	0	0	Bacteria	Verrucomicrobiota	Verrucomicrobiae	Opitutales	Puniceicoccaceae	Verruc-01			
0	0	0	48	0	12	11	31	115	10	20	57	Bacteria	Verrucomicrobiota	Verrucomicrobiae	Opitutales	Puniceicoccaceae	Verruc-01			
0	0	0	0	0	0	0	0	0	0	0	2	Bacteria	Verrucomicrobiota	Chlamydiae	Chlamydiales	Waddliaceae	Waddlia	Waddlia	chondrophila	WSU-86-1044
0	0	0	0	0	32	8	0	0	0	0	0	Bacteria	Verrucomicrobiota	Kiritimatiellae	unclassified_Bacteria	unclassified_Bacteria	WCHB1-41			
0	20	0	26	0	0	0	0	0	30	0	0	Bacteria	Verrucomicrobiota	Kiritimatiellae	unclassified_Bacteria	unclassified_Bacteria	WCHB1-41			
0	0	0	0	14	0	0	0	0	0	0	9	Bacteria	Verrucomicrobiota	Chlamydiae	Chlamydiales	unclassified_Bacteria				
0	0	0	40	0	0	0	0	0	0	0	0	Bacteria	Verrucomicrobiota	Verrucomicrobiae	Chthoniobacterales	unclassified_Bacteria				
0	0	0	0	5	0	0	0	0	0	0	0	Bacteria	Verrucomicrobiota	Verrucomicrobiae	Methylacidiphilales	Methylacidiphilaceae				
0	0	0	47	0	0	0	0	0	9	0	0	Bacteria	Verrucomicrobiota	Verrucomicrobiae	Methylacidiphilales	Methylacidiphilaceae				
0	0	0	43	0	0	0	6	0	0	17	0	Bacteria	Verrucomicrobiota	Verrucomicrobiae	Opitutales	Puniceicoccaceae				
42	75	0	95	0	62	0	23	13	11	5	0	Bacteria	Verrucomicrobiota	Verrucomicrobiae	Opitutales	Puniceicoccaceae				
0	0	0	0	0	0	0	0	0	0	26	0	Bacteria	Verrucomicrobiota	Verrucomicrobiae	Pedosphaerales	Pedosphaeraceae				
0	0	0	15	0	0	0	18	0	6	0	0	Bacteria	WPS-2	unclassified_Bacteria	unclassified_Bacteria	unclassified_Bacteria				
0	0	0	0	6	0	0	0	0	0	0	0	Bacteria	WS2	unclassified_Bacteria	unclassified_Bacteria	unclassified_Bacteria				
0	0	0	0	122	74	0	27	40	0	98	0	Bacteria	Zixibacteria	unclassified_Bacteria	unclassified_Bacteria	unclassified_Bacteria				
0	0	0	0	13	0	0	0	36	4	45	5	Bacteria	Zixibacteria	unclassified_Bacteria	unclassified_Bacteria	unclassified_Bacteria				
0	0	0	0	0	0	59	0	14	0	0	58	Bacteria	Zixibacteria	unclassified_Bacteria	unclassified_Bacteria	unclassified_Bacteria				
0	0	0	0	81	0	0	0	0	0	0	6	Bacteria	Zixibacteria	unclassified_Bacteria	unclassified_Bacteria	unclassified_Bacteria				
0	0	0	0	0	18	0	0	0	0	0	0	Bacteria	Zixibacteria	unclassified_Bacteria	unclassified_Bacteria	unclassified_Bacteria				
